# Bismuth(III) triflate: an economical and environmentally friendly catalyst for the Nazarov reaction

**DOI:** 10.3762/bjoc.20.99

**Published:** 2024-05-21

**Authors:** Manoel Trindade Rodrigues Jr., Aline Silva Barroso de Oliveira, Ralph C Gomes, Amanda Soares Hirata, Lucas A Zeoly, Hugo Santos, João Arantes, Catarina Sofia Mateus Reis-Silva, João Agostinho Machado-Neto, Leticia Veras Costa-Lotufo, Fernando Coelho

**Affiliations:** 1 Institute of Chemistry, Universidade Estadual de Campinas, PO Box 6154, 13083-970 Campinas, São Paulo, Brazil; 2 Department of Pharmacology, Institute of Biomedical Sciences, University of São Paulo, 05508-000 São Paulo, Brazil

**Keywords:** bismuth, catalysis, heterocycles, indanones, Nazarov reaction

## Abstract

We describe the use of bismuth(III) triflate as an efficient and environmentally friendly catalyst for the Nazarov reaction of aryl vinyl ketones, leading to the synthesis of 3-aryl-2-ethoxycarbonyl-1-indanones and 3-aryl-1-indanones. By changing the temperature and reaction time, it was possible to modulate the reactivity, allowing the synthesis of two distinct product classes (3-aryl-2-ethoxycarbonyl-1-indanones and 3-aryl-1-indanones) in good to excellent yield. The reaction did not require additives and was insensitive to both air and moisture. Preliminary biological evaluation of some indanones showed a promising profile against some human cancer line cells.

## Introduction

**Figure 1:**
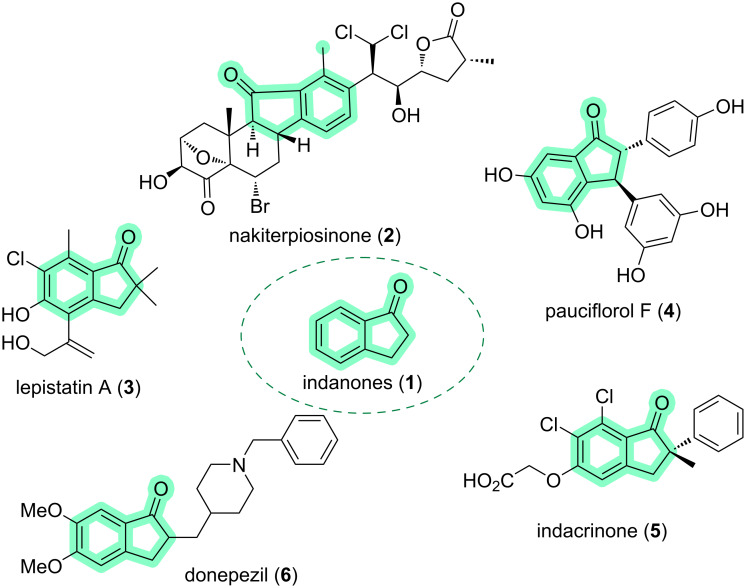
Examples of different compounds containing the indanone moiety.

Natural products are the source of inspiration for several research groups that develop new synthetic methodologies. The chemistry of five-membered rings plays an important role in organic chemistry, both because of the wide occurrence in nature [1–6] and the broad spectrum of biological activities. Among the five-membered ring compounds, we find indanone (**1**, Figure 1). Within the class of indanones, we can highlight some interesting compounds. For example, nakiterpiosinone (**2**), which inhibits the growth of P388 mouse leukemia cells with an average inhibitory concentration (IC_50_) of 10 ng/mL, lepistatin A (**3**), along with two other new chlorinated analogs, that were isolated from Basidiomycete *Lepista sordida* culture, pauciflorol F (**4**), isolated from *Vatica pauciflora*, which is an important building block for the biosynthesis of bioactive polyphenols, in addition to having antiviral activity, indacrinone (**5**), which is related to ethacrynic acid and usually stimulates the reversible short-circuit current and the influx of sodium when applied to the epithelial surface of amphibian skin, and donepezil (**6**), a drug used to treat Alzheimer's disease [7–12].

The interest in the preparation of functionalized indanone derivatives has increased enormously, and many synthetic methods have been developed, including Friedel–Crafts cyclization reactions [13], cyclization of acetylenic derivatives [14], ring contractions and ring expansions [15], and the Nazarov reaction [16–20].

The Nazarov cyclization is one of the most versatile and simple methods for preparing indanones from aryl vinyl ketone derivatives [16–20]. The Nazarov reaction is classically formulated as a 4π conrotatory electrocyclization of a pentadienyl cation [1–12]. Until the past decade, the conditions used for the Nazarov reaction generally involved the use of a stoichiometric amount of a strong Lewis acid (e.g., BF_3_, TiCl_4_, SnCl_4_, AlCl_3_) in relation to the divinyl ketone derivative [21–23]. However, Dhoro and Tius demonstrated that weak acids could also be used as efficient catalysts for the Nazarov reaction [24].

In this context, some research groups developed methodologies that allowed the use of a catalytic amount of Lewis acid. By using more reactive divinyl ketone derivatives, the electrocyclization reaction could be mediated by weaker Lewis acids, and consequently a catalytic amount of them could be used. The first example of a catalytic version of the Nazarov cyclization was reported by Denmark and Jones [25–31]. They found that a substoichiometric amount of FeCl_3_ (40–50 mol %) promoted the cyclization of silylated derivatives efficiently. However, when 10 mol % was used, the conversion was poor. Denmark’s and Jones’s pioneering work was used as inspiration for the development of catalytic methodologies for this reaction. In 2004, Lang and Trauner described the first asymmetric catalytic Nazarov reaction [32]. In recent years, several strategies were reported employing different Lewis acids, such as, AuCl_3_/AgSbF_6_, Cu(II), In(OTf)_3_, Ir(III), Al(III), Sc(OTf)_3_/LiClO_4_, In(OTf)_3_/diphenylphosphoric acid (DPP), Fe(OTf)_3_/(CF_3_)_2_PhB(OH)_2_, iodine [33–43], and other strategies [44–45].

Although methodologies involving catalysis by Lewis acids are very efficient, including asymmetric versions of the Nazarov reaction, the experimental protocols are quite laborious in most cases, requiring low temperature, an inert atmosphere, or the use of Lewis acids sensitive to moisture [2,27–28,30–31,33–34,36,40–43,46–49].

Despite the numerous reports on catalytic versions of the Nazarov reaction, few of them describe the use of bismuth salts as catalysts for Nazarov-type reactions [50–51] and none for the classical Nazarov reaction. With the growing environmental concern and the need to use green reagents, interest in the use of bismuth in organic synthesis has increased significantly, as is reflected by the large number of works dedicated to this topic [52–54]. In addition to the replacement of toxic heavy metals, the use of bismuth compounds to promote reactions has the advantages of low cost and insensitivity to water and air. As such, the handling does not require special experimental techniques, such as an inert atmosphere and anhydrous solvents [55]. The use of bismuth salts in organic synthesis has been reported for several transformations, such as epoxide opening [56], ketal formation and deprotection [57–58], Mannich reaction [59], intramolecular Sakurai cyclization [60], alcohol oxidation [61], aromatic hydrocarbon nitration [62], imine allylation [63], Knoevenagel condensation [64], Reformatsky reaction [65], azalactone synthesis [66], nitro reduction [67–68], epoxide rearrangement, thiourea guanylation, and others [69–70].

In this article, we describe a simple and direct protocol for the preparation of indanones through a classical Nazarov reaction catalyzed by bismuth(III) triflate. In addition to the synthetic simplicity, the moisture stability of bismuth triflate allows the protocol to be carried out under ambient atmospheric conditions.

## Results and Discussion

### Preparation of β-ketoesters

**Figure 2:**
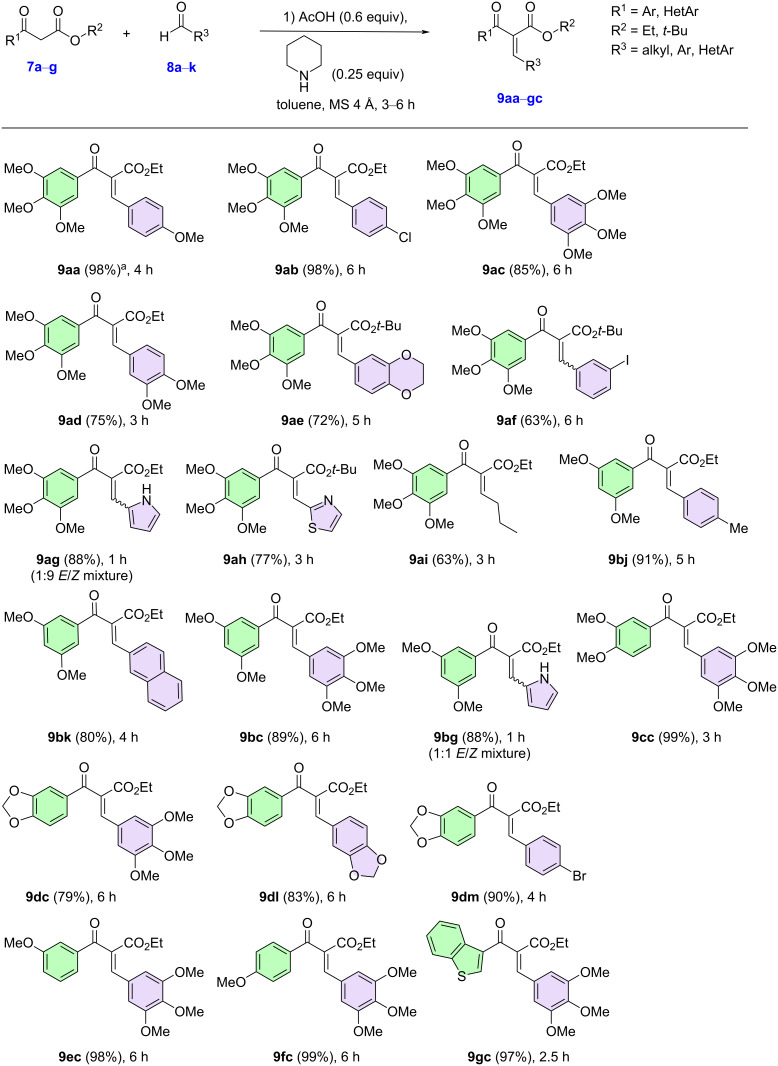
Synthesis of unsaturated β-ketoesters (Knoevenagel derivatives). ^a^Isolated yield after purification using silica gel column chromatography.

We initiated our studies with the preparation of the β-ketoesters, which were synthesized according to well-established protocols [71–74]. The β-ketoesters were obtained employing a sequence of two reactions, the formation of the benzylic alcohol derivative, through a Reformatsky reaction using In(0), followed by a pyridinium chlorochromate (PCC) oxidation, giving the β-ketoesters **7a**–**g** in moderate to good yields. With the β-ketoesters prepared, we began the synthesis of the Knoevenagel derivatives. To do so, we employed an adapted protocol from the literature. Using 1.00 equiv of β-ketoester, 1.50 equiv of aldehyde, 0.60 equiv of acetic acid, and 0.25 equiv of piperidine, the desired products were obtained in good to excellent yields and *E*-selectivities (Figure 2) [71–74]. Various substrates (**9aa**–**gc**), containing electron-donating, electron-withdrawing, electroneutral groups, and heteroaryl units (2-thiophene and 3-benzothiophene) were obtained in good to excellent yields. For derivatives **9af**,**ag**,**bq**, we observed the formation of an *E*/*Z* mixture of products that was inseparable by column chromatography.

### Synthesis of 3-aryl-2-ethoxycarbonyl-1-indanones and 3-aryl-1-indanones

With the starting materials prepared, we began evaluating the use of bismuth salts to promote the Nazarov reaction, using models already studied in the literature [33–43]. We investigated several conditions, such as the type of catalyst, temperature, solvent, and amount of catalyst (Table 1). Our optimization studies began with the reaction of substrate **9aa** with Bi(OTf)_3_ (10 mol %) in acetonitrile at room temperature (Table 1, entry 1). The desired product was obtained in 72% after 12 h. When the reaction was carried out at 40 °C for 8 h, the yield increased slightly to 76% (Table 1, entry 2). When the reaction was carried out at 60 °C, the yield increased to 93%, in addition to a marked decrease in the reaction time (Table 1, entry 3). Despite this excellent result, we continued to evaluate other catalysts to improve the yield and reaction time further. For this purpose, a series of Lewis acids, such as Bi(NO)_3_, BiBr_3_, BiCl_3_, Yt(OTf)_3_, Dy(OTf)_3_, ZrCl_4_, In(OTf)_3_, InCl_3_, and AlCl_3_ were selected as catalysts (Table 1, entries 5–13), and even after this screening, the best result still remained the one obtained with Bi(OTf)_3_. The Brønsted acids TFA and TsOH were also tested for the transformation but gave worse results (Table 1, entries 14 and 15). Once the catalyst was chosen, we investigated the influence of the solvent. For this purpose, we evaluated dichloroethane (DCE), dichloromethane (DCM), toluene, and tetrahydrofuran (THF) as solvents for the transformation (Table 1, entries 16–19), but acetonitrile remained the best solvent. Finally, we evaluated the amount of catalyst, using two different catalyst loadings (5 and 20 mol %), but these variations also did not improve the yield (Table 1, entries 20 and 21). After this screening, the optimum conditions employed 10 mol % of bismuth triflate (Bi(OTf)_3_) in acetonitrile at 60 °C (Table 1, entry 3).

**Table 1:** Optimization of the reaction conditions.

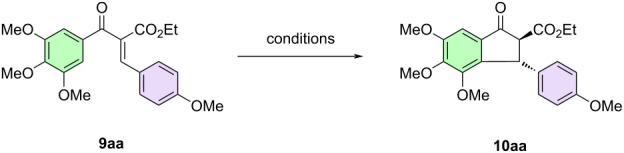					

entry	catalyst (mol %)	solvent	*T* (°C)	*t* (h)	yield (%)^a^

1	Bi(OTf)_3_ (10)	acetonitrile	25	12	72
2	Bi(OTf)_3_ (10)	acetonitrile	40	8	76
3	**Bi(OTf)** **_3_** ** (10)**	**acetonitrile**	**60**	**2**	**93**
4	Bi(OTf)_3_ (10)	acetonitrile	70	2	88
5	Bi(NO)_3_ (10)	acetonitrile	60	24	15^b^
6	BiBr_3_ (10)	acetonitrile	60	24	26^b^
7	BiCl_3_ (10)	acetonitrile	60	12	24^b^
8	Yt(OTf)_3_(10)	acetonitrile	60	24	15^b^
9	Dy(OTf)_3_ (10)	acetonitrile	60	12	45
10	ZrCl_4_ (10)	acetonitrile	60	12	58
11	In(OTf)_3_ (10)	acetonitrile	60	2	71
12	InCl_3_ (10)	acetonitrile	60	12	30
13	AlCl_3_ (10)	acetonitrile	60	24	5^b^
14	TFA (10)	acetonitrile	60	6	58
15	TsOH (10)	acetonitrile	60	2	65
16	Bi(OTf)_3_ (10)	DCE	60	1	65
17	Bi(OTf)_3_ (10)	DCM	60	1	73
18	Bi(OTf)_3_ (10)	toluene	60	1.5	67
19	Bi(OTf)_3_ (10)	THF	60	3	53
20	Bi(OTf)_3_ (5)	acetonitrile	60	2	87
21	Bi(OTf)_3_ (20)	acetonitrile	60	1.5	91
22	TfOH (10)	acetonitrile	60	1.5	83
23	Bi(OTf)_3_ (10)	DCE	60	1	35^b^

^a^Isolated yield after purification using silica gel column chromatography. ^b^Recovery of starting material.

**Figure 3:**
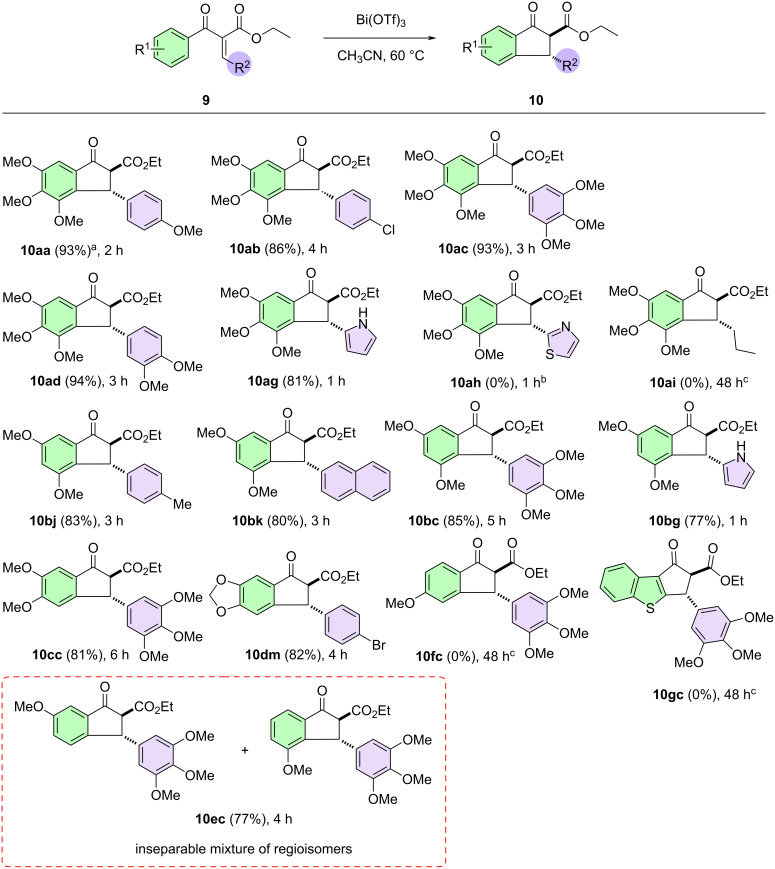
Synthesis of 3-aryl-2-ethoxycarbonyl-1-indanones mediated by bismuth triflate. ^a^Isolated yield after purification using silica gel column chromatography. ^b^Extensive degradation. ^c^Recovery of starting material. Basic protocol: The Knoevenagel product **9** (0.5 mmol), dry acetonitrile (2 mL), and Bi(OTf)_3_ (0.05 mmol) were added to a sealed tube. The reaction mixture was stirred at 60 °C and monitored by TLC.

With the optimized conditions in hand, we explored the substrate scope (Figure 3). In general, substrates with electron donor groups provided a better yield when compared to those obtained with electroneutral or electron-withdrawing groups. Particularly for substrates **9ai**,**fc**,**gc**, there was no formation of the corresponding indanones even though the reaction remained at 60 °C for a longer time (48 h), but the starting materials could be recovered. For substrate **9ah**, there was complete decomposition of the starting material, along with the formation of several byproducts.

Surprisingly, when using the optimized conditions for substrates **9dc**,**dl**, we found that in addition to the products of interest, they also formed the decarboxylated products as inseparable mixtures. Given this result, we investigated milder conditions to avoid decarboxylation. In doing so, we reacted the substrate **9dc** for 24 h at room temperature. Despite this long reaction time, we still observed formation of the decarboxylated derivative, and we could also partly recover the starting material (Table 2).

**Table 2:** Evaluation of the reactivity of **9dc**,**dl**.

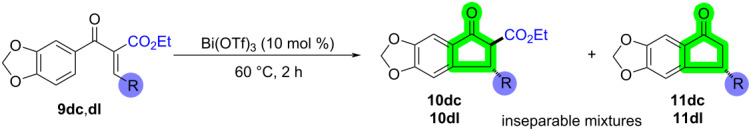		

R	yield (%^)a^	**10**/**11** ratio^b^

3,4,5-(CH_3_O)_3_C_6_H_2_ (i.e.,**9dc**)	77	8:2
piperonyl (i.e., **9dl**)	75	7:3

^a^Yield determined by ^1^H NMR analysis using dimethyl terephthalate as internal standard. ^b^Ratio determined by ^1^H NMR analysis.

**Scheme 1:**
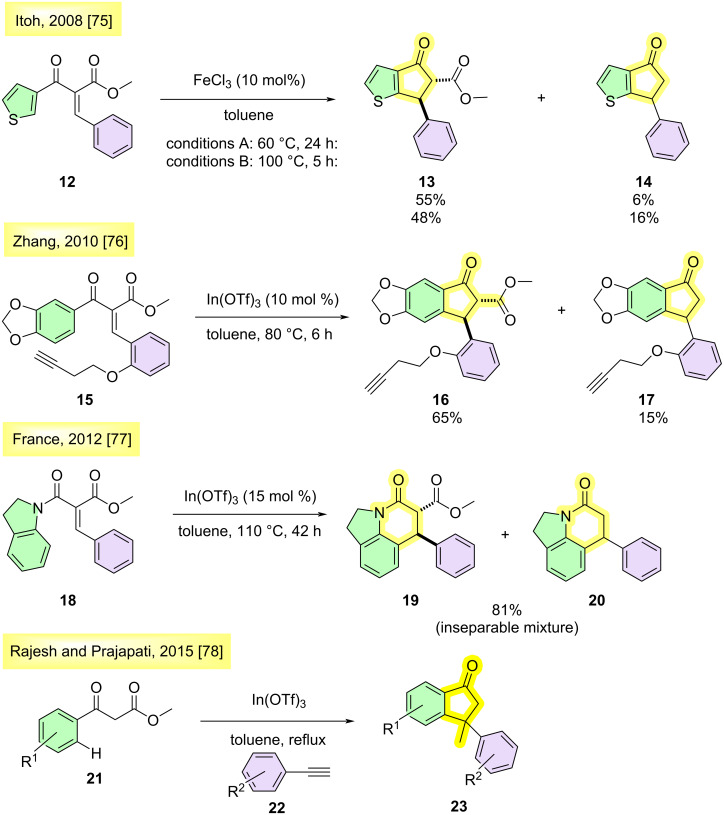
Previous methods describing decarboxylation reactions of indanones and xanthenones.

Decarboxylation reactions of indanones have previously been described in the literature. In 2008, Itoh et al. investigated the Nazarov cyclization of 3-substituted thiophene derivatives. When carrying out the reactions at 60 °C for 24 h, the formation of a 9:1 mixture of the products **13** and **14** in 61% combined yield was observed (Scheme 1) [75]. Under more vigorous conditions (100 °C for 5 h), there was a slight increase in the yield of decarboxylated product **14**. In 2010, Zhang et al. [76] developed a methodology catalyzed by In(OTf)_3_ for the synthesis of bicycles, and they also verified that decarboxylation occurred when the reaction remained at 80 °C for 6 h (Scheme 1). The same behavior was observed by France during the synthesis of the lilolidone nucleus [77] and by Jung in the stereoselective synthesis of podophyllotoxin derivatives (Scheme 1) [78]. Although the decarboxylation reaction of indanones has been observed and described in the literature, there are few extensive studies aimed at exploring this transformation. The only exception is a report by Rajesh and Prajapati from 2015 [79]. In this work, the authors aimed at obtaining substituted β,β-indanones, and the decarboxylation step was a mandatory part of the methodology, with no interest in controlling the process.

The indanone core is a privileged structure, as it is often found in a series of natural products and synthetic molecules with different biological activities [11,80–81]. In particular, 1-indanones substituted in position 3 are important synthons for some drugs and natural products [82–84]. A bibliographic survey revealed that some methods were developed for the synthesis of 3-aryl-1-indanones. In this context, due to the formation of the previously shown derivatives **11dc**,**dl**, we decided to explore the Nazarov reaction–decarboxylation sequence catalyzed by Bi(OTf)_3_ to prepare variously substituted 3-aryl-1-indanones from substrate **9**.

**Figure 4:**
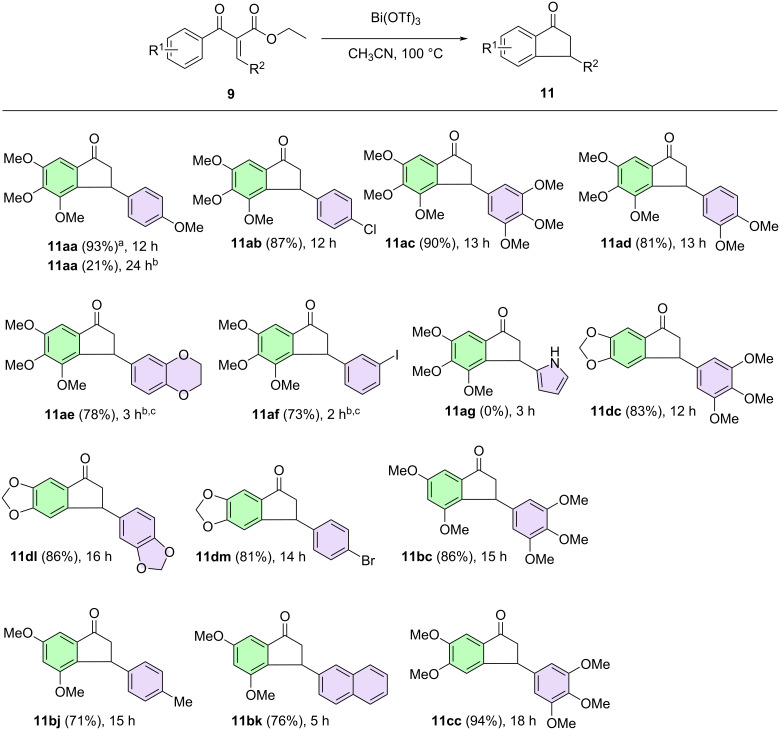
Controlled decarboxylation directed by bismuth triflate at 100 °C. Synthesis of 3-aryl-1-indanones. ^a^Isolated yield after purification using silica gel column chromatography. ^b^Reaction performed at 60 °C. ^c^From *tert*-butyl Knoevenagel derivative. Basic protocol: The Knoevenagel product **9** (0.5 mmol), dry acetonitrile (2 mL), and Bi(OTf)_3_ (0.05 mmol) were added to a sealed tube. The reaction mixture was stirred at 100 °C and monitored by TLC.

We initially investigated the behavior of substrate **9aa** under the conditions established for the Nazarov cyclization (60 °C). However, despite the reaction remaining under these conditions for a long period (24 h), only partial decarboxylation of the Nazarov product was observed. Thus, we decided to increase the temperature to 100 °C, and the reaction was maintained under these conditions in a sealed tube for 12 h. To our delight, we obtained only the decarboxylated indanone in an excellent yield (93%). These conditions were chosen as the optimal conditions, and the scope for the synthesis of 3-aryl-1-indanones derivatives is summarized in Figure 4.

Simply by controlling the reaction temperature, it was possible to obtain indanones with different substitution patterns. At the lower temperature (60 °C), 2,3-substituted indanones could be obtained, while at the higher temperature (100 °C), 3-substituted indanones were achieved. Under both conditions, virtually no product mixtures were observed.

### Preliminary biological evaluation

**Figure 5:**
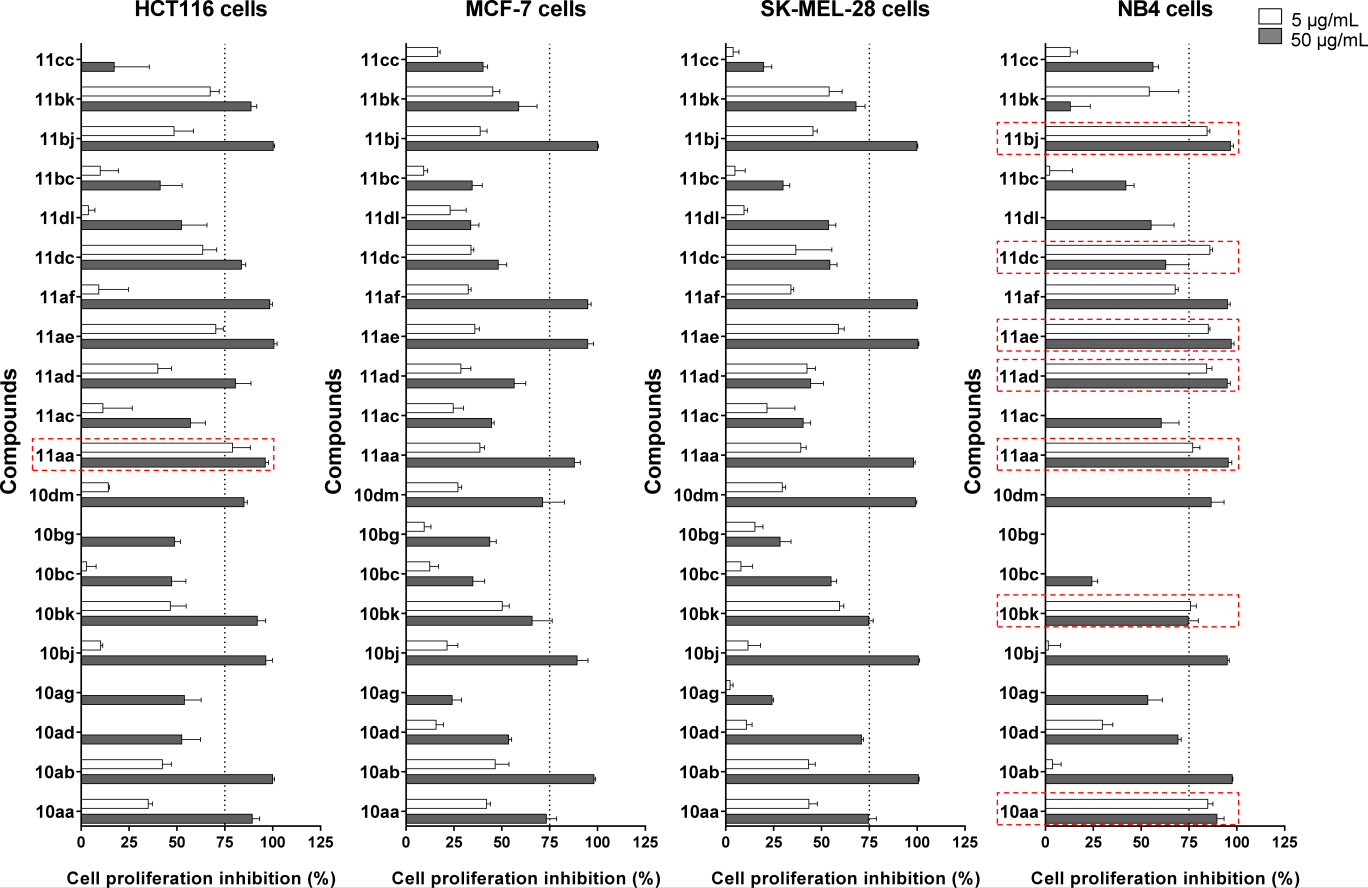
Impact of indanone derivatives on cell viability of tumor cells. Cell viability was determined by MTT assay. Data is expressed as reduction in viability in relation to the vehicle, and the dotted line indicates 75% reduction in cell viability. Compounds that reduced cell viability by at least 75% at 5 µg/mL are highlighted in the graph.

To investigate the cytotoxic activity of the indanone derivatives, in total 20 compounds were tested at a concentration of 5 and 50 μg/mL for 72 h in a panel of four histologically unrelated tumor lines, HCT116 (colon adenocarcinoma), MCF7 (breast adenocarcinoma), SK-MEL-28 (melanoma), and NB4 (acute leukemia) by methylthiazol tetrazolium (MTT) assay, as previously described [85]. Among the tested cells, NB4 cells were the most sensitive ones, with 7 compounds (**10aa**,**bk** and **11aa**,**ad**,**ae**,**dc**,**bj**) being active at 5 μg/mL, using a 75% cutoff inhibition at each concentration. On the other hand, MCF-7 and SK-MEL-28 cells were the most resistant ones, with no compound being active at the lower concentration (Figure 5). This evidence of selective cytotoxicity for a specific histological tumor subtype may drive further structure–activity relationship studies to identify indanone targets of pharmacological interest.

## Conclusion

In summary, we developed a simple and efficient methodology for the Nazarov reaction of aryl vinyl ketones, leading to 3-aryl-2-ethoxycarbonyl-1-indanones and 3-aryl-1-indanones. The reactions were catalyzed by bismuth triflate, an environmentally friendly metal. By simply changing the temperature and reaction time, it was possible to modulate the reactivity. In this methodology, no additives were used, and the reaction was insensitive to both air and moisture. To the best of our knowledge, this study is unique in the sense that there are no previous reports on the use of bismuth triflate as catalyst for a classic Nazarov reaction. There is also no reported precedent for the preparation of indanones with different substitution patterns through simple control of the reaction temperature.

The initial biological profile of 20 indanones was assessed, revealing promising activity against certain human cancer cell lines in some cases. To enhance the anticancer potential of these compounds, it is imperative to carry out additional comprehensive studies.

## Supporting Information

File 1 Experimental section and copies of ^1^H and ^13^C NMR spectra of all new compounds.

## Data Availability

The data that supports the findings of this study is available from the corresponding author upon reasonable request.
