# Supplementary material for: Bismuth(III) triflate: an economical and environmentally friendly catalyst for the Nazarov reaction
Source: Beilstein J Org Chem. 2024 May 21;20:1167–78. doi: 10.3762/bjoc.20.99 (PMC11181232; doi:10.3762/bjoc.20.99)
Supplement: File 1 — Experimental section and copies of 1H and 13C NMR spectra of all new compounds. [file Beilstein_J_Org_Chem-20-1167-s001.pdf]

BEILSTEIN JOURNAL OF ORGANIC CHEMISTRY

Supporting Information

for

Bismuth(III) triflate: an economical and environmentally friendly catalyst for the Nazarov reaction

Manoel T. Rodrigues Jr., Aline S. B. de Oliveira, Ralph C. Gomes, Amanda Soares Hirata, Lucas A. Zeoly, Hugo Santos, João Arantes, Catarina Sofia Mateus Reis-Silva, João Agostinho Machado-Neto, Leticia Veras Costa-Lotufo and Fernando Coelho

Beilstein J. Org. Chem. **2024,** *20,* 1167–1178. doi:10.3762/bjoc.20.99

Experimental section and copies of ^{1}H and ^{13}C NMR spectra of all new compounds

License and Terms: This is a supporting information file under the terms of the Creative Commons Attribution License (https://creativecommons.org/licenses/by/4.0). Please note that the reuse, redistribution and reproduction in particular requires that the author(s) and source are credited and that individual graphics may be subject to special legal provisions.
The license is subject to the *Beilstein Journal of Organic Chemistry* terms and conditions: (https://www.beilstein-journals.org/bjoc/terms)

SUPPORTING INFORMATION

S1 General information

S2 General procedures and characterization data for compounds **9aa–gc**, **10aa–dm**, and **11aa–af**

S22 References

S23 ^{1}H NMR and ^{13}C NMR spectra

General information

All chemicals and solvents were of analytical grade, purchased from commercial sources, and used without further purification unless otherwise stipulated. Unless otherwise noted, all reactions were performed under ambient atmosphere in oven-dried open-flask glassware with magnetic stirring. Reaction progress was monitored by analytical thin-layer chromatography (TLC) performed on Merck precoated silica gel 60 F254 (5–40 μm thickness) plates. The TLC plates were visualized with UV light (254 nm) and/or phosphomolybdic acid or sulfuric vanillin, followed by heating. The reaction products were purified by flash column chromatography using silica gel (230–400 mesh).

Nuclear magnetic resonance (NMR) spectra were recorded in $CDCl_3$ solution at room temperature, unless noted otherwise. ^{1}H NMR and proton-decoupled ^{13}C NMR spectra were acquired on a Bruker DPX250 (250 MHz for ^{1}H NMR and 63 MHz for ^{13}C NMR), Bruker Avance 400 (400 MHz for ^{1}H NMR and 101 MHz for ^{13}C NMR), Bruker Avance 500 (500 MHz for ^{1}H and 126 MHz for ^{13}C NMR), or Bruker Avance 600 (600 MHz for ^{1}H and 150 MHz for ^{13}C NMR). Chemical shifts (δ) are reported in ppm and the coupling constant (*J*) in Hz. Signal multiplicity was assigned as singlet (s), doublet (d), double doublet (dd), double double doublet (ddd), triplet (t), quartet (qt), double quartet (dq), multiplet (m), and broad singlet (bs). High-resolution mass spectrometry (HRMS) was performed using electrospray ionization (ESI) on a Thermo Scientific Q Exactive mass spectrometer. Melting points were obtained using a Gehaka equipment model PF 1500 FARMA and were corrected. The compounds were named according to IUPAC rules using the program MarvinSketch 15.9.21.0.

General procedures and characterization data for compounds 9aa–gc, 10aa–dm, and 11aa–af

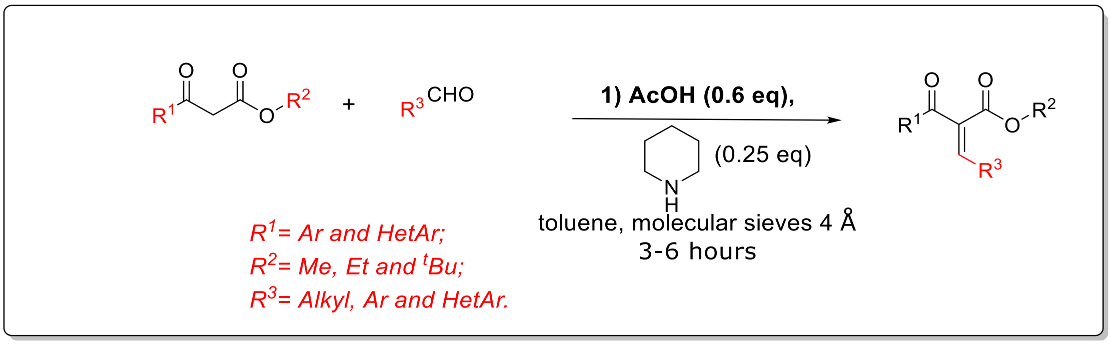

A solution of β-ketoester (1 mmol), aldehyde (1.5 mmol), acetic acid (0.6 mmol), piperidine (0.25 mmol), and molecular sieve (100 mg) in anhydrous toluene was refluxed. The reaction was monitored by TLC. After this period, the solvent was removed under reduced pressure. The crude reaction mixture was purified by column chromatography using a mobile phase of hexane/ethyl acetate 9:1–7:3, v/v.

Ethyl (2*Z*)-3-(4-methoxyphenyl)-2-[(*Z*)-3,4,5-trimethoxybenzoyl]prop-2-enoate (**9aa**)

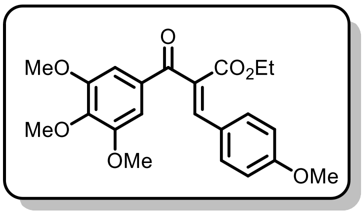

Purified by column chromatography (hexane/EtOAc 95:5–9:1) to give **9aa** (392 mg, 0,98 mmol, 98%) as a yellow solid. mp 103-104 °C. ^{1}H NMR (250 MHz, $CDCl_3$) δ 7.88 (s, 1H), 7.29 (d, *J* = 8.8 Hz, 2H), 7.20 (s, 2H), 6.75 (d, *J* = 8.9 Hz, 2H), 4.22 (q, *J* = 7.1 Hz, 2H), 3.89 (s, 3H), 3.81 (s, 6H), 3,74 (s, 3H), 1.19 (t, *J* = 7.1 Hz, 3H). ^{13}C NMR (63 MHz, $CDCl_3$) δ 194.98, 165.50, 161.54, 153.40, 143.42, 142.40, 132.35, 131.50, 128.55, 125.63, 114.47, 106.65, 61.52, 61.04, 56.38, 55.43, 14.30. IR (ATR, ν_{max}) 3403, 2918, 1589, 1455, 1321, 835, 747, 728 cm^{-1}. HRMS (ESI) *m/z* calcd for $C_{22}H_{25}O_7^+$ [M + H]$^+$ 401.1595, found 401.1601.

Ethyl (2*Z*)-3-(4-chlorophenyl)-2-[(*Z*)-3,4,5-trimethoxybenzoyl]prop-2-enoate (**9ab**)

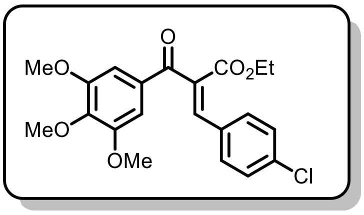

Purified by column chromatography (hexane/EtOAc 95:5–9:1) to give **9ab** (396 mg, 0,98 mmol, 98%) as a yellow solid. mp 87-89 °C (lit. 97-98 °C). ^{1}H NMR (400 MHz, Acetone) δ 8.00 (s, 1H), 7.92 (s, 1H), 7.44 (d, *J* = 8.6 Hz, 2H), 7.38 – 7.30 (m, 2H), 7.24 (s, 2H), 4.24 (p, *J* = 7.0 Hz, 2H), 3.82 (s, 6H), 3.79 (s, 3H), 1.20 (t, *J* = 7.0 Hz, 3H). ^{13}C NMR (101 MHz, Acetone) 193.25, 164.40, 153.62, 143.67, 140.25, 135.68, 132.57, 132.16, 131.48, 131.29, 128.94, 106.52, 61.19, 59.88, 55.75, 13.59. IR (ATR, ν_{max}) 3403, 2918, 1589, 1455, 1321, 835, 747, 728 cm^{-1}. HRMS (ESI) *m/z* calcd for $C_{21}H_{22}ClO_6^+$ [M + H]$^+$ 405.1099, found 405.1105.

Ethyl 2-[(*Z*)-3,4,5-trimethoxybenzoyl]-3- (3,4,5-trimethoxyphenyl)prop-2-enoate (**9ac**)

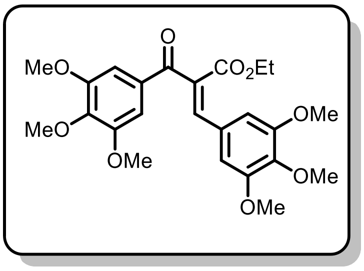

Purified by column chromatography (hexane/EtOAc 95:5–9:1) to give **9ac** (391 mg, 0,85 mmol, 85%) as a white solid. mp 77-78 °C. ^{1}H NMR (500 MHz, $CDCl_3$) δ 7.82 (s, 1H), 7.19 (s, 2H), 6.55 (s, 2H), 4.35 – 4.09 (m, 2H), 3.86 (s, 3H), 3.79 (s, 6H), 3.76 (s, 3H), 3.62 (s, 6H), 1.19 (t, *J* = 7.1 Hz, 3H). ^{13}C NMR (126 MHz, $CDCl_3$) δ 194.46, 165.04, 153.35, 153.06, 143.44, 142.41, 140.02, 131.39, 130.23, 128.14, 107.70, 106.41, 61.58, 60.95, 60.85, 56.31, 55.89, 14.18. HRMS (ESI) *m/z* calcd for $C_{24}H_{29}O_9^+$ [M + H]$^+$ 461.1806, found 461.1823.

Ethyl (2*Z*)-3-(3,4-dimethoxyphenyl)-2-[(*Z*)-3,4,5-trimethoxybenzoyl]prop-2-enoate (**9ad**)

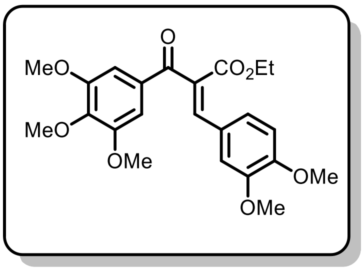

Purified by column chromatography (hexane/EtOAc 95:5–9:1) to give **9ad** (322 mg, 0,75 mmol, 75%) as a yellow oil.^{1}H NMR (400 MHz, $CDCl_3$) δ 7.85 (s, 1H), 7.19 (s, 2H), 6.96 (dd, *J* = 8.4, 2.0 Hz, 1H), 6.80 (d, *J* = 2.0 Hz, 1H), 6.75 – 6.69 (m, 1H), 4.27 – 4.13 (m, 2H), 3.87 (s, 3H), 3.80 (s, 3H), 3.78 (s, 6H), 3,59 (s, 3H), 1.19 (t, *J* = 7.1 Hz, 3H). ^{13}C NMR (101 MHz, $CDCl_3$) δ 195.01, 165.46, 153.46, 151.24,

148.92, 143.51, 142.62, 131.56, 128.73, 125.84, 125.12, 112.58, 111.12, 106.60, 61.60, 61.10, 56.43, 56.02, 55.73, 14.34. HRMS (ESI) *m/z* calcd for $C_{23}H_{27}O_8^+$ $[M + H]^+$ 431.1700, found 431.1708.

Tert-butyl (2*Z*)-3-(2,3-dihydro-1,4-benzodioxin-6-yl)-2-[(*Z*)-3,4,5-trimethoxybenzoyl]prop-2-enoate (**9ae**)

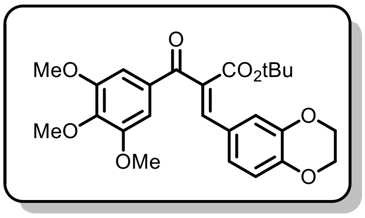

Purified by column chromatography (hexane/EtOAc 95:5–9:1) to give **9ae** (327 mg, 0,72 mmol, 72%) as a yellow oil. ^{1}H NMR (500 MHz, $CDCl_3$) δ7.72 (s, 1H), 7.21 (s, 2H), 6.93 – 6.86 (m, 2H), 6.75 (d, *J* = 8.3 Hz, 1H), 4.29 – 4.16 (m, 4H), 3.93 (s, 3H), 3.86 (s, 6H), 1.39 (s, 9H).

^{13}C NMR (126 MHz, $CDCl_3$) δ 194.99, 164.56, 153.37, 145.84, 143.61, 143.14, 141.58, 132.09, 130.95, 126.66, 124.51, 119.32, 117.82, 106.41, 82.19, 64.69, 64.24, 61.13, 56.43, 28.10. HRMS (ESI) *m/z* calcd for $C_{25}H_{29}O_8^+$ $[M + H]^+$ 457.1857, found 457.1862.

Tert-butyl (2)-3-(3-iodophenyl)-2-[-3,4,5-trimethoxybenzoyl]prop-2-enoate (**9af**)

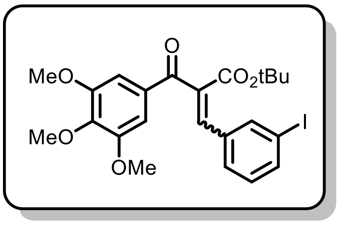

Purified by column chromatography (hexane/EtOAc 95:5–8:2) to give **9af** (330 mg, 0,63 mmol, 63%) as a brown oil. *E*/*Z* ≈ 1:3. ^{1}H NMR (500 MHz, $CDCl_3$) δ 7.73 (s, 1H), 7.71 (t, *J* = 1.5 Hz, 1H), 7.62 (d, *J* = 8.0 Hz, 1H), 7.32 (s, 1H), 7.30 (s, 1H), 7.28 (s, 1H), 7.17 (s, 2H), 6.99 (t, *J* = 7.9 Hz, 1H), 3.92 (s, 3H), 3.87 (s, 6H), 1.43 (s, 9H). ^{13}C NMR (126 MHz, $CDCl_3$) δ194.07, 163.94, 153.44, 153.13, 143.43, 139.89, 139.14, 139.05, 135.39, 134.62, 131.71, 130.55, 128.72, 125.34, 106.99, 106.46, 94.57, 82.79, 61.17, 61.11, 56.49, 56.42, 52.42, 28.09. HRMS (ESI) *m/z* calcd for $C_{23}H_{25}INaO_6^+$ $[M + Na]^+$ 547.0594, found 547.0602.

Ethyl (2*Z*)-3-(1*H*-pyrrol-2-yl)-2-[(*Z*)-3,4,5-trimethoxybenzoyl]prop-2-enoate (**9ag**)

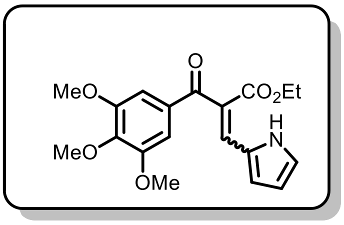

Purified by column chromatography (hexane/EtOAc 95:5–8:2) to give **9ag** (316 mg, 0,88 mmol, 88%) as a brown solid. *E*/*Z* ≈ 1:10. mp 105-108 ºC. ^{1}H NMR (500 MHz, $CDCl_3$) δ 11.90 (s, 1H), 7.34 (s, 1H), 7.28 (s, 1H), 7.09 (d, *J* = 7.6 Hz, 2H), 6.80 – 6.74 (m, 1H), 6.39 (d, *J* = 3.7 Hz, 1H), 4.17 (q, *J* = 7.1 Hz, 2H), 3.94 (s, 3H), 3.90 (s, 6H), 1.07 (t, *J* = 7.1 Hz, 3H). ^{13}C NMR (126 MHz, $CDCl_3$) δ193.92, 167.5, 152.98, 141.98, 136.92, 134.09, 127.54, 125.95, 123.30, 120.42, 111.58, 106.30, 61.20, 61.01, 56.33, 13.76. HRMS (ESI) *m/z* calcd for $C_{19}H_{22}NO_6^+$ $[M + H]^+$ 360.1442, found 360.1449.

Tert-butyl (2*Z*)-3-(1,3-thiazol-2-yl)-2-[(*Z*)-3,4,5-trimethoxybenzoyl]prop-2-enoate (**9ah**)

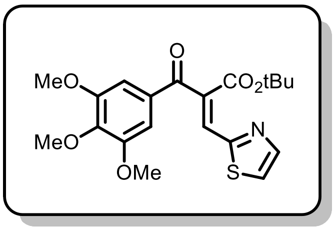

Purified by column chromatography (hexane/EtOAc 95:5–8:2) to give **9ah** (310 mg, 0,77 mmol, 77%) as a brown oil. ^{1}H NMR (400 MHz, $CDCl_3$) δ 7.94 (s, 1H), 7.83 (d, *J* = 3.2 Hz, 1H), 7.40 (d, *J* = 3.1 Hz, 1H), 7.20 (s, 2H), 3.91 (s, 3H), 3.85 (s, 6H), 1.41 (s, 9H). ^{13}C NMR (101 MHz, $CDCl_3$) δ 193.11, 163.44, 160.37, 153.32, 145.08, 143.29, 135.64, 132.00, 131.47, 123.13, 106.32, 61.06, 56.37, 27.99. HRMS (ESI) *m/z* calcd for $C_{20}H_{24}NO_6S^+$ $[M + H]^+$ 406.1319, found 406.1326.

Ethyl (2*Z*)-2-[(*Z*)-3,4,5-trimethoxybenzoyl]hex-2-enoate (**9ai**)

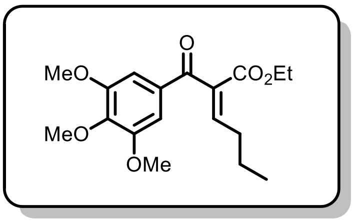

Purified by column chromatography (hexane/EtOAc 95:5–9:1) to give **9ai** (210 mg, 0,63 mmol, 63%) as a yellow oil. ^{1}H NMR (400 MHz, $CDCl_3$) δ 7.28 (s, 1H), 7.18 (s, 2H), 4.25 – 4.18 (m, 2H), 3.96 (s, 3H), 3.91 (s, 6H), 2.12 (dd, *J* = 15.0, 7.6 Hz, 2H), 1.51 (dq, *J* = 14.6, 7.4 Hz, 2H), 1.21 (t, *J* = 7.1 Hz, 3H), 0.92 (t, *J* = 7.4 Hz, 3H). ^{13}C NMR (101 MHz, $CDCl_3$) δ 193.33, 164.62, 153.21, 148.04, 143.19, 133.65, 131.93, 106.49, 61.20, 60.97, 56.29, 31.59, 29.70, 21.79, 14.11, 13.81. HRMS (ESI) *m/z* calcd for $C_{18}H_{25}O_6^+$ $[M + H]^+$ 337,1646, found 337,1640.

Ethyl (2*Z*)-2-[(*Z*)-3,5-dimethoxybenzoyl]-3-(4-methylphenyl)prop-2-enoate (**9bj**)

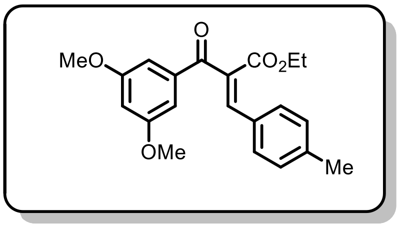

Purified by column chromatography (hexane/EtOAc 95:5–9:1) to give **9bj** (322 mg, 0,91 mmol, 91%) as a yellow solid. mp 80-83 °C ^{1}H NMR (400 MHz, $CDCl_3$) δ 7.90 (s, 1H), 7.60 (d, *J* = 1.9 Hz, 1H), 7.48 (dd, *J* = 8.4, 1.9 Hz, 1H), 7.25 (d, *J* = 8.2 Hz, 2H), 7.03 (d, *J* = 8.1 Hz, 2H), 6.79 (d, *J* = 8.4 Hz, 1H), 4.22 (q, *J* = 7.1 Hz, 2H), 3.91 (s, 3H), 3.89 (s, 3H), 2.26 (s, 3H), 1.20 (t, *J* = 7.1 Hz, 3H). ^{13}C NMR (101 MHz, $CDCl_3$) δ 194.52, 165.43, 154.11, 149.41, 142.29, 140.93, 130.38, 130.32, 130.29, 129.63, 129.59, 125.00, 110.35, 110.30, 61.51, 56.13, 56.07, 21.48, 14.22. HRMS (ESI) *m/z* calcd for $C_{21}H_{23}O_5^+$ $[M + H]^+$ 355.1540, found 355.1541.

Ethyl (2*Z*)-2-[(*Z*)-3,5-dimethoxybenzoyl]-3-(naphthalen-2-yl)prop-2-enoate (**9bk**)

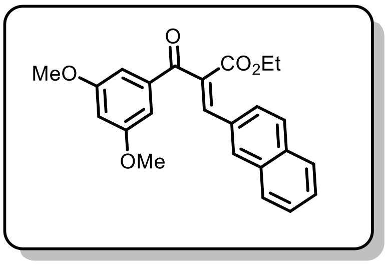

Purified by column chromatography (hexane/EtOAc 95:5–8:2) to give **9bk** (312 mg, 0,80 mmol, 80%) as a yellow oil. ^{1}H NMR (500 MHz, $CDCl_3$) δ 8.13 (s, 1H), 7.92 (s, 1H), 7.81 – 7.72 (m, 2H), 7.68 (d, *J* = 8.7 Hz, 1H), 7.52 – 7.44 (m, 2H), 7.41 (dd, *J* = 8.6, 1.6 Hz, 1H), 7.18 (d, *J* = 2.3 Hz, 2H), 6.65 (t, *J* = 2.3 Hz, 1H), 4.29 (q, *J* = 7.1 Hz, 2H), 3.79 (s, 6H), 1.25 (t, *J* = 7.1 Hz, 3H). ^{13}C NMR (126 MHz, $CDCl_3$) δ 195.57, 142.74, 138.35, 134.02, 133.12, 131.95, 131.36, 130.58, 128.87, 128.72, 127.76, 126.80, 126.02, 106.98, 106.53, 61.74, 55.69, 14.27. HRMS (ESI) *m/z* calcd for $C_{24}H_{23}O_5^+$ $[M + H]^+$ 391.1540, found 391.1547.

Ethyl (2*Z*)-2-[(*Z*)-3,5-dimethoxybenzoyl]-3-(3,4,5-trimethoxyphenyl)prop-2-enoate (**9bc**)

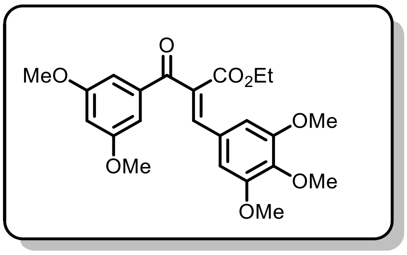

Purified by column chromatography (hexane/EtOAc 95:5–9:1) to give **9bc** (383 mg, 0,89 mmol, 89%) as a yellow solid. mp 126-128 °C. ^{1}H NMR (400 MHz, $CDCl_3$) δ 7.83 (s, 1H), 7.11 (d, *J* = 2.3 Hz, 2H), 6.64 (s, 1H), 6.57 (s, 2H), 4.23 (q, *J* = 7.1 Hz, 2H), 3.80 (s, 3H), 3.78 (s, 6H), 3.65 (s, 6H), 1.21 (t, *J* = 7.1 Hz, 3H). ^{13}C NMR (101 MHz, $CDCl_3$) δ 195.54, 165.03, 161.25, 153.13, 142.53, 140.08, 138.28, 130.46, 128.22, 107.82, 106.85, 106.39, 61.65, 60.94, 55.99, 55.70, 14.21. HRMS (ESI) *m/z* calcd for $C_{23}H_{27}O_8^+$ [M + H]$^+$ 431.1700, found 431.1707.

(*E*/*Z*)-Ethyl -2-[3,5-dimethoxybenzoyl]-3-(1*H*-pyrrol-2-yl)prop-2-enoate (**9bg**)

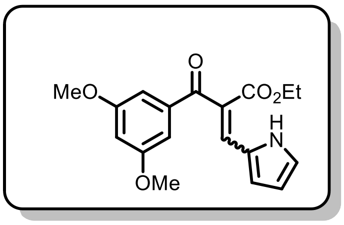

Purified by column chromatography (hexane/EtOAc 95:5–8:2) to give **9bg** (389 mg, 0,88 mmol, 88%) as a yellow oil. *E*/*Z* ≈ 1:1. ^{1}H NMR (500 MHz, $CDCl_3$) δ 11.84 (s, 1H), 10.39 (s, 1H), 9.47 (d, *J* = 1.2 Hz, 0H), 7.13 – 7.02 (m, 1H), 7.00 – 6.92 (m, 1H), 6.90 (dd, *J* = 3.9, 2.3 Hz, 2H), 6.70 (dq, *J* = 3.8, 1.7 Hz, 1H), 6.59 (td, *J* = 2.3, 1.1 Hz, 1H), 6.38 – 6.24 (m, 1H), 4.19 – 3.99 (m, 2H), 3.76 (d, *J* = 6.2 Hz, 6H), 0.98 (dt, *J* = 14.4, 7.2 Hz, 3H). ^{13}C NMR (126 MHz, $CDCl_3$) δ 196.83, 179.45, 167.52, 160.81, 160.77, 141.42, 141.19, 137.31, 136.33, 127.70, 127.65, 126.71, 126.25, 125.92, 123.65, 122.52, 120.93, 111.91, 111.70, 111.39, 106.53, 106.11, 105.58, 104.97, 61.26, 61.09, 55.70, 55.66, 14.02, 13.76. HRMS (ESI) *m/z* calcd for $C_{18}H_{20}NO_5^+$ [M + H]$^+$ 330.1336, found 330.1340.

Ethyl (2*Z*)-2-[(*Z*)-3,4-dimethoxybenzoyl]-3-(3,4,5-trimethoxyphenyl)prop-2-enoate (**9cc**) [1]

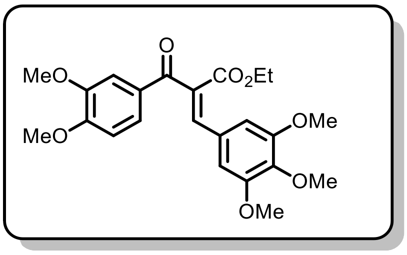

Purified by column chromatography (hexane/EtOAc 95:5–8:2) to give **9cc** (426 mg, 0,99 mmol, 99%) as a yellow oil. ^{1}H NMR (400 MHz, $CDCl_3$) δ 7.82 (s, 1H), 7.60 (d, *J* = 1.8 Hz, 1H), 7.50 (dd, *J* = 8.4, 1.8 Hz, 1H), 6.82 (d, *J* = 8.4 Hz, 1H), 6.59 (s, 2H), 4.23 (q, *J* = 7.1 Hz, 2H), 3.90 (d, *J* = 1.7 Hz, 6H), 3.78 (s, 3H), 3.64 (s, 6H), 1.20 (t, *J* = 7.1 Hz, 3H). ^{13}C NMR (101 MHz, $CDCl_3$) δ 194.43, 165.29, 154.24, 153.10, 149.52, 142.18, 139.99, 130.57, 129.68, 128.32, 125.05, 110.49, 110.03, 107.81, 61.61, 60.94, 56.20, 56.16, 55.98, 14.24. HRMS (ESI) *m/z* calcd for $C_{23}H_{27}O_8^+$ $[M + H]^+$ 431.1700, found 431.1708.

Ethyl (2*Z*)-2-[(*Z*)-2*H*-1,3-benzodioxole-5-carbonyl]-3-(3,4,5-trimethoxyphenyl)prop-2-enoate (**9dc**) [2]

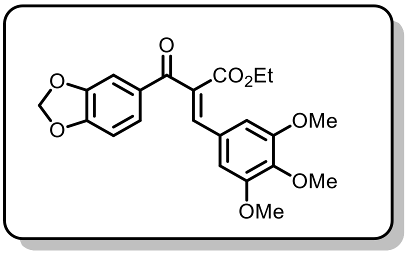

Purified by column chromatography (hexane/EtOAc 95:5–9:1) to give **9dc** (327 mg, 0,79 mmol, 79%) as a brown solid. mp 70-72 °C (lit. 97-98 °C). ^{1}H NMR (400 MHz, $CDCl_3$) δ 7.82 (s, 1H), 7.54 (dd, *J* = 8.2, 1.7 Hz, 1H), 7.48 (d, *J* = 1.6 Hz, 1H), 6.81 (d, *J* = 8.2 Hz, 1H), 6.61 (s, 2H), 6.06 (s, 2H), 4.25 (q, *J* = 7.1 Hz, 2H), 3.82 (s, 3H), 3.69 (s, 6H), 1.23 (t, *J* = 7.1 Hz, 3H). ^{13}C NMR (101 MHz, $CDCl_3$) δ 194.04, 165.23, 153.20, 152.83, 148.71, 108.48, 108.23, 107.83, 102.25, 61.70, 61.03, 56.09, 14.30. HRMS (ESI) *m/z* calcd for $C_{22}H_{23}O_8^+$ $[M + H]^+$ 415.1387, found 415.1405.

Ethyl (2*Z*)-3-(2*H*-1,3-benzodioxol-5-yl)-2-[(*Z*)-2*H*-1,3-benzodioxole-5-carbonyl]prop-2-enoate (**9dl**)

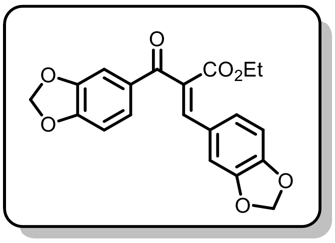

Purified by column chromatography (hexane/EtOAc 95:5–9:1) to give **9dl** (305 mg, 0,83 mmol, 83%) as a brown solid. mp 103-105 °C. ^{1}H NMR (500 MHz, $CDCl_3$) δ 7.80 (s, 1H), 7.57 – 7.47 (m, 2H), 6.94 (dd, *J* = 8.2, 1.7 Hz, 1H), 6.80 (t, *J* = 4.9 Hz, 2H), 6.72 (d, *J* = 8.1 Hz, 1H), 6.05 (s, 2H), 5.93 (s, 2H), 4.22 (q, *J* = 7.1 Hz, 2H), 1.21 (t, *J* = 7.1 Hz, 3H). ^{13}C NMR (126 MHz, $CDCl_3$) δ 194.12, 165.40, 152.80, 149.77, 148.67, 148.24, 142.04, 131.41, 129.32, 127.26, 126.90,

126.56, 109.32, 108.73, 108.41, 108.37, 102.20, 101.75, 61.59, 14.27. HRMS (ESI) *m/z* calcd for $C_{20}H_{17}O_7^+$ $[M + H]^+$ 369.0969, found 369.0972.

Ethyl (2*Z*)-2-[(*Z*)-2*H*-1,3-benzodioxole-5-carbonyl]-3-(4-bromophenyl)prop-2-enoate (**9dm**)

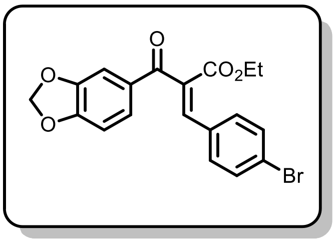

Purified by column chromatography (hexane/EtOAc 95:5–9:1) to give **9dm** (462 mg, 0,90 mmol, 90%) as a brown oil. ^{1}H NMR (400 MHz, $CDCl_3$) δ 7.83 (s, 1H), 7.50 – 7.44 (m, 2H), 7.40 – 7.35 (m, 2H), 7.26 – 7.20 (m, 2H), 6.78 (d, *J* = 8.1 Hz, 1H), 6.04 (s, 2H), 4.24 (q, *J* = 7.1 Hz, 2H), 1.21 (t, *J* = 7.1 Hz, 3H). ^{13}C NMR (101 MHz, $CDCl_3$) δ 193.41, 164.95, 152.94, 148.71, 140.77, 132.24, 132.19, 131.95, 131.61, 131.03, 126.56, 124.97, 108.37, 108.25, 102.24, 61.82, 14.21. HRMS (ESI) *m/z* calcd for $C_{19}H_{16}BrO_5^+$ $[M + H]^+$ 403.0176, found 403.0185.

Ethyl (2*Z*)-2-[(*Z*)-3-methoxybenzoyl]-3-(3,4,5-trimethoxyphenyl)prop-2-enoate (**9ec**)

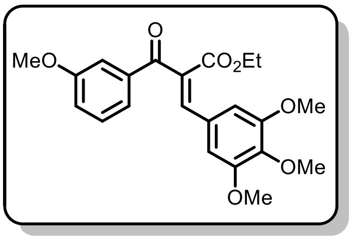

Purified by column chromatography (hexane/EtOAc 95:5–9:1) to give **9ec** (393 mg, 0,98 mmol, 98%) as a yellow oil. mp 95.5-96.6 ºC (lit. 97-98 ºC). ^{1}H NMR (400 MHz, $CDCl_3$) δ 7.83 (s, 1H), 7.50 (d, *J* = 7.4 Hz, 2H), 7.35 – 7.25 (m, 1H), 7.08 (d, *J* = 8.0 Hz, 1H), 6.56 (s, 2H), 4.21 (q, *J* = 7.0 Hz, 2H), 3.79 (s, 3H), 3,77 (s, 3H), 3.61 (s, 6H), 1.17 (t, *J* = 7.0 Hz, 3H). ^{13}C NMR (101 MHz, $CDCl_3$) δ 195.58, 164.97, 160.12, 153.06, 142.40, 140.01, 137.66, 130.53, 130.05, 128.15, 122.08, 120.51, 112.84, 107.75, 61.53, 60.83, 55.88, 55.47, 14.11. . HRMS (ESI) *m/z* calcd for $C_{22}H_{25}O_7^+$ $[M + H]^+$ 401.1595, found 401.1602.

Ethyl (2*Z*)-2-[(*Z*)-4-methoxybenzoyl]-3-(3,4,5-trimethoxyphenyl)prop-2-enoate (**9fc**)

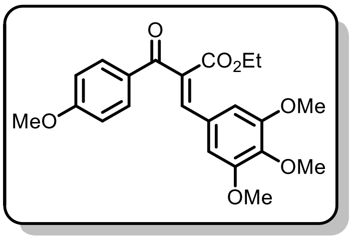

Purified by column chromatography (hexane/EtOAc 95:5–9:1) to give **9fc** (395 mg, 0,99 mmol, 99%) as a yellow oil. ^{1}H NMR (250 MHz, $CDCl_3$) δ 7.92 (d, *J* = 8.8 Hz, 2H), 7.79 (s, 1H), 6.88 (d, *J* = 8.8 Hz, 2H), 6.58 (s, 2H), 4.21 (q, *J* = 7.1 Hz, 2H), 3.82 (s, 3H), 3.77 (s, 3H), 3.62 (s, 6H), 1.18 (t, *J* = 7.1 Hz, 3H). ^{13}C NMR (63 MHz, $CDCl_3$) δ 194.36, 165.28, 164.38, 153.12,

142.01, 139.98, 131.58, 130.77, 129.61, 128.34, 114.29, 107.83, 61.57, 60.93, 55.99, 55.62, 14.22. HRMS (ESI) *m/z* calcd for $C_{22}H_{25}O_7^+$ $[M + H]^+$ 401.1595, found 401.1600.

Ethyl (2*Z*)-2-[(*Z*)-1-benzothiophene-3-carbonyl]-3-(3,4,5-trimethoxyphenyl)prop-2-enoate (**9gc**)

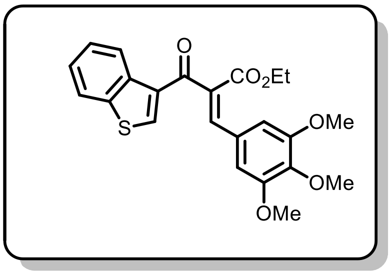

Purified by column chromatography (hexane/EtOAc 95:5–8:2) to give **9gc** (414 mg, 0,97 mmol, 97%) as a brown solid. mp 119-122 °C. ^{1}H NMR (400 MHz, $CDCl_3$) δ 8.92 (d, *J* = 8.1 Hz, 1H), 8.21 (s, 1H), 7.85 (d, *J* = 8.8 Hz, 2H), 7.54 (dd, *J* = 11.2, 3.9 Hz, 1H), 7.45 (dd, *J* = 11.2, 3.8 Hz, 1H), 6.65 (s, 2H), 4.33 – 4.23 (m, 2H), 3.77 (s, 3H), 3.54 (s, 6H), 1.23 (t, *J* = 7.1 Hz, 3H). ^{13}C NMR (101 MHz, $CDCl_3$) δ 189.95, 165.13, 153.03, 142.04, 140.89, 140.22, 139.96, 136.12, 134.88, 131.21, 128.11, 126.19, 125.89, 125.28, 122.47, 107.65, 61.61, 60.79, 55.80, 14.17. HRMS (ESI) *m/z* calcd for $C_{23}H_{23}O_6S^+$ $[M + H]^+$ 427.1210, found 427.1215.

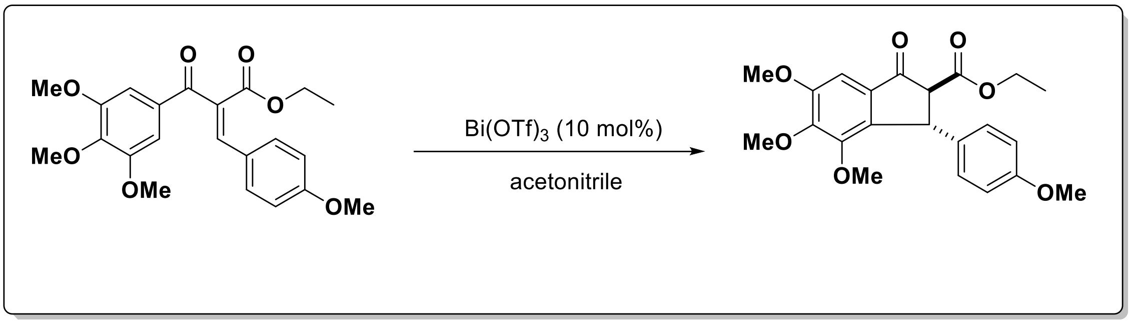

To a sealed tube were added the Knoevenagel product **9** (0.5 mmol), dry acetonitrile (2 mL), and $Bi(OTf)_3$ (0.05 mmol). The reaction mixture was stirred at 60 °C using magnetic stirring. The reaction was monitored by TLC. After confirming the completion of the reaction, the reaction mixture was concentrated under reduced pressure. The resulting crude reaction mixture was purified by column chromatography using a mobile phase of hexane/ethyl acetate 9:1–8:2, v/v.

Ethyl 4,5,6-trimethoxy-3-(4-methoxyphenyl)-1-oxo-2,3-dihydro-1*H*-indene-2-carboxylate (**10aa**)

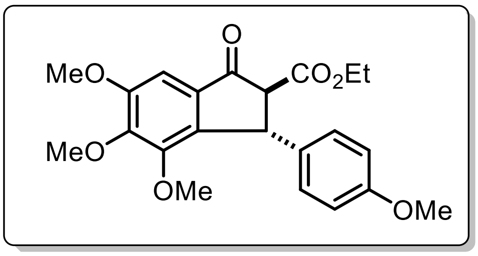

Purified by column chromatography (hexane/EtOAc 95:5–9:1) to give **10aa** (112 mg, 0,28 mmol, 93%) as a yellow oil. ^{1}H NMR (250 MHz, $CDCl_3$) δ 7.09 – 7.00 (m, 3H), 6.82 (d, *J* = 8.6 Hz, 2H), 4.89 (d, *J* = 3.1 Hz, 1H), 4.23 (q, *J* = 7.1 Hz, 2H), 3.90 (s, 6H), 3.76 (s, 3H), 3.56 (d, *J* = 3.1 Hz, 1H), 3.38 (s, 3H), 1.28 (t, *J* = 7.2 Hz, 3H). ^{13}C NMR (63 MHz, $CDCl_3$) δ 198.25, 168.65, 158.81, 155.33, 150.49, 149.62, 143.82, 134.97, 130.66, 128.57, 114.28, 101.14, 64.12, 61.94, 61.08, 60.28, 56.44, 55.41, 45.70, 14.36. HRMS (ESI) *m/z* calcd for $C_{22}H_{25}O_7^+$ [M + H]$^+$ 401.1595, found 401.1565.

Ethyl 4,5,6-trimethoxy-3-(4-chlorophenyl)-1-oxo-2,3-dihydro-1*H*-indene-2-carboxylate (**10ab**)

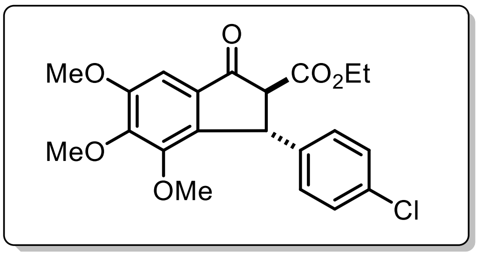

Purified by column chromatography (hexane/EtOAc 95:5–9:1) to give **10ab** (104 mg, 0,26 mmol, 86%) as a brown oil. ^{1}H NMR (400 MHz, $CDCl_3$) δ 7.29 (d, *J* = 8.2 Hz, 2H), 7.10 (s, 1H), 7.08 (d, *J* = 8.3 Hz, 2H), 4.93 (d, *J* = 3.2 Hz, 1H), 4.26 (q, *J* = 7.0 Hz, 2H), 3.93 (d, *J* = 2.3 Hz, 6H), 3.56 (d, *J* = 3.3 Hz, 1H), 3.45 (s, 3H), 1.32 (t, *J* = 7.1 Hz, 3H). ^{13}C NMR (101 MHz, $CDCl_3$) δ 197.47, 168.19, 155.42, 150.19, 149.37, 142.67, 141.31, 132.88, 130.51, 128.92, 128.76, 101.03, 63.61, 61.96, 60.96, 60.13, 56.31, 45.51, 14.20. HRMS (ESI) *m/z* calcd for $C_{21}H_{22}ClO_6^+$ [M + H]$^+$ 405.1099, found 405.1107.

Ethyl 4,5,6-trimethoxy-1-oxo-3-(3,4,5-trimethoxyphenyl)-2,3-dihydro-1*H*-indene-2-carboxylate (**10ac**)

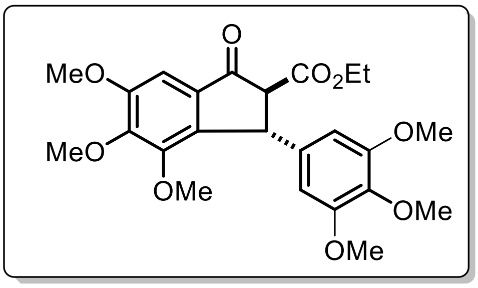

Purified by column chromatography (hexane/EtOAc 95:5–8:2) to give **10ac** (127 mg, 0,28 mmol, 93%) as a brown oil. ^{1}H NMR (500 MHz, $CDCl_3$) δ7.09 (s, 1H), 6.32 (s, 2H), 4.88 (d, *J* = 3.3 Hz, 1H), 4.26 (qd, *J* = 7.1, 2.2 Hz, 2H), 3.92 (d, *J* = 1.3 Hz, 6H), 3.82 (s, 3H), 3.78 (s, 6H), 3.62 (d, *J* = 3.2 Hz, 1H), 3.45 (s, 3H), 1.31 (t, *J* = 7.1 Hz, 3H).. ^{13}C NMR (126 MHz, $CDCl_3$) δ197.97, 168.51, 155.43, 153.59, 150.50, 149.57, 143.19, 138.61, 137.23, 130.64, 104.56, 101.18, 63.83, 62.06, 61.10, 61.04, 60.35, 56.43, 56.33, 46.57, 14.37. HRMS (ESI) *m/z* calcd for $C_{24}H_{29}O_9^+$ $[M + H]^+$ 461.1806, found 461.1817.

Ethyl 3-(3,4-dimethoxyphenyl)-4,5,6-trimethoxy-1-oxo-2,3-dihydro-1*H*-indene-2-carboxylate (**10ad**)

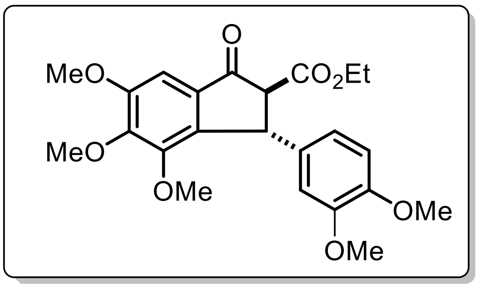

Purified by column chromatography (hexane/EtOAc 95:5–8:2) to give **10ad** (120 mg, 0,28 mmol, 94%) as a yellow oil. ^{1}H NMR (400 MHz, $CDCl_3$) δ 7.05 (s, 1H), 6.75 (d, *J* = 8.1 Hz, 1H), 6.64 – 6.59 (m, 2H), 4.86 (d, *J* = 3.1 Hz, 1H), 4.21 (q, *J* = 6.9 Hz, 2H), 3.88 (s, 6H), 3.82 (s, 3H), 3.78 (s, 3H), 3.57 (d, *J* = 3.1 Hz, 1H), 3.38 (s, 3H), 1.27 (t, *J* = 7.1 Hz, 3H). ^{13}C NMR (101 MHz, $CDCl_3$) δ 198.21, 168.63, 155.37, 150.52, 149.63, 149.31, 148.27, 143.61, 135.48, 130.65, 119.62, 111.51, 110.83, 101.17, 64.08, 62.02, 61.11, 60.36, 56.45, 56.13, 56.07, 46.06, 14.39. HRMS (ESI) *m/z* calcd for $C_{23}H_{27}O_8^+$ $[M + H]^+$ 431.1700, found 431.1708.

Ethyl 4,5,6-trimethoxy-1-oxo-3-(1*H*-pyrrol-2-yl)-2,3-dihydro-1*H*-indene-2-carboxylate (**10ag**)

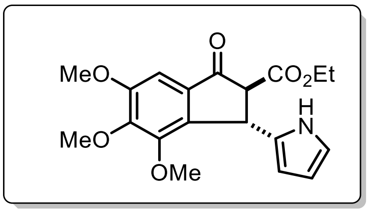

Purified by column chromatography (hexane/EtOAc 95:5–8:2) to give **10ag** (87 mg, 0,24 mmol, 81%) as a yellow oil. ^{1}H NMR (400 MHz, $CDCl_3$) δ 8.83 (s, 1H), 7.28 (s, 1H), 7.07 (s, 1H), 6.73 (d, *J* = 1.4 Hz, 1H), 6.13 (d, *J* = 3.0 Hz, 1H), 5.96 (s, 1H), 5.06 (d, *J* = 2.9 Hz, 1H), 4.27 (q, *J* = 7.1 Hz, 2H), 3.98 (s, 3H), 3.93 – 3.89 (m, 1H), 3.91 (s, 3H), 3.76 (s, 3H), 1.33 (t, *J* = 7.1 Hz, 3H). ^{13}C NMR (101 MHz, $CDCl_3$) δ 197.23, 168.41, 155.13, 149.90, 149.24, 141.85, 131.50, 129.95, 117.45, 108.43, 105.18, 101.78, 62.01, 61.26, 61.07, 60.94, 56.32, 39.14, 14.19. HRMS (ESI) *m/z* calcd for $C_{19}H_{22}NO_6^+$ $[M + H]^+$ 360.1442, found 360.1433.

Ethyl 4,6-dimethoxy-3-(4-methylphenyl)-1-oxo-2,3-dihydro-1*H*-indene-2-carboxylate (**10bj**)

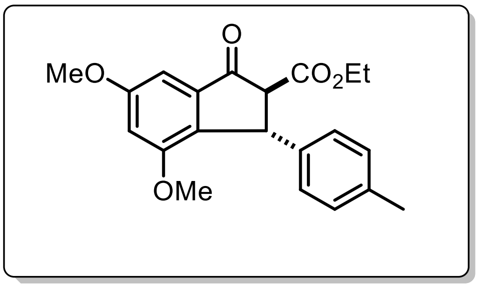

Purified by column chromatography (hexane/EtOAc 95:5–9:1) to give **10bj** (88 mg, 0,25 mmol, 83%) as a yellow oil. ^{1}H NMR (500 MHz, $CDCl_3$) δ 7.23 (s, 1H), 7.18 (d, *J* = 3.7 Hz, 1H), 7.10 (d, *J* = 8.0 Hz, 2H), 6.99 (dd, *J* = 11.5, 8.0 Hz, 2H), 6.61 (d, *J* = 2.5 Hz, 1H), 4.82 (d, *J* = 4.0 Hz, 1H), 4.22 (dd, *J* = 7.1, 5.2 Hz, 2H), 3.90 (s, 3H), 3.81 (s, 3H), 3.56 (d, *J* = 4.1 Hz, 1H), 2.31 (s, 3H), 1.27 (t, *J* = 7.1 Hz, 3H). ^{13}C NMR (126 MHz, $CDCl_3$) δ 197.46, 168.99, 156.49, 152.24, 150.29, 139.20, 137.32, 129.90, 129.76, 128.20, 127.91, 127.61, 107.46, 104.42, 64.11, 61.91, 56.56, 56.38, 48.25, 21.26, 14.42. HRMS (ESI) *m/z* calcd for $C_{21}H_{23}O_5^+$ $[M + H]^+$ 355.1540, found 355.1545.

Ethyl 4,6-dimethoxy-3-(naphthalen-2-yl)-1-oxo-2,3-dihydro-1*H*-indene-2-carboxylate (**10bk**)

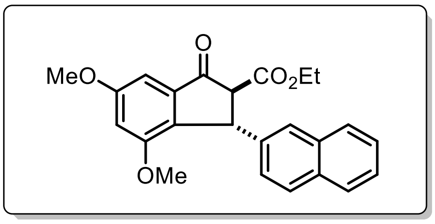

Purified by column chromatography (hexane/EtOAc 95:5–8:2) to give **10bk** (94 mg, 0,24 mmol, 80%) as an orange solid. mp 130-131 °C. ^{1}H NMR (500 MHz, $CDCl_3$) δ 7.91 – 7.72 (m, 3H), 7.58 (d, *J* = 1.8 Hz, 1H), 7.54 – 7.41 (m, 3H), 7.18 (dd, *J* = 8.5, 1.9 Hz, 1H), 6.93 (d, *J* = 2.1 Hz, 1H), 6.70 (d, *J* = 2.2 Hz, 1H), 5.13 (d, *J* = 3.1 Hz, 1H), 4.29 (q, *J* = 7.1 Hz, 2H), 3.90 (s, 3H), 3,70 (d, *J* = 3,0 Hz, 2H), 3.61 (s, 3H), 1.33 (t, *J* = 7.1 Hz, 3H). ^{13}C NMR (126 MHz, $CDCl_3$) δ 199.05, 168.37, 162.14, 157.88, 139.80, 138.13, 137.76, 133.47, 132.49, 128.49, 127.75, 127.66, 126.17, 125.88, 125.70, 125.31, 106.92, 96.61, 64.19, 61.90, 55.86, 55.62, 46.09, 14.23. HRMS (ESI) *m/z* calcd for $C_{24}H_{23}O_5^+$ [M + H]$^+$ 391.1540, found 391.1544.

Ethyl 4,6-dimethoxy-1-oxo-3-(3,4,5-trimethoxyphenyl)-2,3-dihydro-1*H*-indene-2-carboxylate (**10bc**)

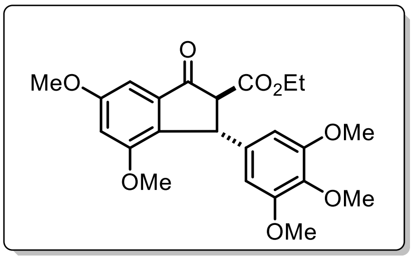

Purified by column chromatography (hexane/EtOAc 95:5–9:1) to give **10bc** (110 mg, 0,26 mmol, 85%) as a yellow solid. mp 144-145 °C. ^{1}H NMR (400 MHz, $CDCl_3$) δ 6.87 (d, *J* = 2.1 Hz, 1H), 6.69 (d, *J* = 2.1 Hz, 1H), 6.27 (s, 2H), 4.86 (d, *J* = 2.9 Hz, 2H), 4.31 – 4.23 (q, *J* = 7.1 Hz, 2H), 3.88 (s, 3H), 3.85 – 3.82 (m, 3H), 3.77 (s, 6H), 3.70 (s, 3H), 3.62 (d, *J* = 3.0 Hz, 1H), 1.32 (t, *J* = 7.1 Hz, 3H). ^{13}C NMR (101 MHz, $CDCl_3$) δ199.11, 168.49, 162.24, 158.01, 153.46, 138.28, 138.05, 137.77, 137.01, 107.05, 104.34, 96.76, 64.29, 62.04, 61.00, 56.29, 55.98, 55.85, 46.29, 14.40. HRMS (ESI) *m/z* calcd for $C_{23}H_{27}O_8^+$ [M + H]$^+$ 431.1700, found 431.1708.

Ethyl 4,6-dimethoxy-1-oxo-3-(1*H*-pyrrol-2-yl)-2,3-dihydro-1*H*-indene-2-carboxylate (**10bg**)

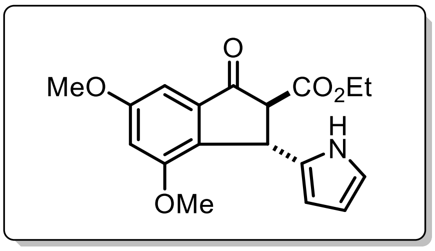

Purified by column chromatography (hexane/EtOAc 95:5–8:2) to give **10bg** (76 mg, 0,23 mmol, 77%) as a yellow solid. mp 100-102 °C. ^{1}H NMR (500 MHz, $CDCl_3$) δ ^{1}H NMR (500 MHz, $CDCl_3$) δ 8.57 (s, 1H), 6.72 (d, *J* = 2.1 Hz, 1H), 6.62 (d, *J* = 2.1 Hz, 1H), 6.60 (m, 1H), 5.99 (q, *J* = 2.9 Hz, 1H), 5.79 (m, 1H), 4.91 (d, *J* = 2.7 Hz, 1H), 4.14 (q, *J* = 7.1 Hz, 2H), 3.84 (s, 0H), 3.77 (s, 3H), 3.73 (s, 3H), 1.21 (t, *J* = 7.2 Hz, 3H). . ^{13}C NMR (125 MHz, $CDCl_3$) δ ^{13}C NMR (126 MHz, $CDCl_3$) δ 198.52, 168.50, 162.11, 157.50, 137.26, 137.22, 131.77, 117.51, 108.44, 107.29, 104.96, 97.58, 62.21, 61.62, 56.24, 56.06, 39.14, 14.40. . HRMS (ESI) *m/z* calcd for $C_{18}H_{19}NNaO_5^+$ [M + Na]$^+$ 352.1155, found 352.1165.

Ethyl 5,6-dimethoxy-1-oxo-3-(3,4,5-trimethoxyphenyl)-2,3-dihydro-1*H*-indene-2-carboxylate (**10cc**)

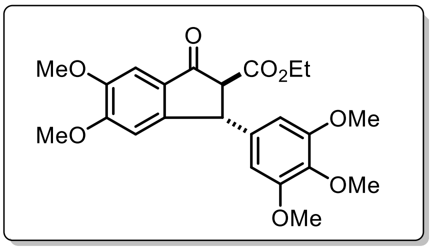

Purified by column chromatography (hexane/EtOAc 95:5–8:2) to give **10cc** (104 mg, 0,24 mmol, 81%) as a brown oil. ^{1}H NMR (400 MHz, $CDCl_3$) δ 7.23 (s, 1H), 6.69 (s, 1H), 6.34 (s, 2H), 4.84 (d, *J* = 4.1 Hz, 1H), 4.29 (dd, *J* = 7.1, 4.3 Hz, 2H), 3.95 (s, 3H), 3.90 (s, 3H), 3.85 (s, 3H), 3.80 (s, 6H), 3.63 (d, *J* = 4.1 Hz, 1H), 1.33 (t, *J* = 7.1 Hz, 3H). ^{13}C NMR (101 MHz, $CDCl_3$) δ197.15, 168.91, 156.57, 153.84, 151.69, 150.44, 137.85, 137.46, 128.17, 107.47, 104.94, 104.48, 63.95, 61.99, 61.01, 56.65, 56.35, 48.82, 14.43. HRMS (ESI) *m/z* calcd for $C_{23}H_{27}O_8^+$ [M + H]$^+$ 431.1700, found 431.1706.

Ethyl 5-(4-bromophenyl)-7-oxo-2*H*,5*H*,6*H*,7*H*-indeno[5,6-d][1,3]dioxole-6-carboxylate (**10dm**)

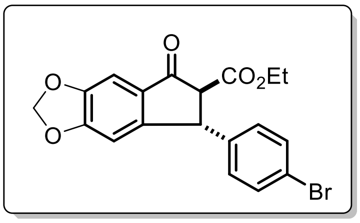

Purified by column chromatography (hexane/EtOAc 95:5–9:1) to give **10dm** (99 mg, 0,25 mmol, 82%) as a yellow oil. ^{1}H NMR (500 MHz, Chloroform-*d*) δ 7.84 (s, 1H), 7.50 – 7.45 (m, 2H), 7.39 (d, *J* = 8.6 Hz, 2H), 7.26 – 7.19 (m, 2H), 6.79 (d, *J* = 8.1 Hz, 1H), 6.05 (s, 2H), 4.25 (q, *J* = 7.1 Hz, 2H), 1.22 (t, *J* = 7.1 Hz, 3H). ^{13}C NMR (126 MHz, $CDCl_3$) δ 193.39, 164.91, 152.90, 148.66, 140.74, 132.30, 132.15, 131.89, 131.57, 130.97, 129.66, 126.53, 124.94, 108.34, 108.21, 102.20, 61.79, 14.18. HRMS (ESI) *m/z* calcd for $C_{19}H_{16}BrO_5^+$ [M + H]$^+$ 403.0176, found 403.0183.

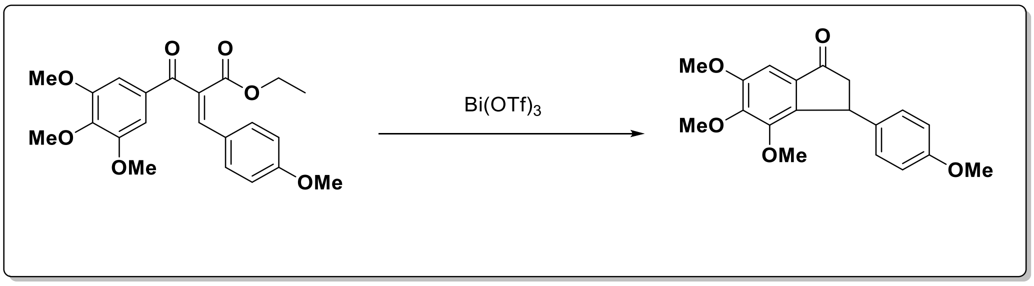

To a sealed tube were added the Knoevenagel product **9** (0.5 mmol), dry acetonitrile (2 mL), and $Bi(OTf)_3$ (0.05 mmol). The reaction mixture was stirred at 100 °C using magnetic stirring. The reaction was monitored by TLC. After confirming the completion of the reaction, the reaction mixture was concentrated under reduced pressure. The resulting crude reaction mixture was purified by column chromatography using a mobile phase of hexane/ethyl acetate 9:1–8:2, v/v.

4,5,6-Trimethoxy-3-(4-methoxyphenyl)-2,3-dihydro-1*H*-inden-1-one (**11aa**) [3]

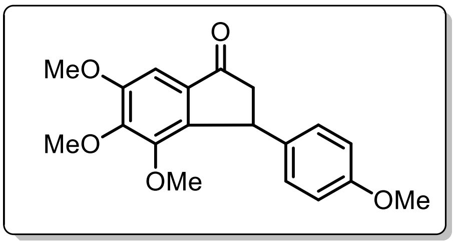

Purified by column chromatography (hexane/EtOAc 95:5–9:1) to give **11aa** (91 mg, 0,28 mmol, 93%) as a yellow solid. mp 60-62 °C. ^{1}H NMR (400 MHz, $CDCl_3$) δ 7.10 (s, 1H), 7.06 – 7.02 (m, 2H), 6.86 – 6.80 (m, 2H), 4.56 (dd, *J* = 8.0, 2.5 Hz, 1H), 3.93 (s, 3H), 3.92 (s, 3H), 3.79 (s, 3H), 3.38 (s, 3H), 3.19 (dd, *J* = 19.3,

8.0 Hz, 1H). ^{13}C NMR (101 MHz, $CDCl_3$) δ 205.69, 158.47, 155.04, 150.59, 149.01, 145.05, 136.61, 132.33, 128.40, 114.16, 100.45, 61.06, 60.28, 56.42, 55.43, 47.52, 41.03. HRMS (ESI) *m/z* calcd for $C_{19}H_{21}O_5^+$ $[M + H]^+$ 329.1384, found 190.1391.

4,5,6-Trimethoxy-3-(4-chlorophenyl)-2,3-dihydro-1*H*-inden-1-one (**11ab**) [4]

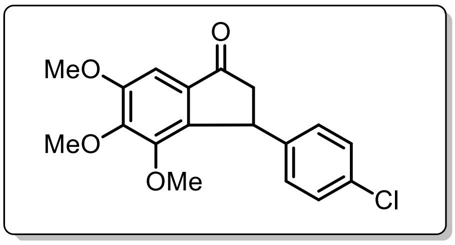

Purified by column chromatography (hexane/EtOAc 95:5–9:1) to give **11ab** (87 mg, 0,26 mmol, 87%) as a yellow oil. ^1H NMR (400 MHz, $CDCl_3$) δ 7.32 – 7.22 (m, 2H), 7.09 (s, 1H), 7.05 (d, *J* = 8.4 Hz, 2H), 4.56 (dd, *J* = 8.0, 2.5 Hz, 1H), 3.93 (s, 3H), 3.91 (s, 3H), 3.42 (s, 3H), 3.19 (dd, *J* = 19.2, 8.0 Hz, 1H), 2.56 (dd, *J* = 19.2, 2.6 Hz, 1H). ^{13}C NMR (101 MHz, $CDCl_3$) δ 204.94, 155.29, 150.45, 148.89, 144.09, 143.12, 132.49, 132.35, 128.91, 128.78, 100.49, 61.06, 60.25, 56.42, 47.16, 41.14. HRMS (ESI) *m/z* calcd for $C_{18}H_{18}ClO_4^+$ $[M + H]^+$ 333.0888, found 333.0894.

4,5,6-Trimethoxy-3-(3,4,5-trimethoxyphenyl)-2,3-dihydro-1*H*-inden-1-one (**11ac**) [4]

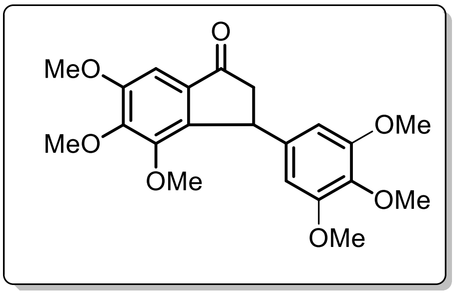

Purified by column chromatography (hexane/EtOAc 95:5–8:2) to give **11ac** (105 mg, 0,27 mmol, 90%) as a yellow oil ^1H NMR (400 MHz, $CDCl_3$) δ 7.06 (s, 1H), 6.28 (s, 2H), 4.49 (dd, *J* = 8.0, 2.5 Hz, 1H), 3.90 (s, 3H), 3.88 (s, 3H), 3.78 (s, 3H), 3.75 (s, 6H), 3.40 (s, 3H), 3.14 (dd, *J* = 19.3, 8.0 Hz, 1H), 2.59 (dd, *J* = 19.3, 2.5 Hz, 1H). ^{13}C NMR (101 MHz, $CDCl_3$) δ 205.34, 155.11, 153.47, 150.56, 148.91, 144.35, 140.27, 136.85, 132.34, 104.38, 100.46, 61.02, 60.30, 56.36, 56.28, 47.23, 42.07. HRMS (ESI) *m/z* calcd for $C_{21}H_{25}O_7^+$ $[M + H]^+$ 389.1595, found 389.1596.

4,5,6-Trimethoxy-3-(3,4-dimethoxyphenyl)-2,3-dihydro-1*H*-inden-1-one (**11ad**)

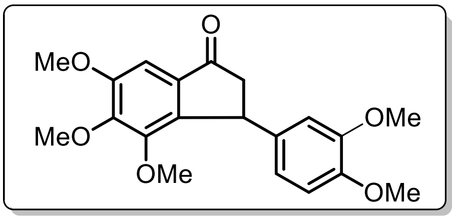

Purified by column chromatography (hexane/EtOAc 95:5–9:1) to give **11ad** (87 mg, 0,24 mmol, 81%) as a yellow oil. ^{1}H NMR (500 MHz, $CDCl_3$) δ 7.10 (s, 1H), 6.79 (d, *J* = 8.2 Hz, 1H), 6.68 – 6.57 (m, 2H), 4.55 (dd, *J* = 7.9, 2.5 Hz, 1H), 3.94 (s, 3H), 3.92 (s, 3H), 3.86 (s, 3H), 3.82 (s, 3H), 3.40 (s, 3H), 3.19 (dd, *J* = 19.2, 8.0 Hz, 1H), 2.61 (dd, *J* = 19.3, 2.5 Hz, 1H). ^{13}C NMR (126 MHz, $CDCl_3$) δ 205.47, 154.89, 150.43, 149.04, 148.83, 147.73, 144.65, 136.96, 132.17, 119.25, 111.25, 110.50, 100.30, 60.89, 60.17, 56.24, 55.93, 55.89, 47.30, 41.29. HRMS (ESI) *m/z* calcd for $C_{20}H_{23}O_6^+$ $[M + H]^+$ 359.1489, found 359.1493.

4,6-Dimethoxy-3-(4-methylphenyl)-2,3-dihydro-1*H*-inden-1-one (**11bj**) [5]

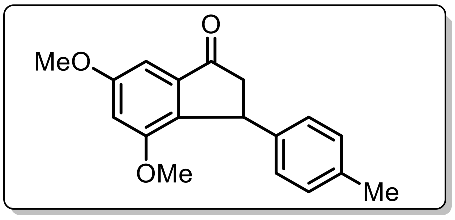

Purified by column chromatography (hexane/EtOAc 95:5–9:1) to give **11bj** (60 mg, 0,21 mmol, 71%) as a brown oil ^{1}H NMR (400 MHz, $CDCl_3$) δ 7.24 (s, 1H), 7.14 (d, *J* = 7.9 Hz, 2H), 7.03 (d, *J* = 8.0 Hz, 2H), 6.66 (s, 1H), 4.47 (dd, *J* = 7.7, 3.3 Hz, 1H), 3.95 (s, 3H), 3.86 (s, 3H), 3.21 (dd, *J* = 19.0, 7.7 Hz, 1H), 2.62 (dd, *J* = 19.0, 3.4 Hz, 1H), 2.35 (s, 3H). ^{13}C NMR (101 MHz, $CDCl_3$) δ 204.91, 155.96, 153.52, 150.01, 141.03, 136.77, 129.78, 127.62, 107.65, 103.86, 56.46, 56.34, 47.52, 44.04, 21.22. HRMS (ESI) *m/z* calcd for $C_{18}H_{19}O_3^+$ $[M + H]^+$ 283.1329, found 283.1337.

4,6-Dimethoxy-3-(naphthalen-2-yl)-2,3-dihydro-1*H*-inden-1-one (**11bk**)

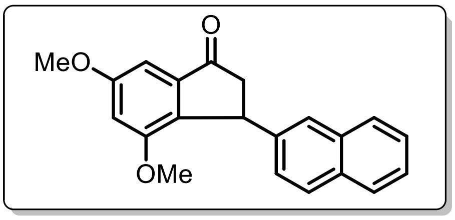

Purified by column chromatography (hexane/EtOAc 95:5–8:2) to give **11bk** (72 mg, 0,23 mmol, 76%) as a yellow solid. mp 138-139 °C. ^{1}H NMR (400 MHz, $CDCl_3$) δ 7.90 – 7.70 (m, 3H), 7.55 (d, *J* = 1.8 Hz, 1H), 7.52 – 7.36 (m, 2H), 7.18 (dd, *J* = 8.4, 1.9 Hz, 1H), 6.93 (d, *J* = 2.1 Hz, 1H), 6.67 (d, *J* = 2.1 Hz, 1H), 4.76 (dd, *J* = 8.0, 2.3 Hz, 1H), 3.91 (s, 3H), 3.62 (s, 3H), 3.30 (dd, *J* = 19.3, 7.9 Hz, 1H), 2.70 (dd, *J* = 19.3, 2.3 Hz, 1H). ^{13}C NMR (101 MHz, $CDCl_3$) δ206.50, 162.02, 158.15, 141.56,

139.54, 139.44, 133.69, 132.48, 128.44, 127.84, 127.81, 126.21, 125.71, 125.66, 125.63, 106.33, 96.12, 55.99, 55.76, 47.77, 41.63. HRMS (ESI) *m/z* calcd for $C_{21}H_{19}O_3^+$ [M + H]$^+$ 319.1329, found 319.1335.

4,6-Dimethoxy-3-(3,4,5-trimethoxyphenyl)-2,3-dihydro-1*H*-inden-1-one **(11bc**)

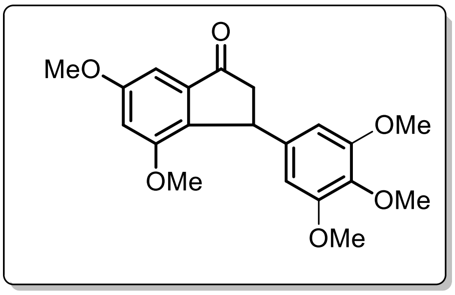

Purified by column chromatography (hexane/EtOAc 95:5–8:2) to give **11bc** (92 mg, 0,26 mmol, 86%) as a yellow solid. mp 142-143 °C. ^{1}H NMR (400 MHz, $CDCl_3$) δ 6.87 (d, *J* = 1.9 Hz, 1H), 6.67 (d, *J* = 1.9 Hz, 1H), 6.27 (s, 2H), 4.52 (dd, *J* = 7.9, 2.0 Hz, 1H), 3.88 (s, 3H), 3.83 (s, 3H), 3.78 (s, 6H), 3.71 (s, 3H), 3.21 (dd, *J* = 19.2, 7.9 Hz, 1H), 2.63 (dd, *J* = 19.2, 2.1 Hz, 1H). ^{13}C NMR (101 MHz, $CDCl_3$) δ 206.43, 161.97, 158.11, 153.36, 139.89, 139.30, 139.26, 136.69, 106.31, 104.27, 96.12, 61.01, 56.28, 55.96, 55.82, 47.73, 41.76. HRMS (ESI) *m/z* calcd for $C_{20}H_{23}O_6^+$ [M + H]$^+$ 359.1489, found 359.1492.

7-(3,4,5-Trimethoxyphenyl)-2*H*,5*H*,6*H*,7*H*-indeno[5,6-d][1,3]dioxol-5-one (**11dc**)

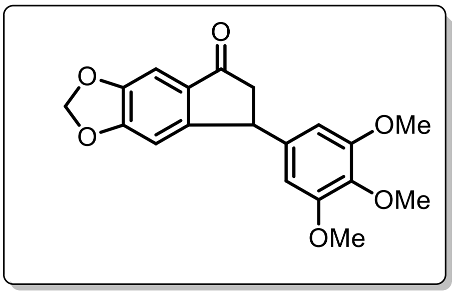

Purified by column chromatography (hexane/EtOAc 95:5–9:1) to give **11dc** (85 mg, 0,25 mmol, 83%) as a yellow solid. mp 176-178 °C. ^{1}H NMR (500 MHz, $CDCl_3$) δ 7.15 (s, 1H), 6.66 (s, 1H), 6.32 (s, 2H), 6.09 (d, *J* = 1.1 Hz, 2H), 4.37 (dd, *J* = 7.7, 3.4 Hz, 1H), 3.84 (s, 3H), 3.82 (s, 6H), 3.21 (dd, *J* = 19.0, 7.8 Hz, 1H), 2.68 (dd, *J* = 19.1, 3.5 Hz, 1H). ^{13}C NMR (126 MHz, $CDCl_3$) δ203.74, 155.35, 154.56, 153.58, 148.80, 139.22, 136.99, 131.50, 105.81, 104.47, 102.40, 101.78, 60.86, 56.19, 47.14, 44.66. HRMS (ESI) *m/z* calcd for $C_{19}H_{19}O_6^+$ [M + H]$^+$ 343.1176, found 343.1183.

7-(2*H*-1,3-Benzodioxol-5-yl)-2*H*,5*H*,6*H*,7*H*-indeno[5,6-d][1,3]dioxol-5-one (**11dl**)

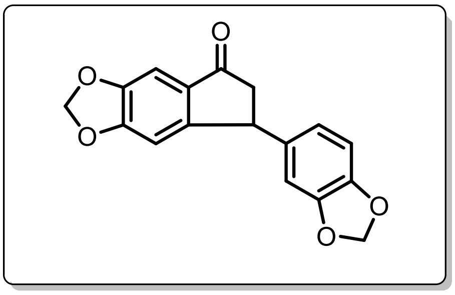

Purified by column chromatography (hexane/EtOAc 95:5–9:1) to give **11dl** (76 mg, 0,26 mmol, 86%) as a brown solid. mp 193-195 ºC. ^{1}H NMR (500 MHz, $CDCl_3$) δ 7.14 (s, 1H), 6.76 (d, *J* = 7.9 Hz, 1H), 6.69 – 6.61 (m, 2H), 6.53 (d, *J* = 1.8 Hz, 1H), 6.10 – 6.04 (m, 2H), 5.95 (d, *J* = 1.7 Hz, 1H), 4.38 (dd, *J* = 7.7, 3.4 Hz, 1H), 3.19 (dd, *J* = 19.1, 7.8 Hz, 1H), 2.62 (dd, *J* = 19.0, 3.5 Hz, 1H). ^{13}C NMR (126 MHz, $CDCl_3$) δ203.70, 155.54, 154.52, 148.73, 148.19, 146.60, 137.46, 131.51, 120.82, 108.38, 107.58, 105.81, 102.35, 101.75, 101.11, 47.30, 44.01. HRMS (ESI) *m/z* calcd for $C_{17}H_{13}O_5^+$ $[M + H]^+$ 297.0757, found 297.0762.

7-(4-Bromophenyl)-2*H*,5*H*,6*H*,7*H*-indeno[5,6-d][1,3]dioxol-5-one (**11dm**)

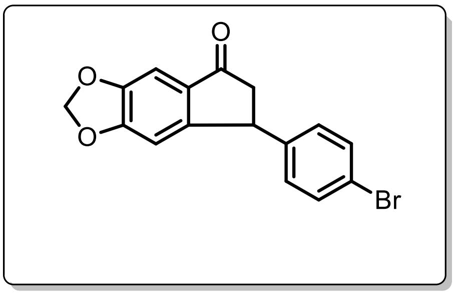

Purified by column chromatography (hexane/EtOAc 95:5–9:1) to give **11dm** (80 mg, 0,24 mmol, 81%) as a brown solid. mp 167-169 ºC. ^{1}H NMR (500 MHz, $CDCl_3$) δ 7.45 (d, *J* = 8.4 Hz, 2H), 7.15 (s, 1H), 7.01 (d, *J* = 8.4 Hz, 2H), 6.58 (s, 1H), 6.08 (d, *J* = 3.0 Hz, 2H), 4.42 (dd, *J* = 7.8, 3.3 Hz, 1H), 3.22 (dd, *J* = 19.0, 7.8 Hz, 1H), 2.61 (dd, *J* = 19.0, 3.4 Hz, 1H). ^{13}C NMR (126 MHz, $CDCl_3$) δ 203.42, 154.99, 154.80, 149.06, 142.85, 132.23, 131.79, 129.44, 121.07, 105.92, 102.62, 102.08, 47.22, 43.90. HRMS (ESI) *m/z* calcd for $C_{16}H_{12}BrO_3^+$ $[M + H]^+$ 330.9964, found 330.9973.

5,6-Dimethoxy-3-(3,4,5-trimethoxyphenyl)-2,3-dihydro-1*H*-inden-1-one (**11cc**)

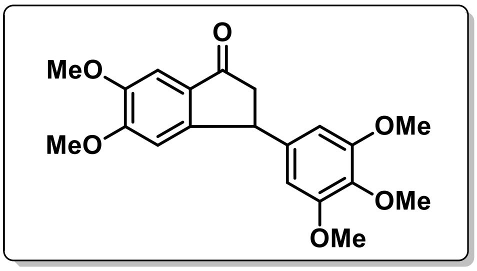

Purified by column chromatography (hexane/EtOAc 95:5–8:2) to give **11cc** (101 mg, 0,28 mmol, 94%) as a yellow solid. mp 137-139 ^oC. ^1H NMR (400 MHz, $CDCl_3$) δ 7.25 (s, 1H), 6.69 (s, 1H), 6.32 (s, 2H), 4.43 (dd, *J* = 7.6, 3.3 Hz, 1H), 3.96 (s, 3H), 3.89 (s, 3H), 3.85 (s, 3H), 3.81 (s, 6H), 3.21 (dd, *J* = 19.0, 7.7 Hz, 1H), 2.64 (dd, *J* = 19.0, 3.4 Hz, 1H). ^{13}C NMR (101 MHz, $CDCl_3$) δ 204.73, 156.02, 153.76, 153.03, 150.13, 144.79, 139.71, 137.13, 130.08, 107.64, 104.73, 103.90, 61.04, 56.55, 56.35, 47.40, 44.77, 29.77. HRMS (ESI) *m/z* calcd for $C_{20}H_{23}O_6^+$ $[M + H]^+$ 359.1489, found 359.1494.

3-(2,3-Dihydro-1,4-benzodioxin-6-yl)-4-ethyl-5,6-dimethoxy-2,3-dihydro-1*H*-inden-1-one (**11ae**)

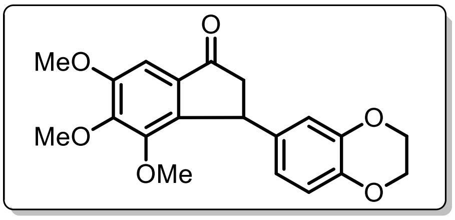

Purified by column chromatography (hexane/EtOAc 95:5–9:1) to give **11ae** (83 mg, 0,23 mmol, 78%) as a brown oil. ^1H NMR (500 MHz, $CDCl_3$) δ 7.09 (s, 1H), 6.79 (d, *J* = 8.2 Hz, 1H), 6.60 (d, *J* = 7.3 Hz, 2H), 4.50 (d, *J* = 6.8 Hz, 1H), 4.24 (s, 3H), 3.93 (s, 6H), 3.47 (s, 3H), 3.16 (dd, *J* = 19.2, 7.9 Hz, 1H), 2.58 (d, *J* = 19.3 Hz, 1H). ^{13}C NMR (126 MHz, $CDCl_3$) δ 205.40, 154.89, 150.40, 148.79, 144.53, 143.51, 142.19, 137.72, 132.18, 120.17, 117.26, 115.87, 100.29, 64.38, 64.31, 60.92, 60.17, 56.26, 47.29, 40.95. HRMS (ESI) *m/z* calcd for $C_{20}H_{21}O_6^+$ $[M + H]^+$ 357.1333, found 357.1335.

3-(3-Iodophenyl)-4,5,6-trimethoxy-2,3-dihydro-1*H*-inden-1-one (**11af**)

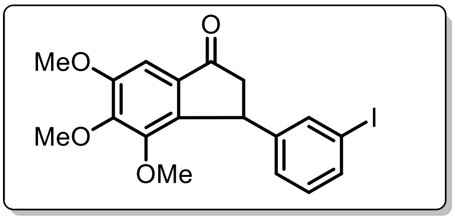

Purified by column chromatography (hexane/EtOAc 95:5–9:1) to give **11af** (93 mg, 0,22 mmol, 73%) as a yellow oil. ^1H NMR (500 MHz, $CDCl_3$) δ 7.57 (d, *J* = 7.5 Hz, 1H), 7.50 (s, 1H), 7.15 – 6.99 (m, 3H), 4.52 (d, *J* = 6.2 Hz, 1H), 3.95 (s, 3H), 3.93 (s, 3H), 3.43 (s, 3H), 3.19 (dd, *J* = 19.2, 8.0 Hz, 1H), 2.60 (d, *J* = 19.3

Hz, 1H). ^{13}C NMR (126 MHz, $CDCl_3$) δ 155.37, 150.48, 148.94, 146.98, 143.89, 136.57, 135.97, 132.39, 130.57, 126.73, 100.58, 94.67, 61.10, 60.30, 56.47, 47.02, 41.31. HRMS (ESI) *m/z* calcd for $C_{18}H_{18}IO_4^+$ [M + H]$^+$ 425.0244, found 425.0253.

References

1. Hackleöer, K.; Schnakenburg, G.; Waldvogel, S. R. *Eur. J. Org. Chem.* **2011,** 2011, 6314-6319. doi: 10.1002/ejoc.201100918.

2. Canterbury, D. P.; Frontier, A. J.; Um, J. M.; Cheong, P. H.-Y.; Goldfield, D. A.; Huhn, R. A.; Houk, K. N. *Org. Lett.* **2008**, *10*, 4597–4600. doi: 10.1021/ol8019154.

3. Hermange, P.; Gøgsig, T. M.; Lindhardt, A. T.; Taaning, R. H.; Skrydstrup, T. *Org. Lett.* **2011**, *13*, 2444–2447. doi: 10.1021/ol200686h.

4. Singh, S.; Trivedi, L.; Vasudev, P. G.; Passarell, D.; Negi, A. S. *New J. Chem.*, **2022**, *46*, 7850-7854. doi: 10.1039/D2NJ00783E.

5. Tang, M., -L.; Peng, P.; Liu, Z. -Y.; Zhang, J.; Yu, J. -M.; Sun, X. *Chem. – Eur. J.* **2016**, *22*, 14535-14539. doi: 10.1002/chem.201603664.

^{1}H NMR and ^{13}C NMR spectra

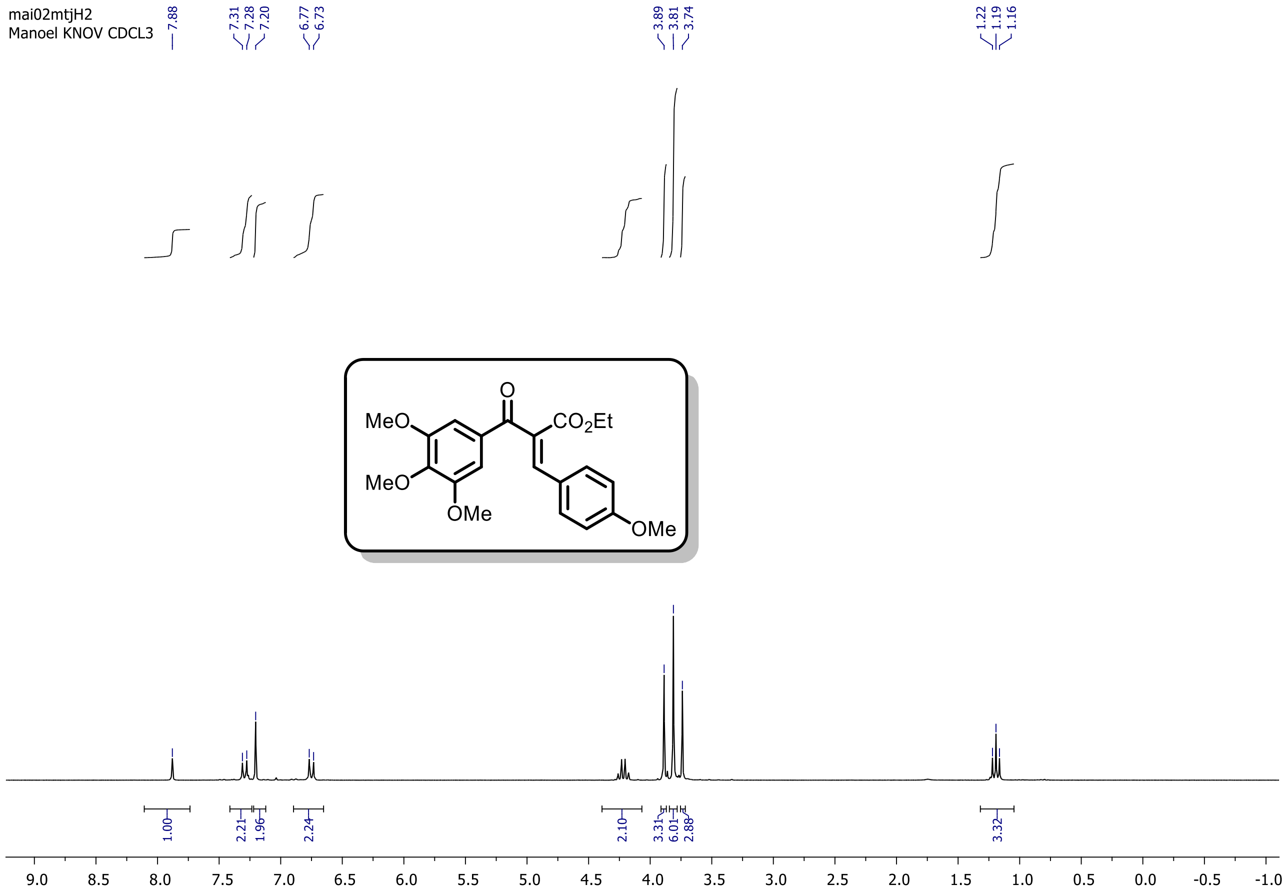

Figure S1. ^{1}H NMR spectrum (250 MHz, $CDCl_3$) of compound **9aa**.

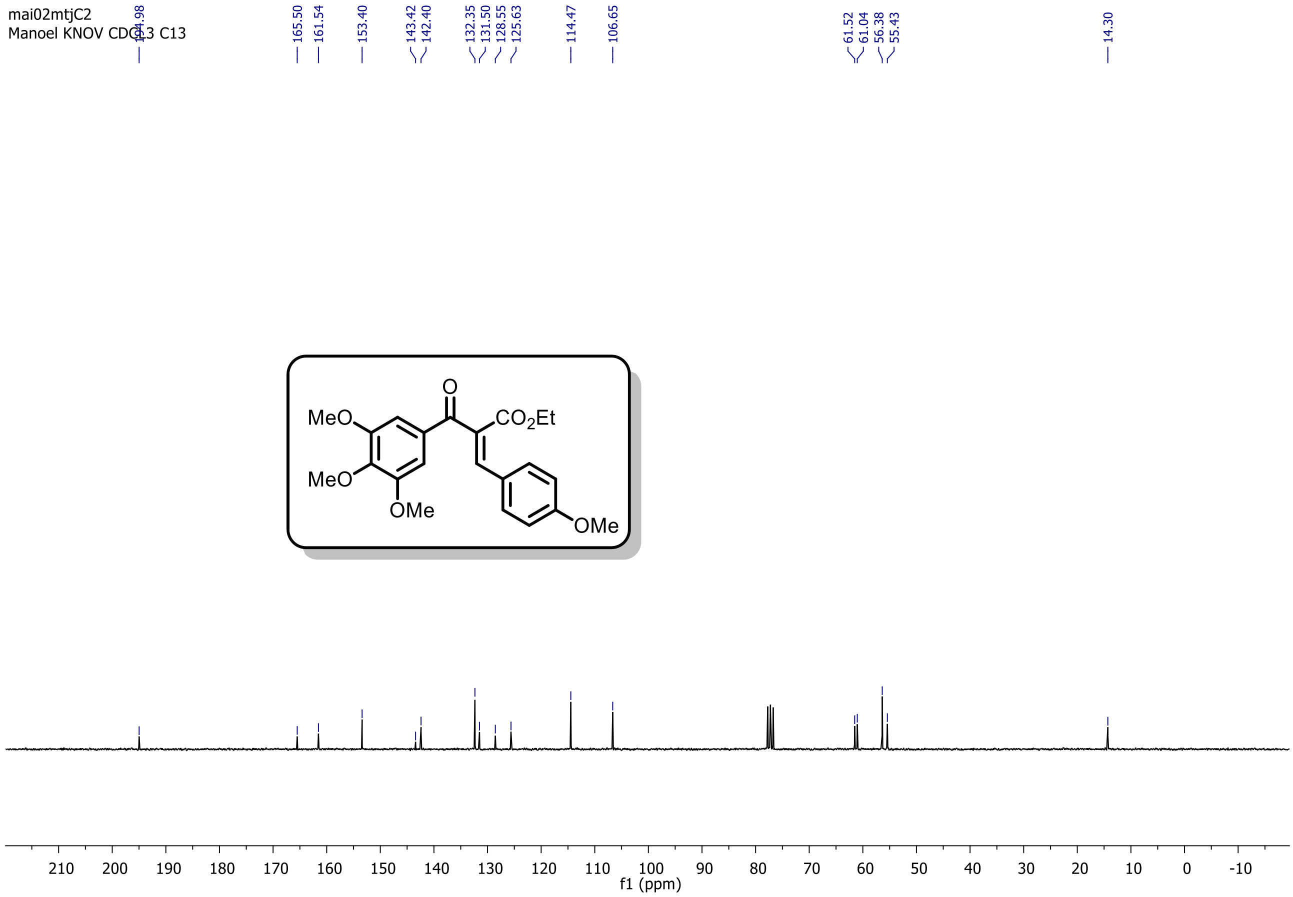

Figure S2. ^{13}C NMR spectrum (63 MHz, $CDCl_3$) of compound **9aa**.

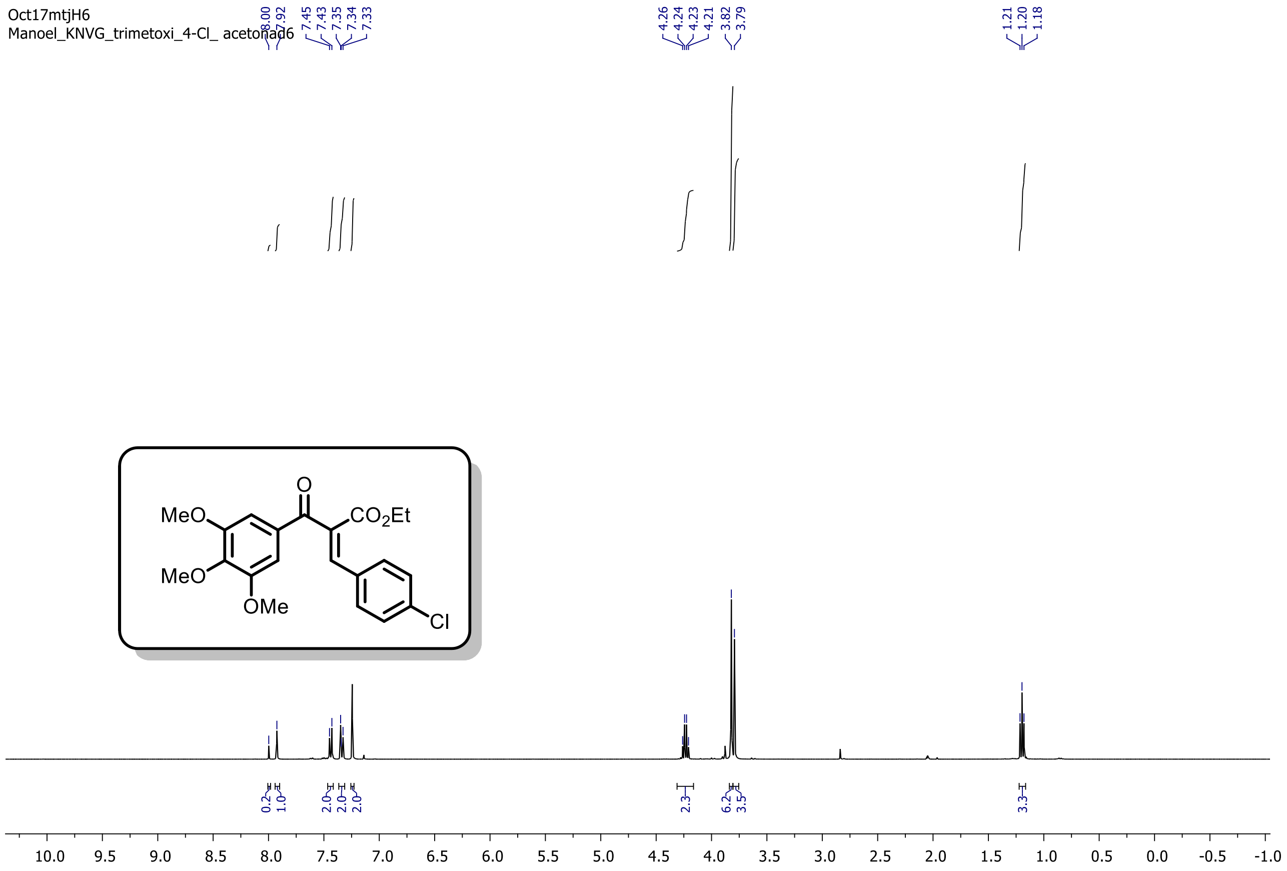

Figure S3. ^{1}H NMR spectrum (400 MHz, $CDCl_3$) of compound **9ab**.

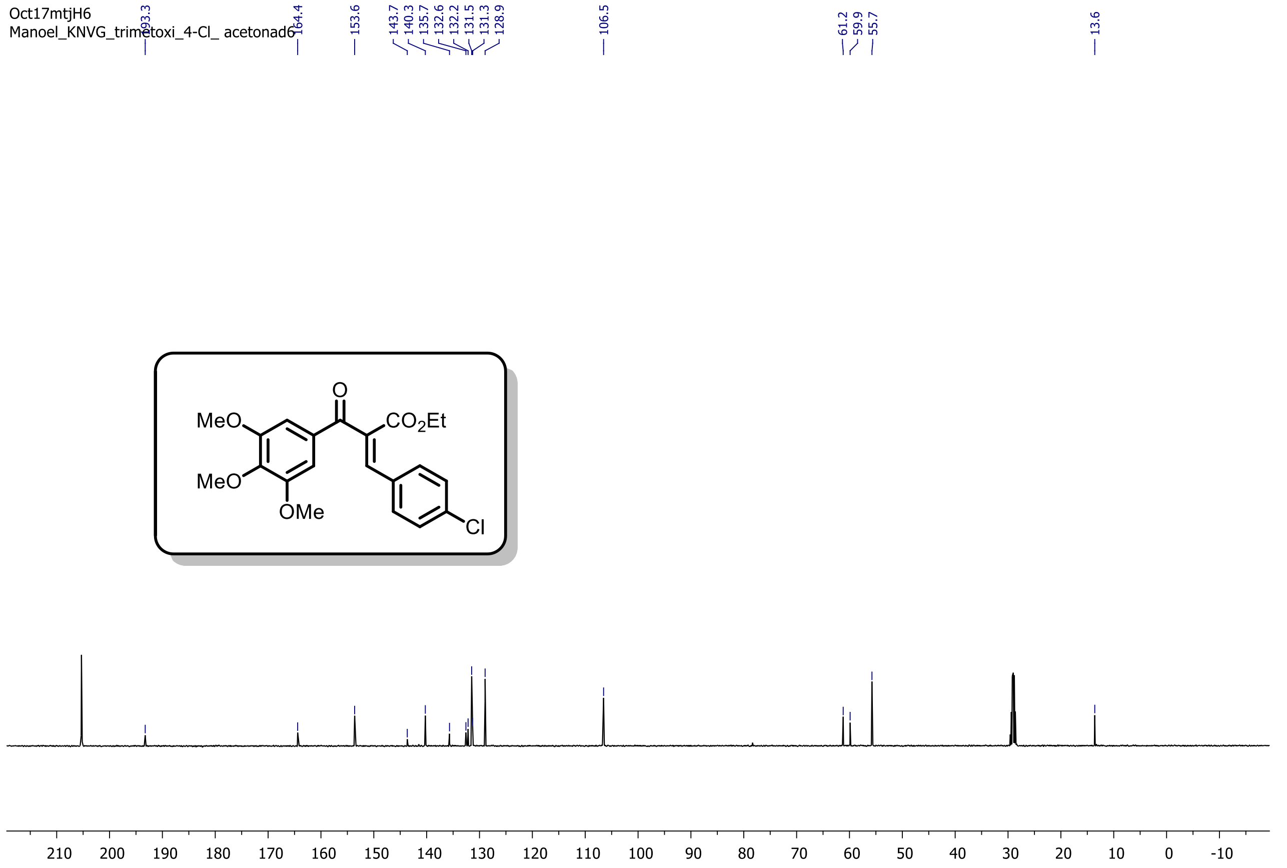

Figure S4. ^{13}C NMR spectrum (101 MHz, $CDCl_3$) of compound **9ab**.

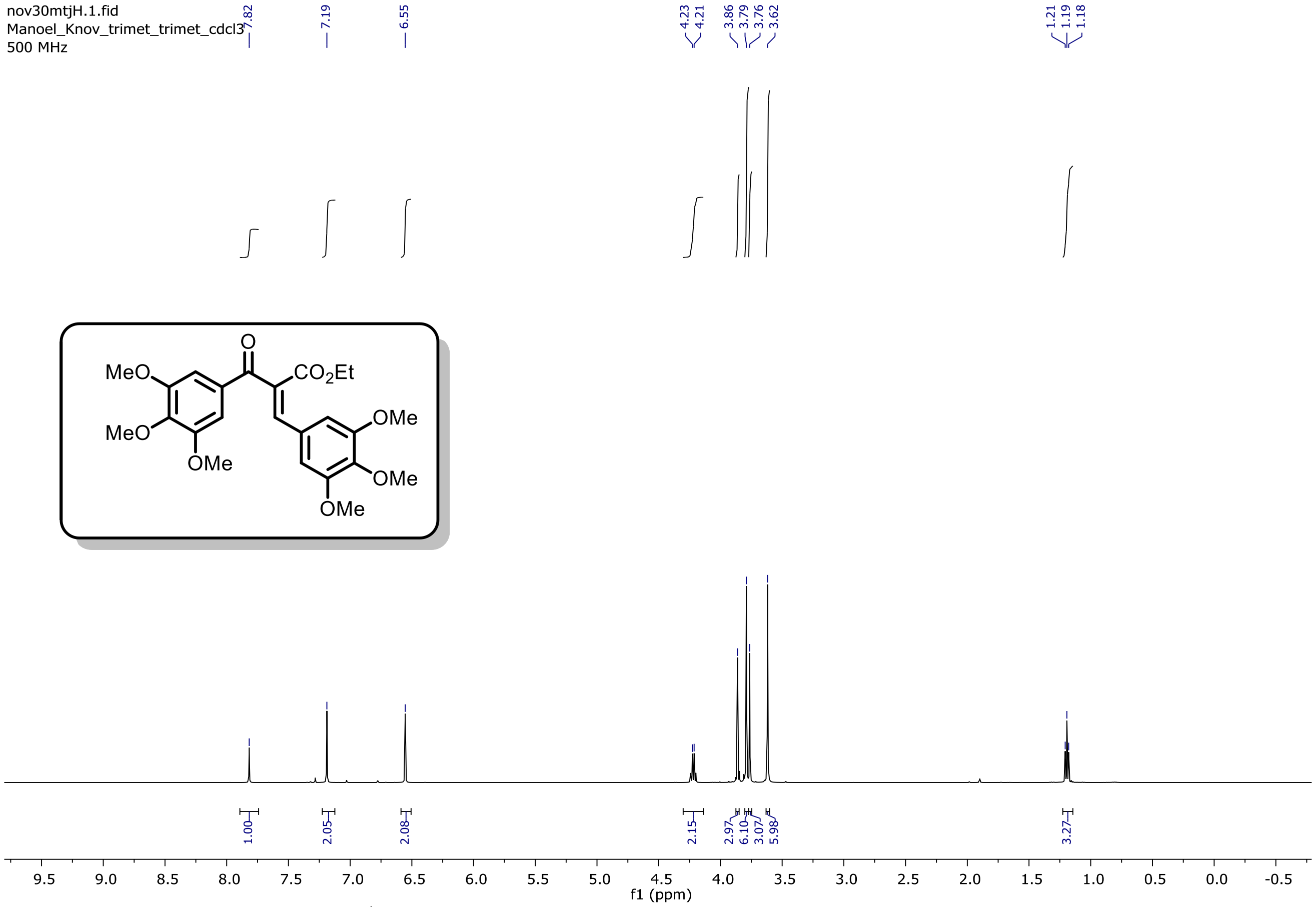

Figure S5. ^{1}H NMR spectrum (500 MHz, $CDCl_3$) of compound **9ac**.

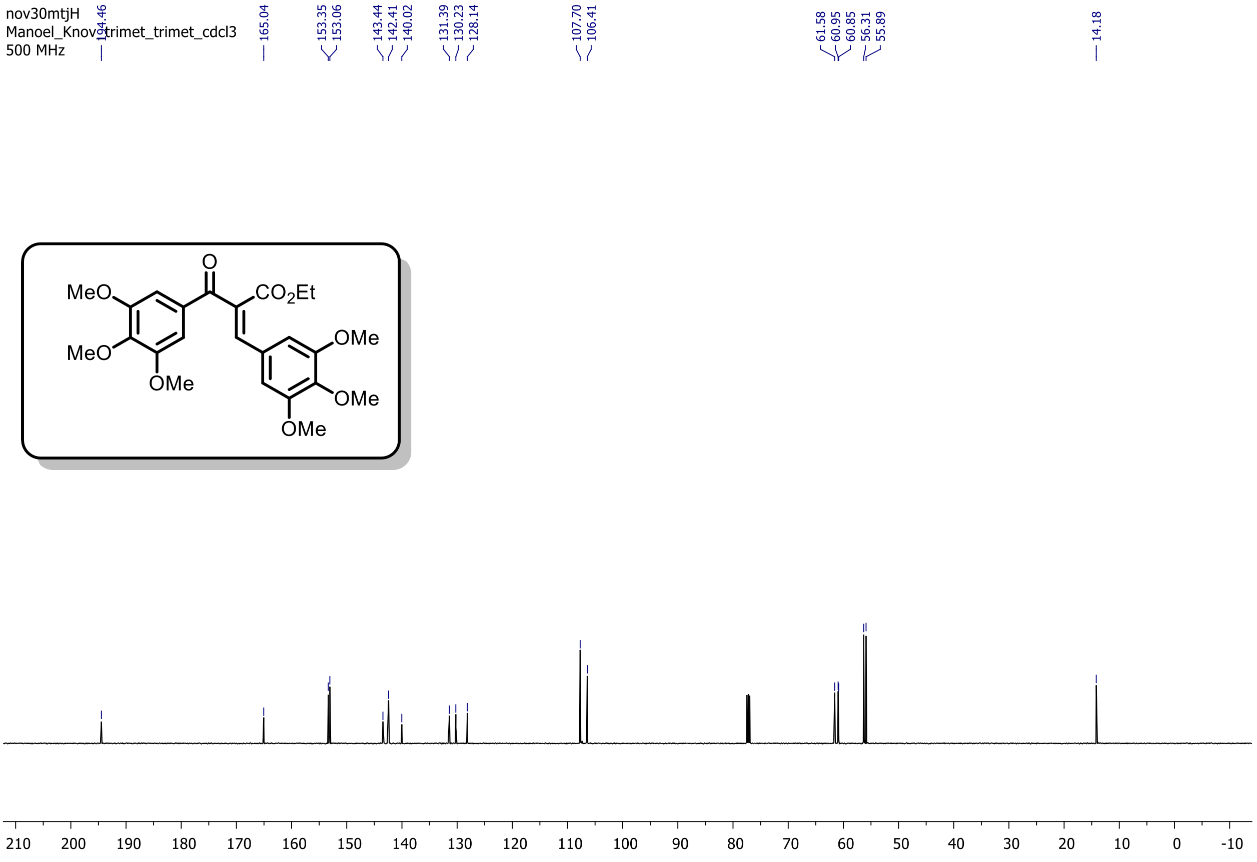

Figure S6. ^{13}C NMR spectrum (126 MHz, $CDCl_3$) of compound **9ac**.

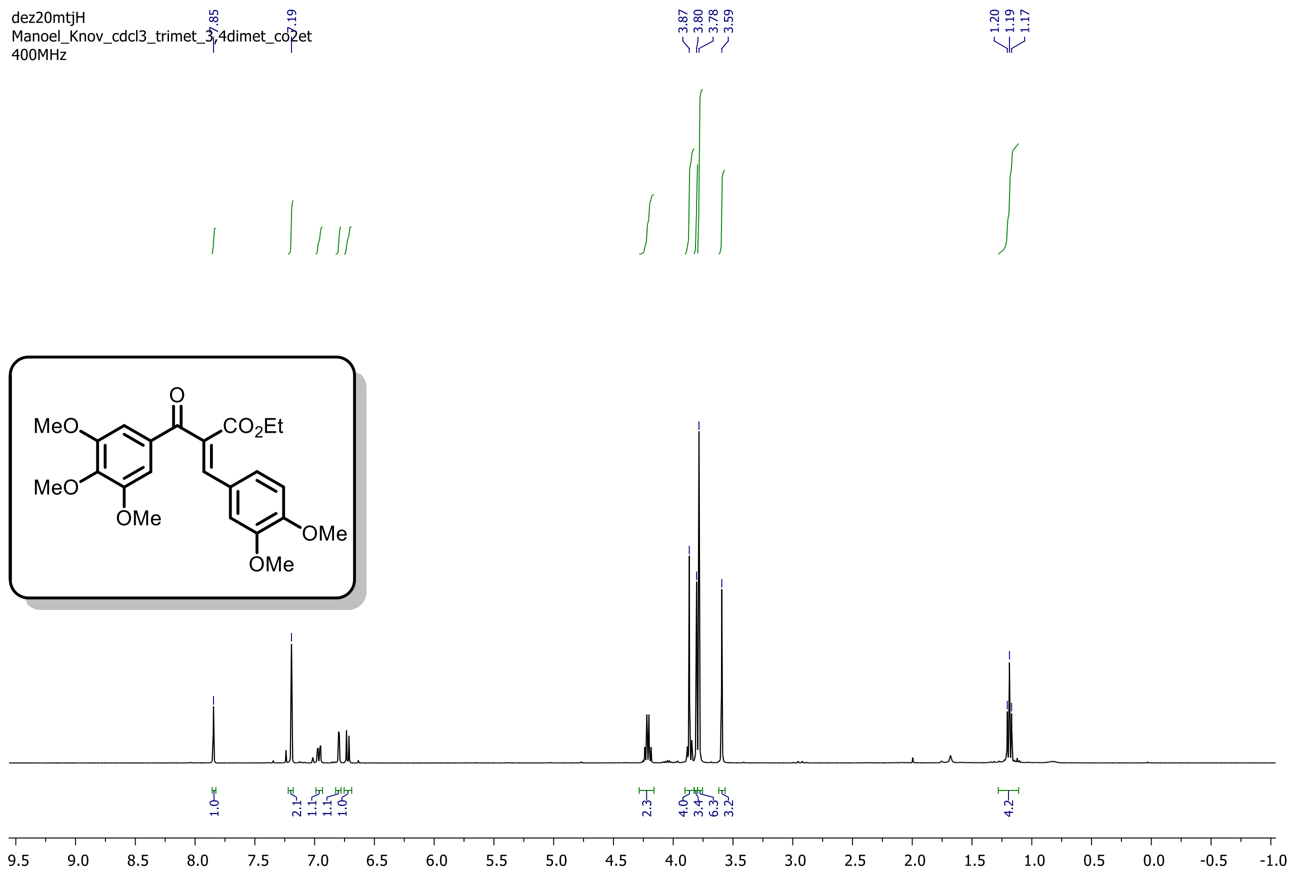

Figure S7. ^{1}H NMR spectrum (400 MHz, $CDCl_3$) of compound **9ad**.

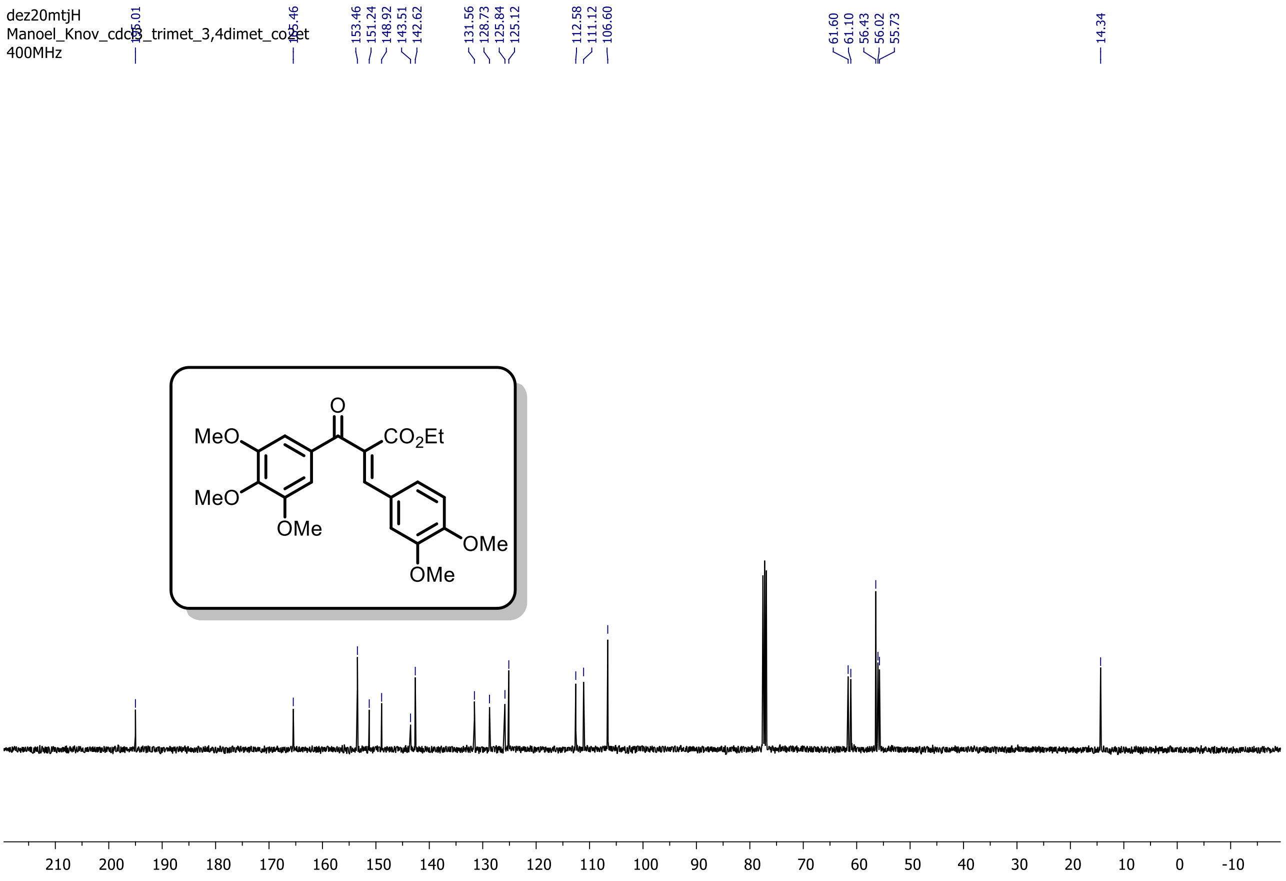

Figure S8. ^{13}C NMR spectrum (101 MHz, $CDCl_3$) of compound **9ad**.

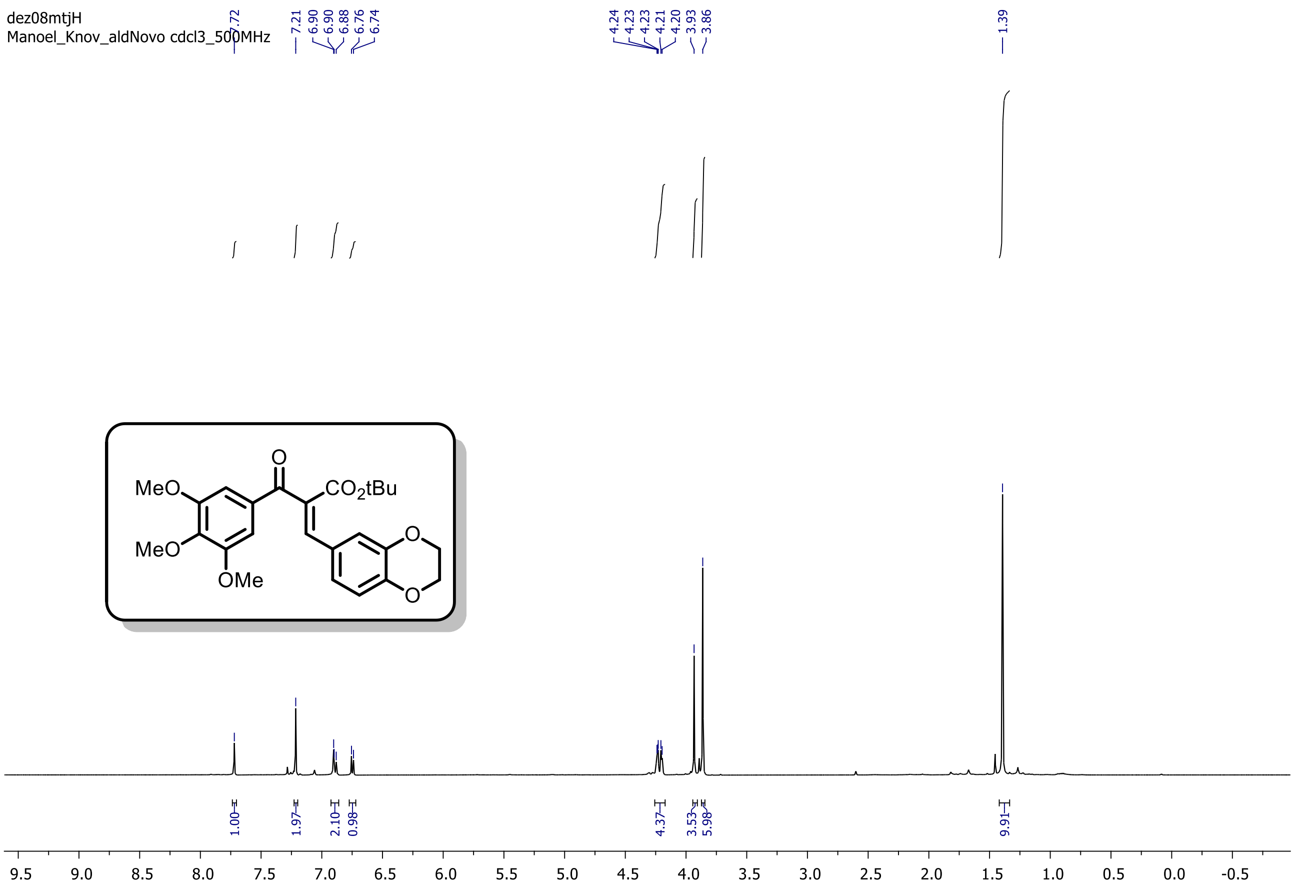

Figure S9. ^{1}H NMR spectrum (500 MHz, $CDCl_3$) of compound **9ae**.

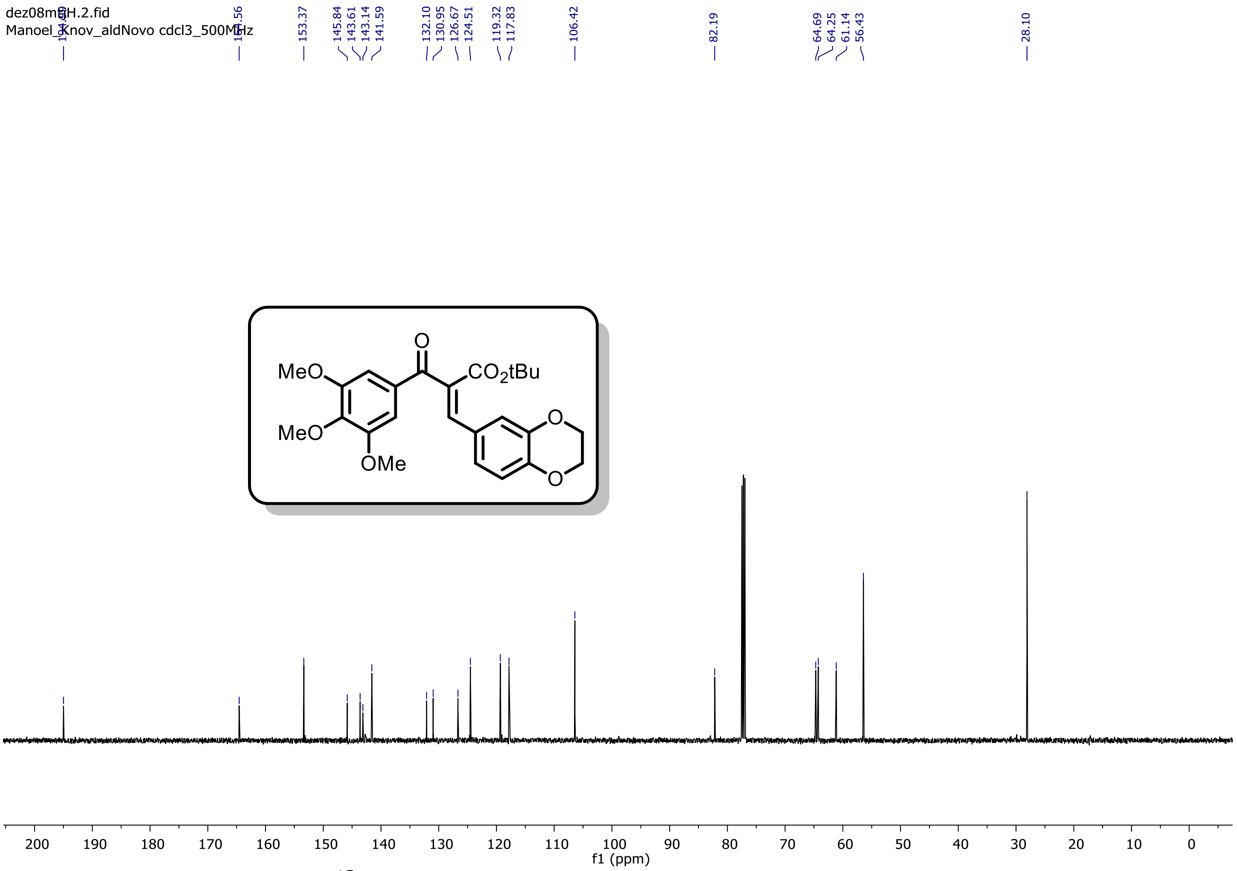

Figure S10-. ^{13}C NMR spectrum (126 MHz, $CDCl_3$) of compound **9ae**.

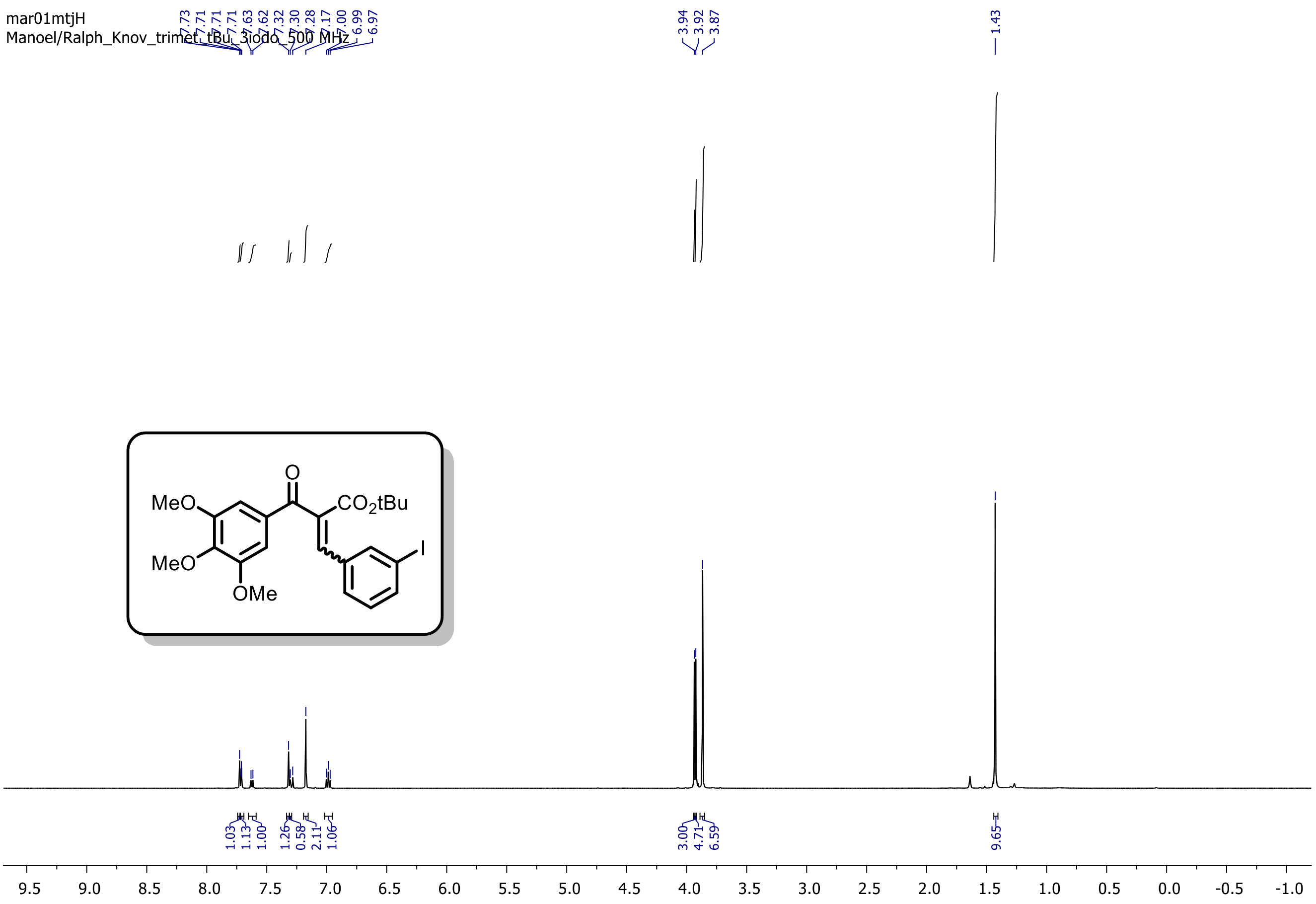

Figure S11. ^{1}H NMR spectrum (500 MHz, $CDCl_3$) of compound **9af**.

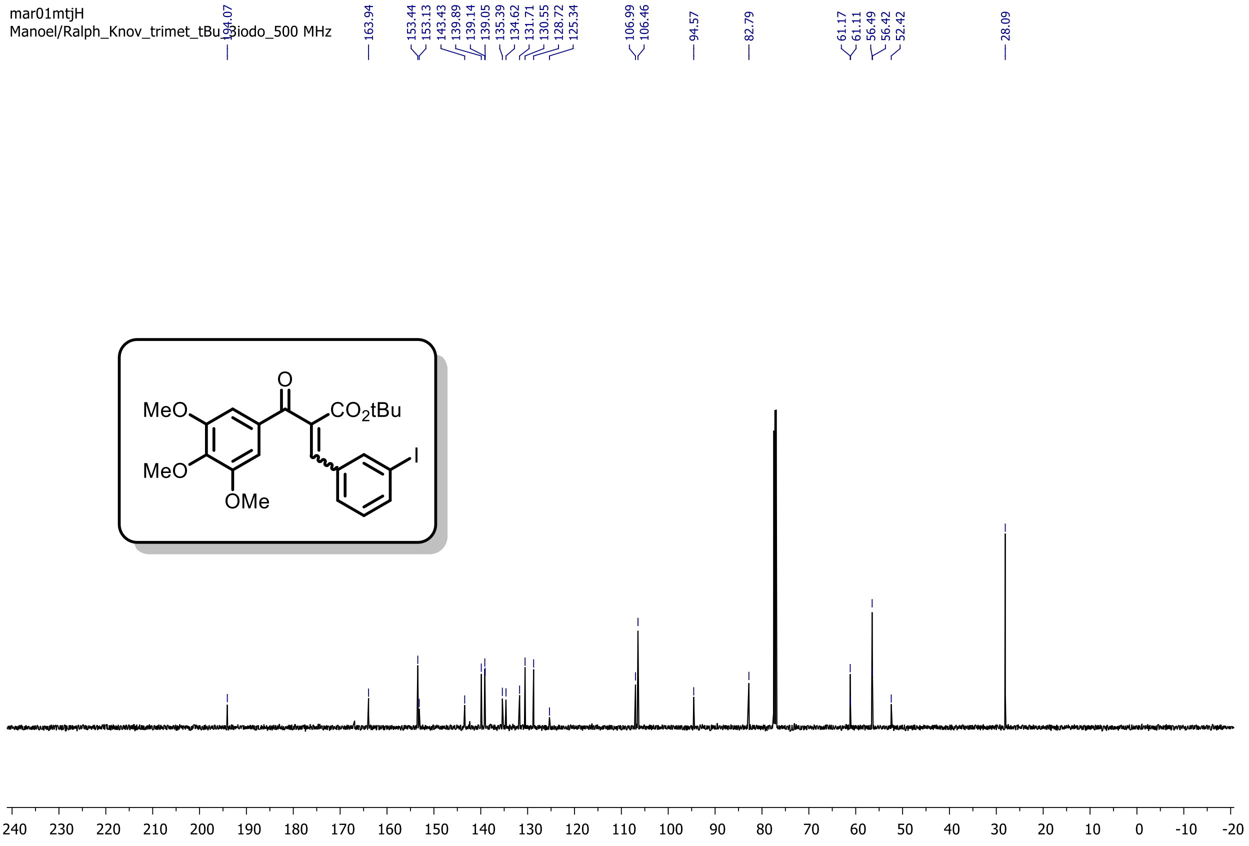

Figure S12. ^{13}C NMR spectrum (126 MHz, $CDCl_3$) of compound **9af**.

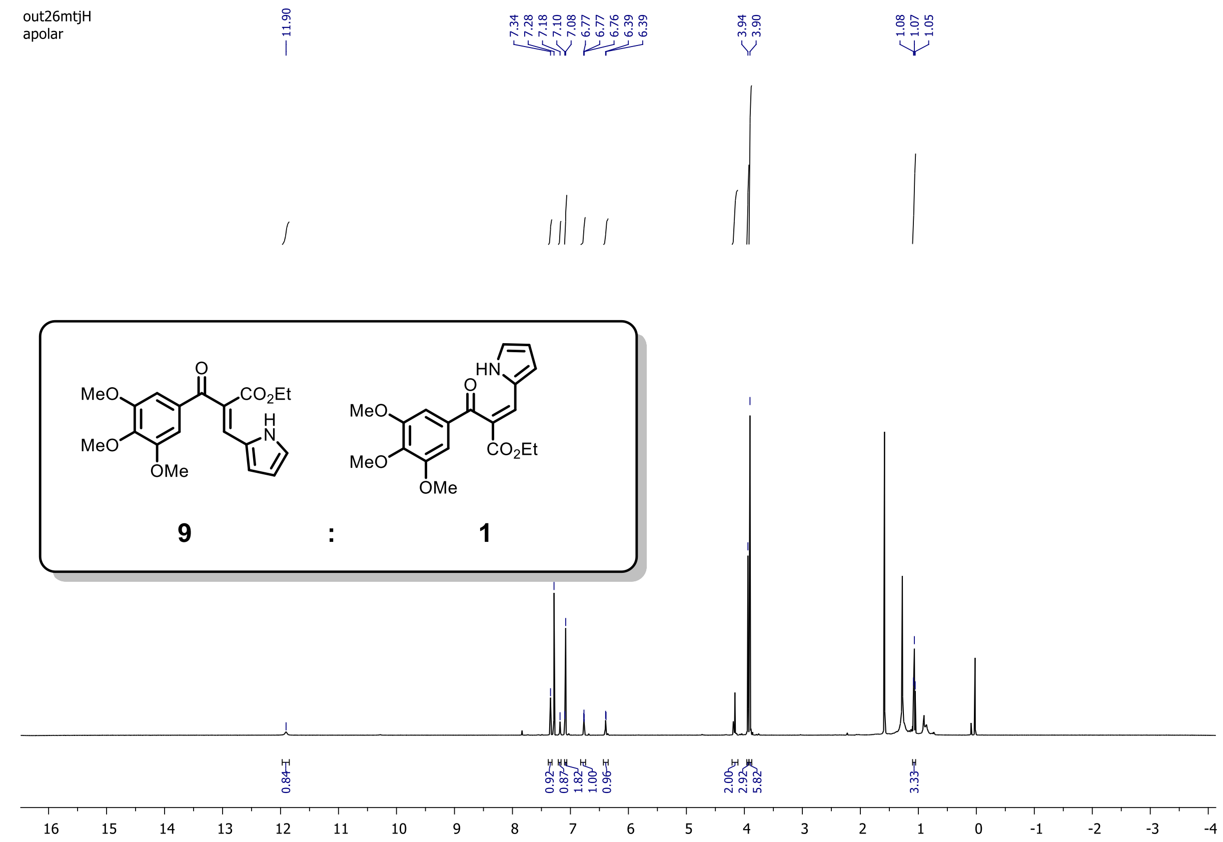

Figure 13. ^{1}H NMR spectrum (500 MHz, $CDCl_3$) of compound **9ag**.

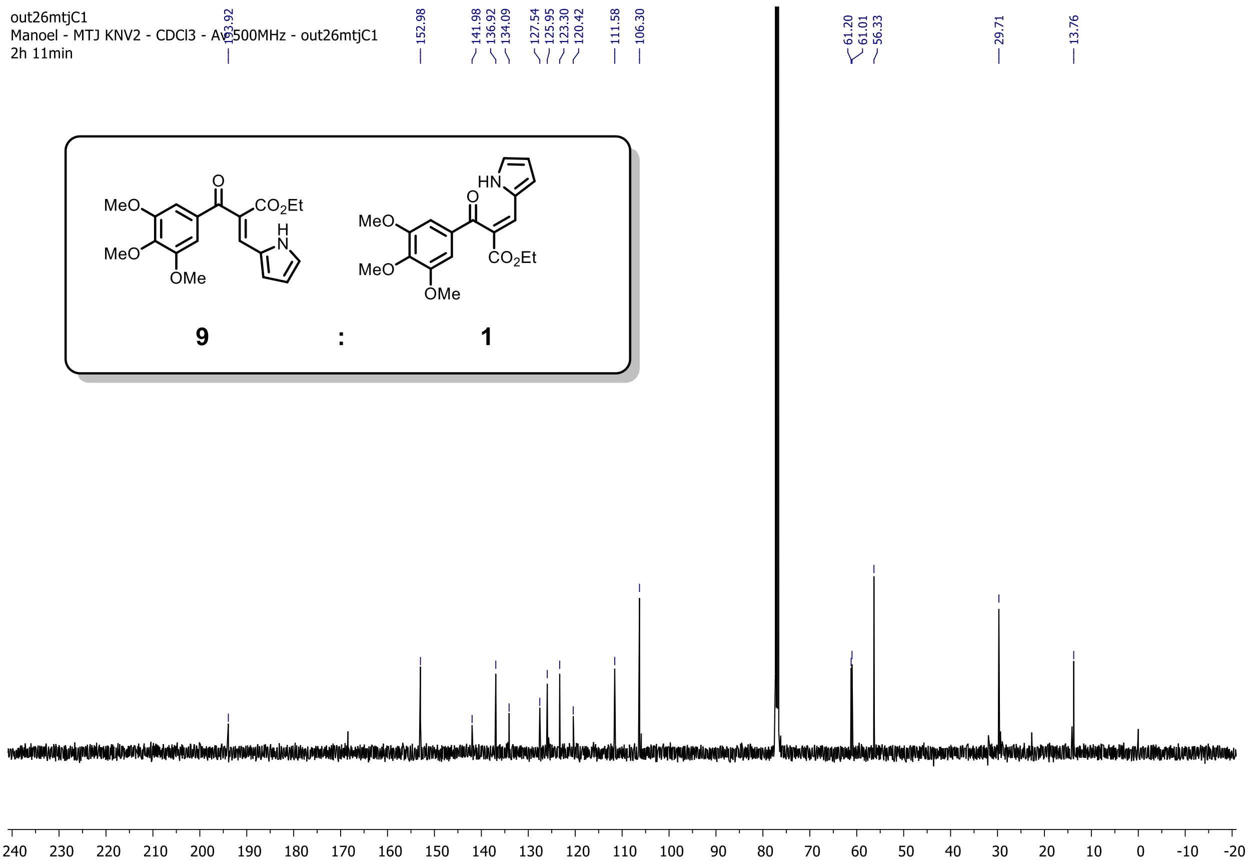

Figure S14. ^{13}C NMR spectrum (126 MHz, $CDCl_3$) of compound **9ag**.

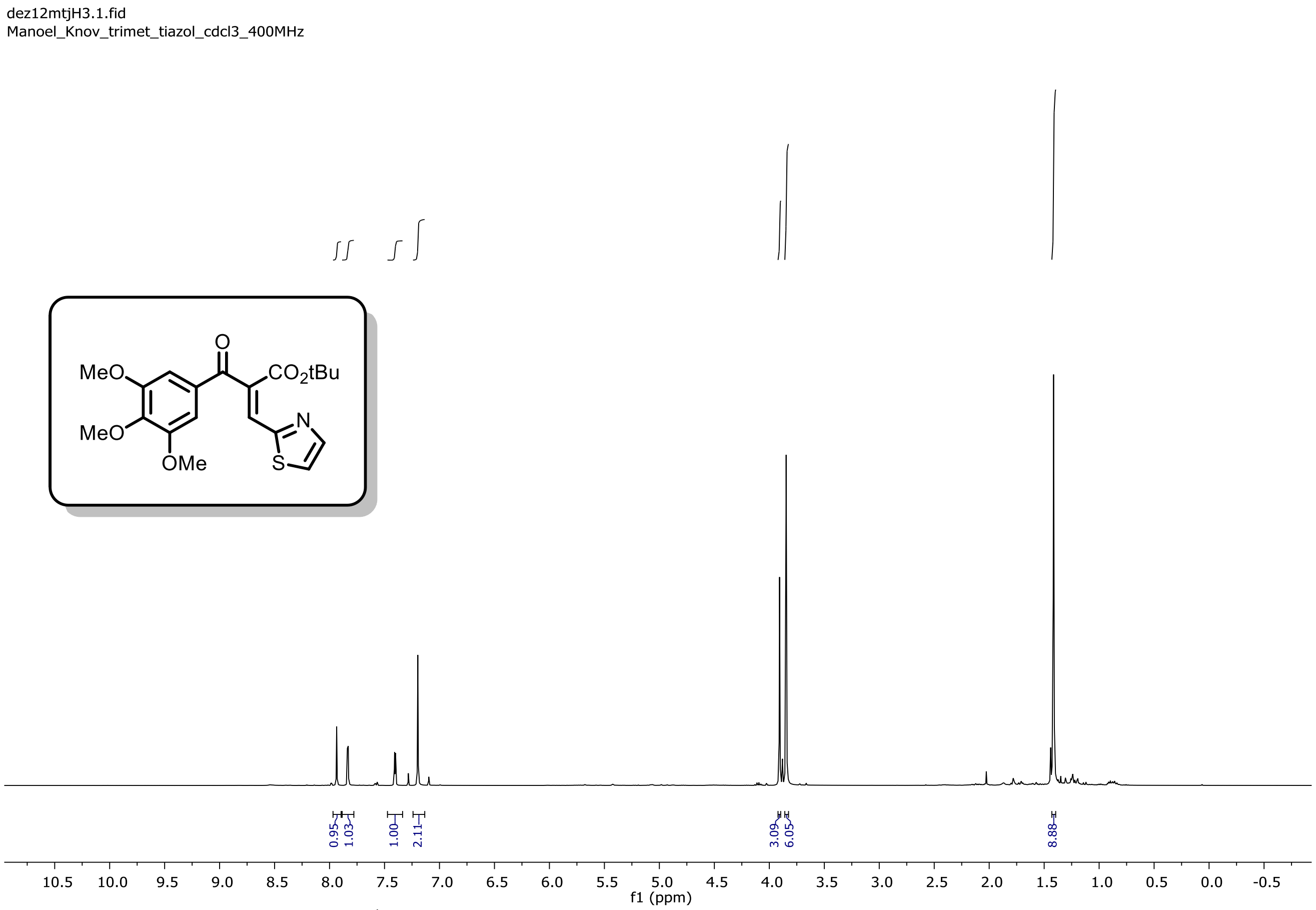

Figure S15. ^{1}H NMR spectrum (400 MHz, $CDCl_3$) of compound **9ah**.

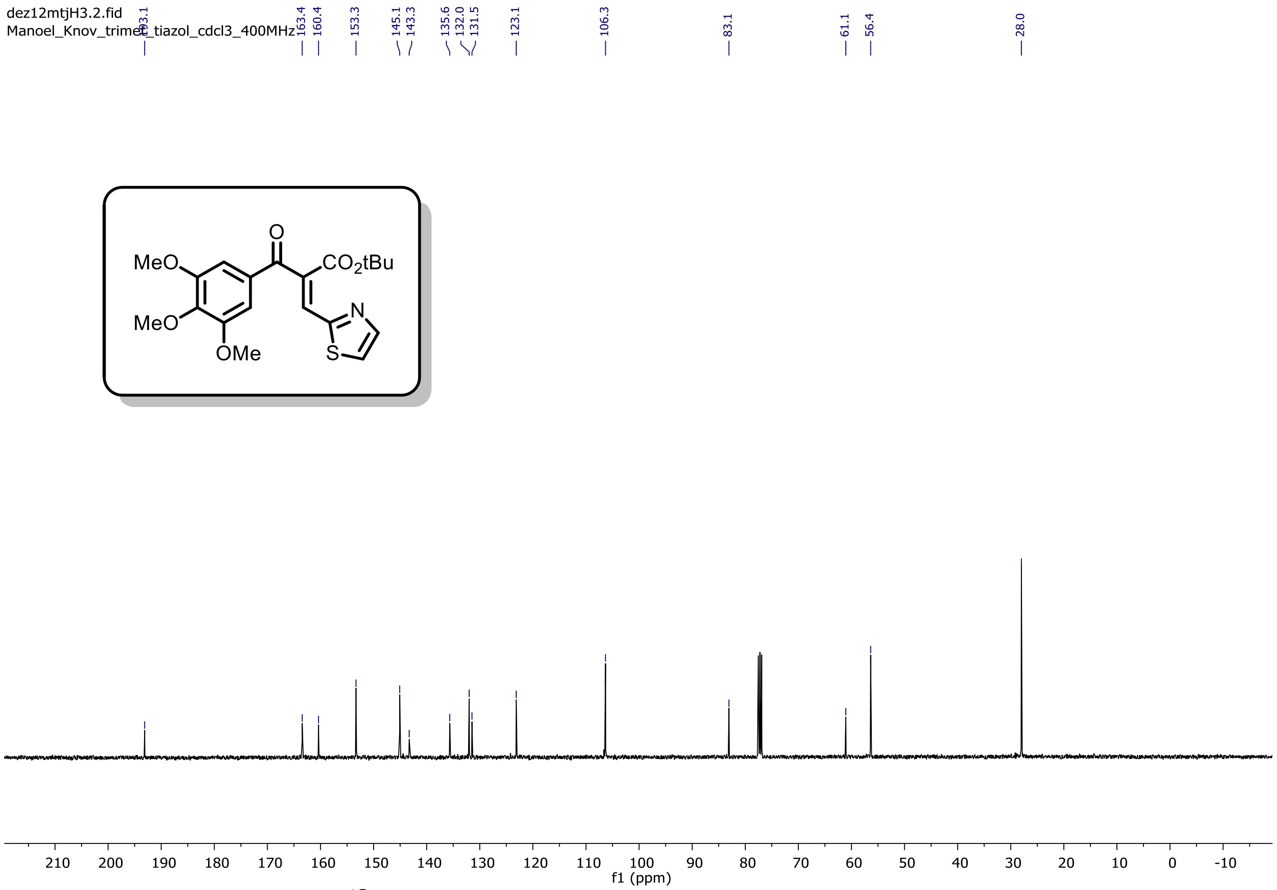

Figure S16. ^{13}C NMR spectrum (101 MHz, $CDCl_3$) of compound **9ah**.

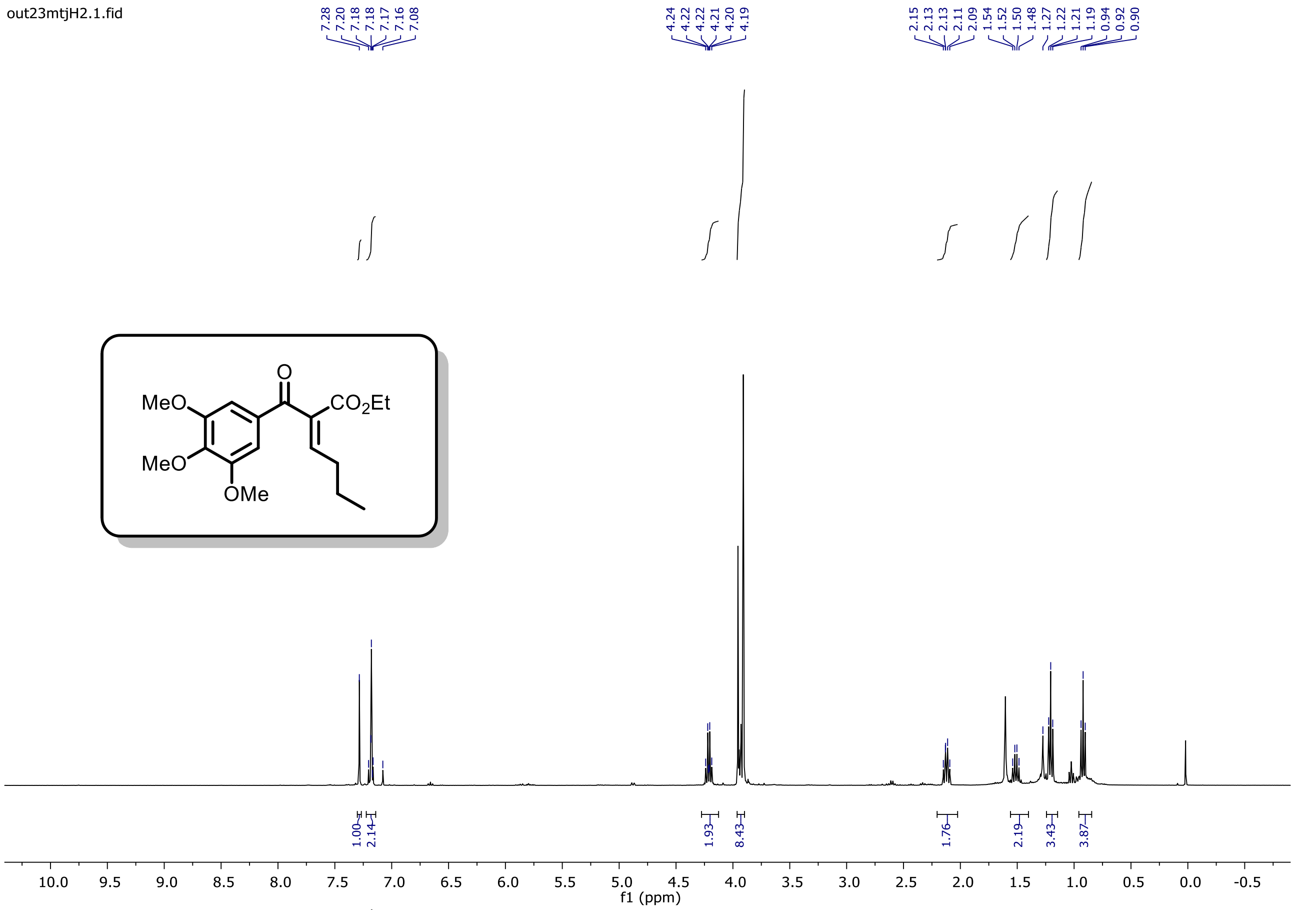

Figure S17. ^{1}H NMR spectrum (400 MHz, $CDCl_3$) of compound **9ai**.

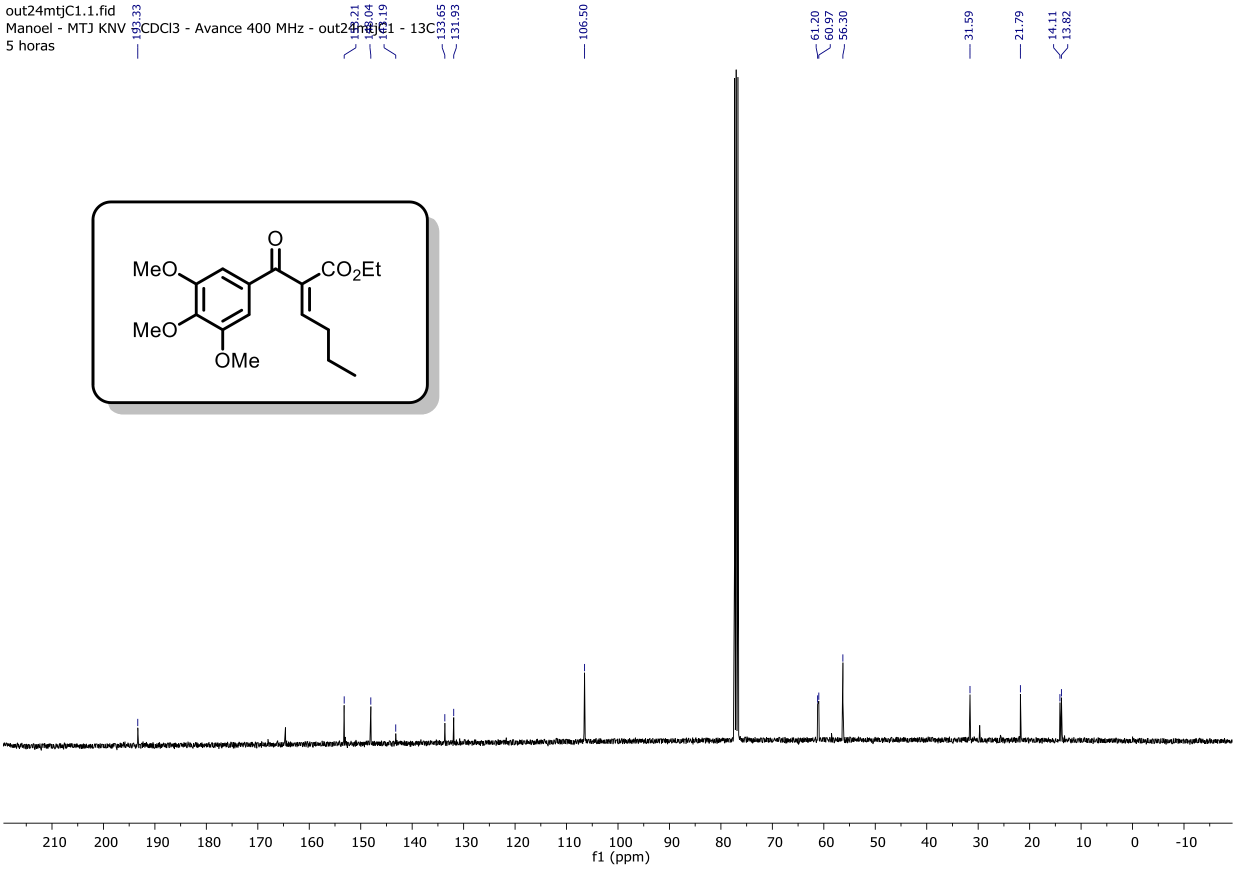

Figure S18. ^{13}C NMR spectrum (101 MHz, $CDCl_3$) of compound **9ai**.

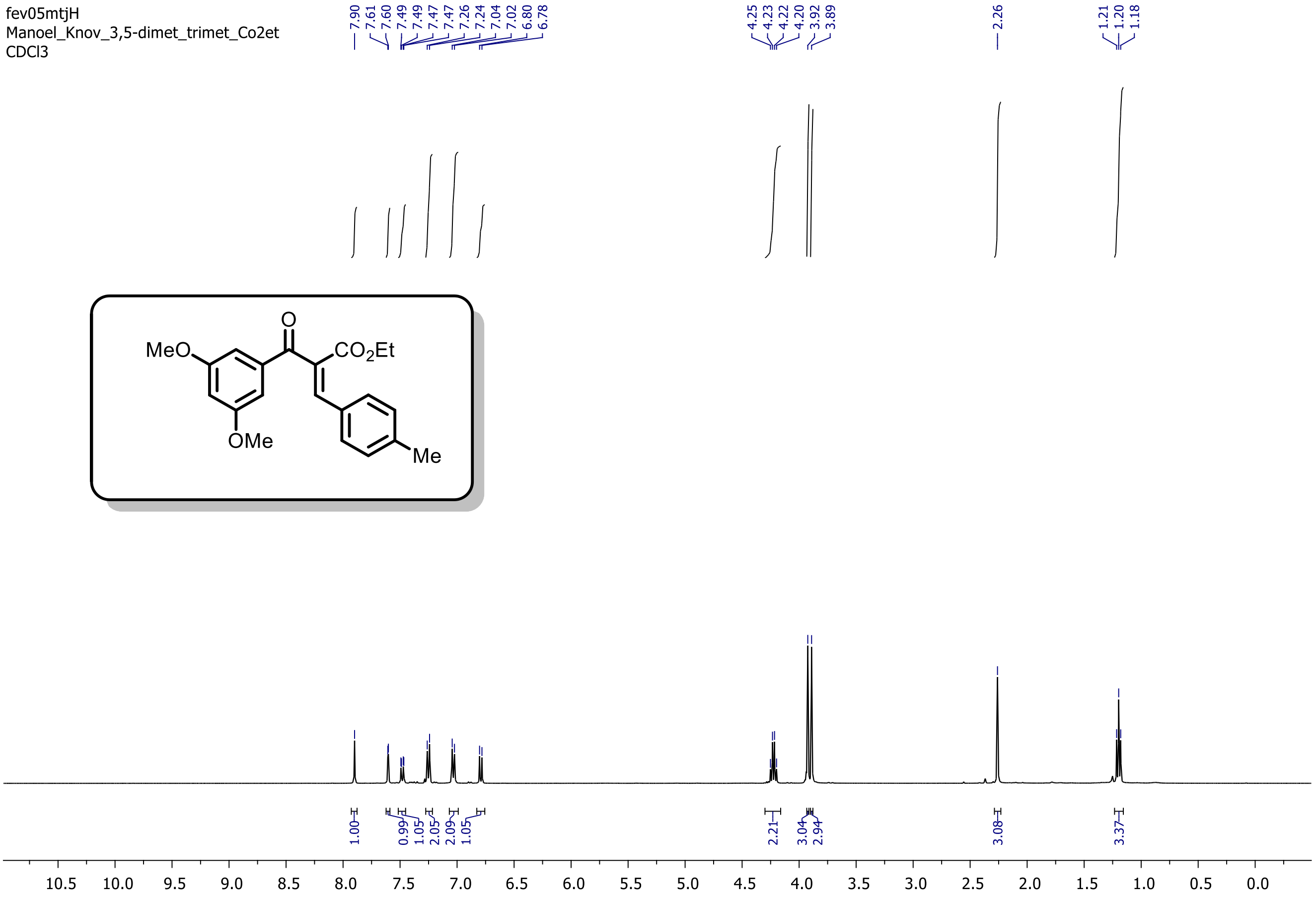

Figure S19. ^{1}H NMR spectrum (400 MHz, $CDCl_3$) of compound **9bj**.

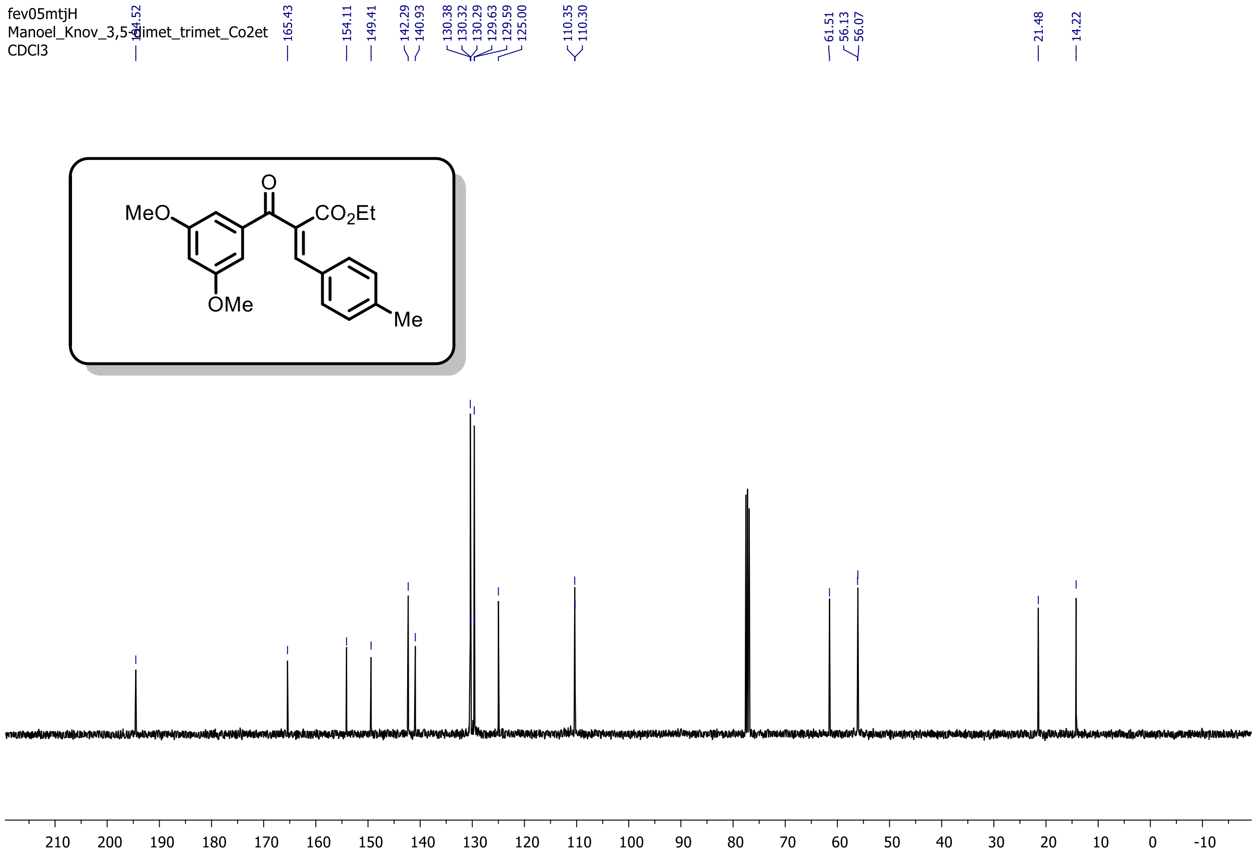

Figure S20. ^{13}C NMR spectrum (101 MHz, $CDCl_3$) of compound **9bj**.

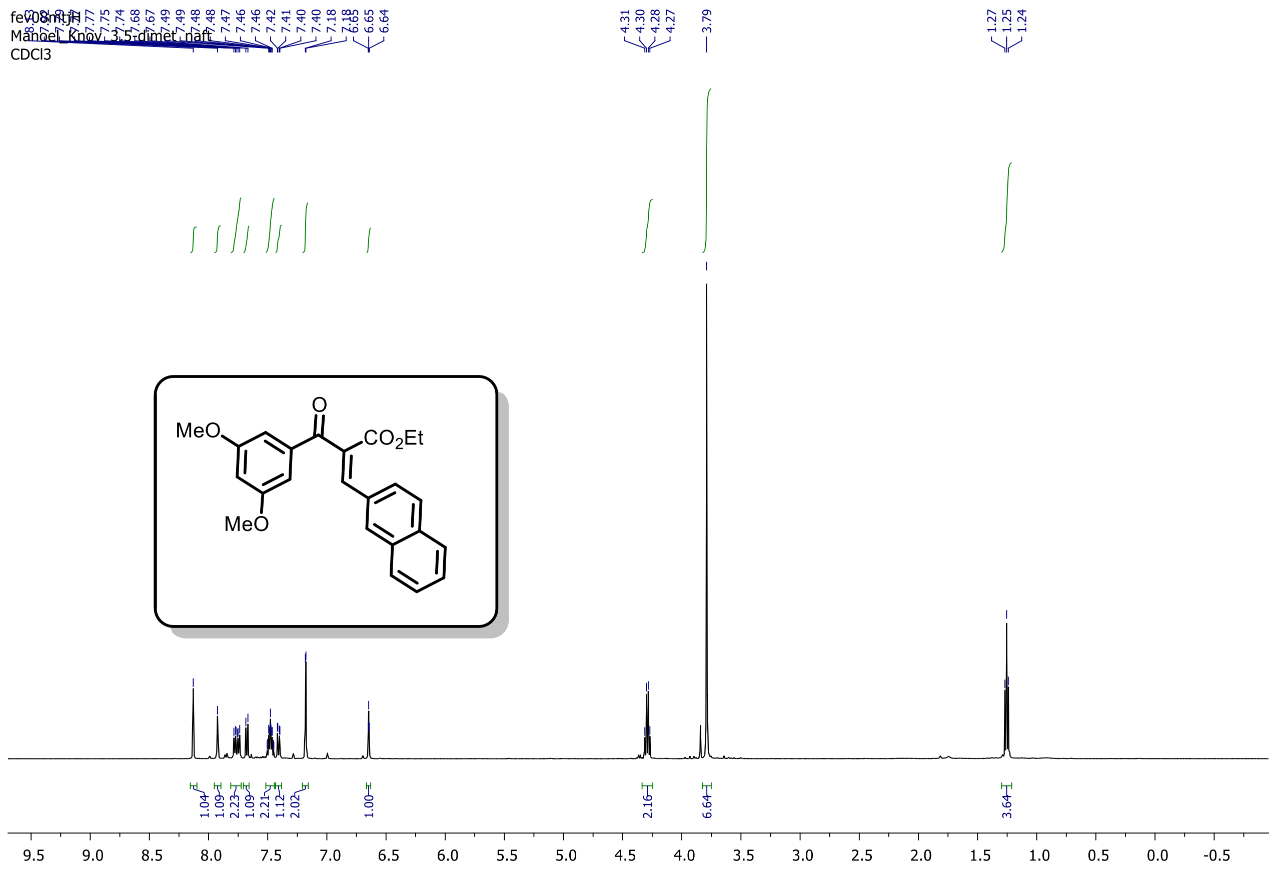

Figure S21. ^{1}H NMR spectrum (500 MHz, $CDCl_3$) of compound **9bk**.

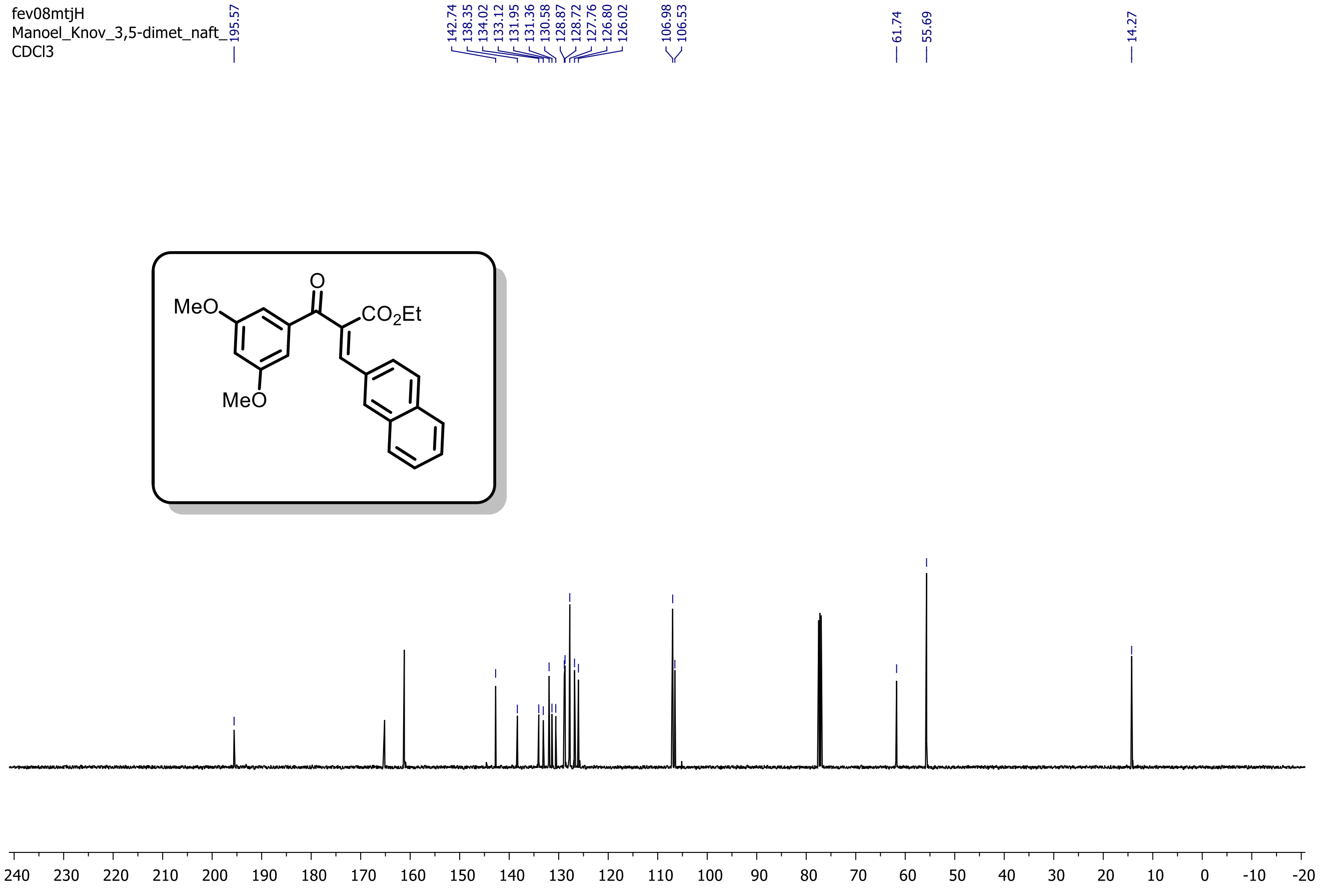

Figure S22. ^{13}C NMR spectrum (126 MHz, $CDCl_3$) of compound **9bk**.

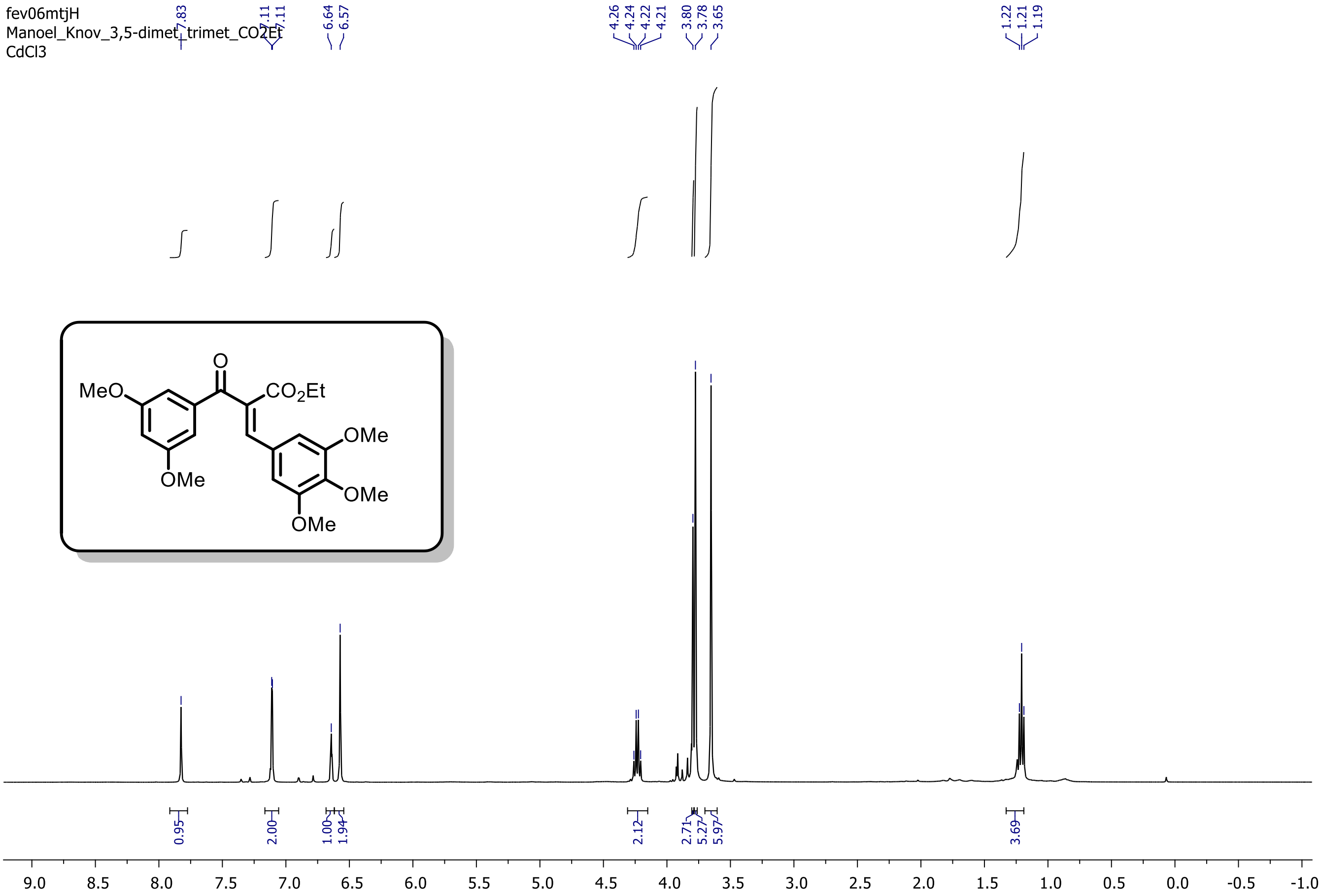

Figure S23. ^{1}H NMR spectrum (400 MHz, $CDCl_3$) of compound **9bc**.

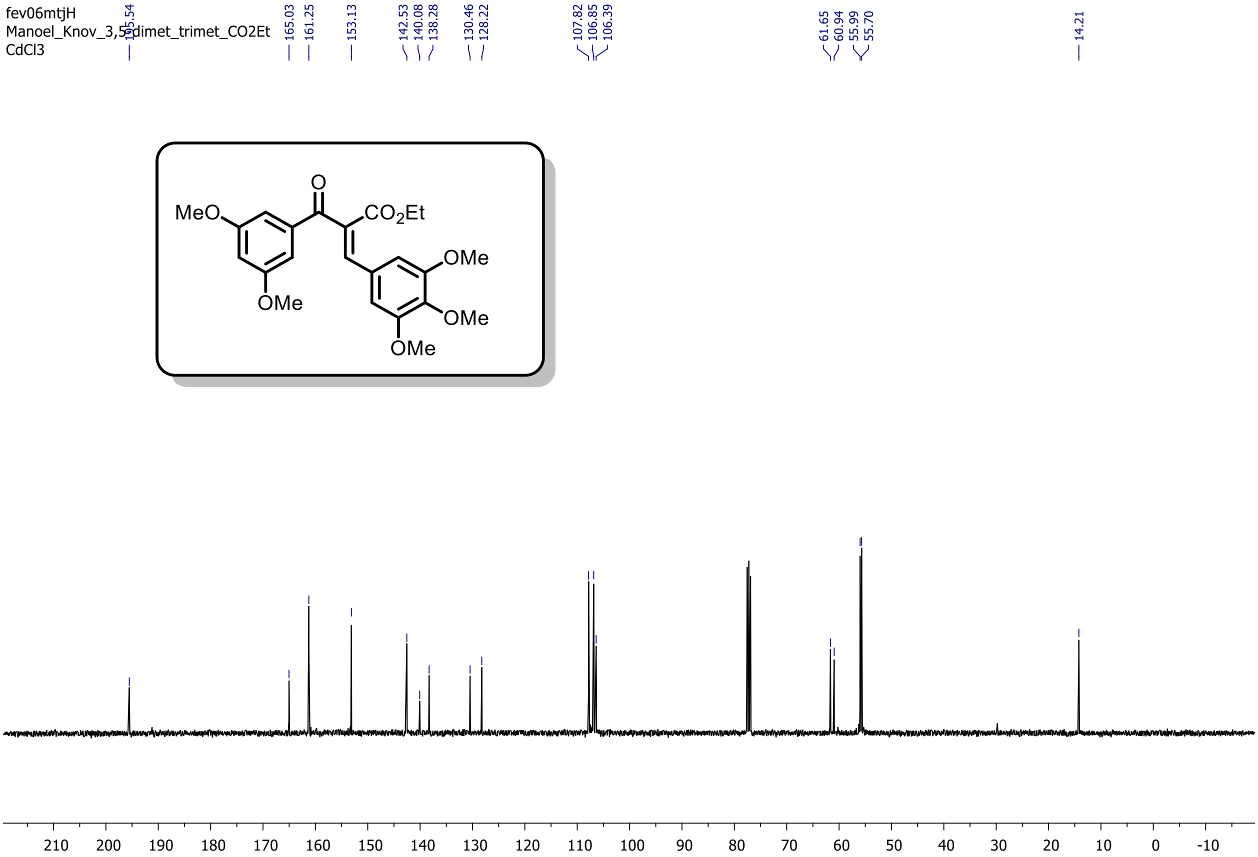

Figure S24. ^{13}C NMR spectrum (101 MHz, $CDCl_3$) of compound **9bc**.

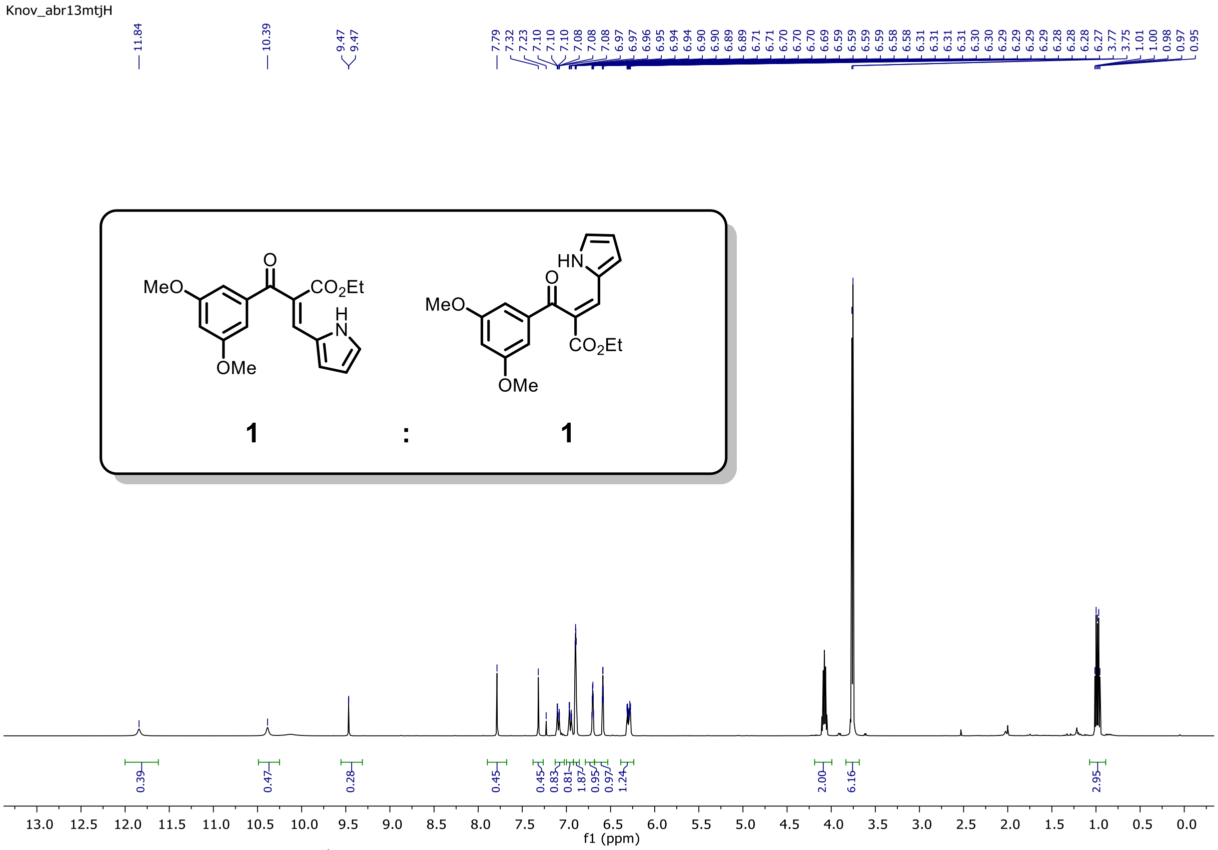

Figure S25. ^{1}H NMR spectrum (500 MHz, $CDCl_3$) of compound **9bg**.

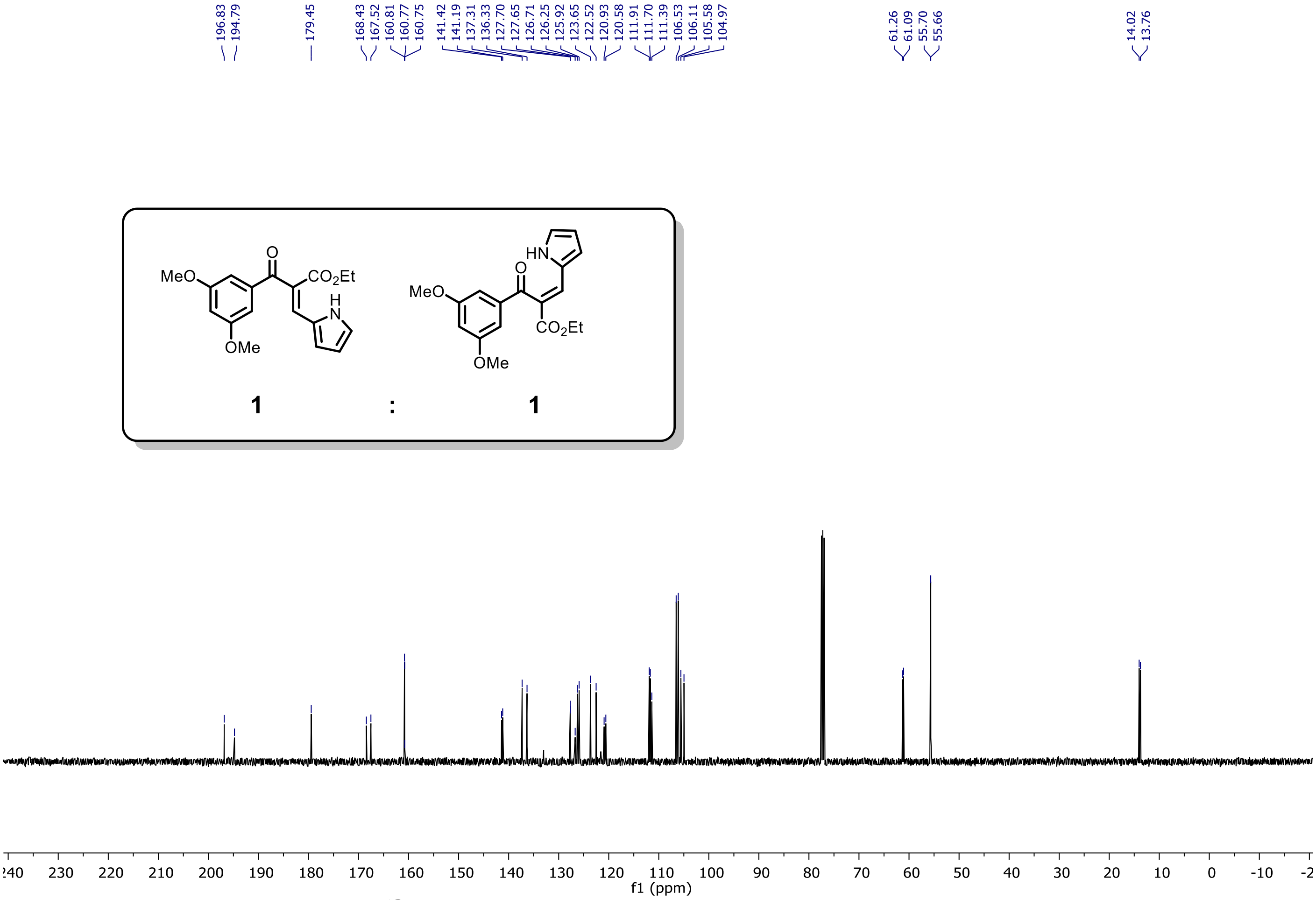

Figure S26. ^{13}C NMR spectrum (126 MHz, $CDCl_3$) of compound **9bg**.

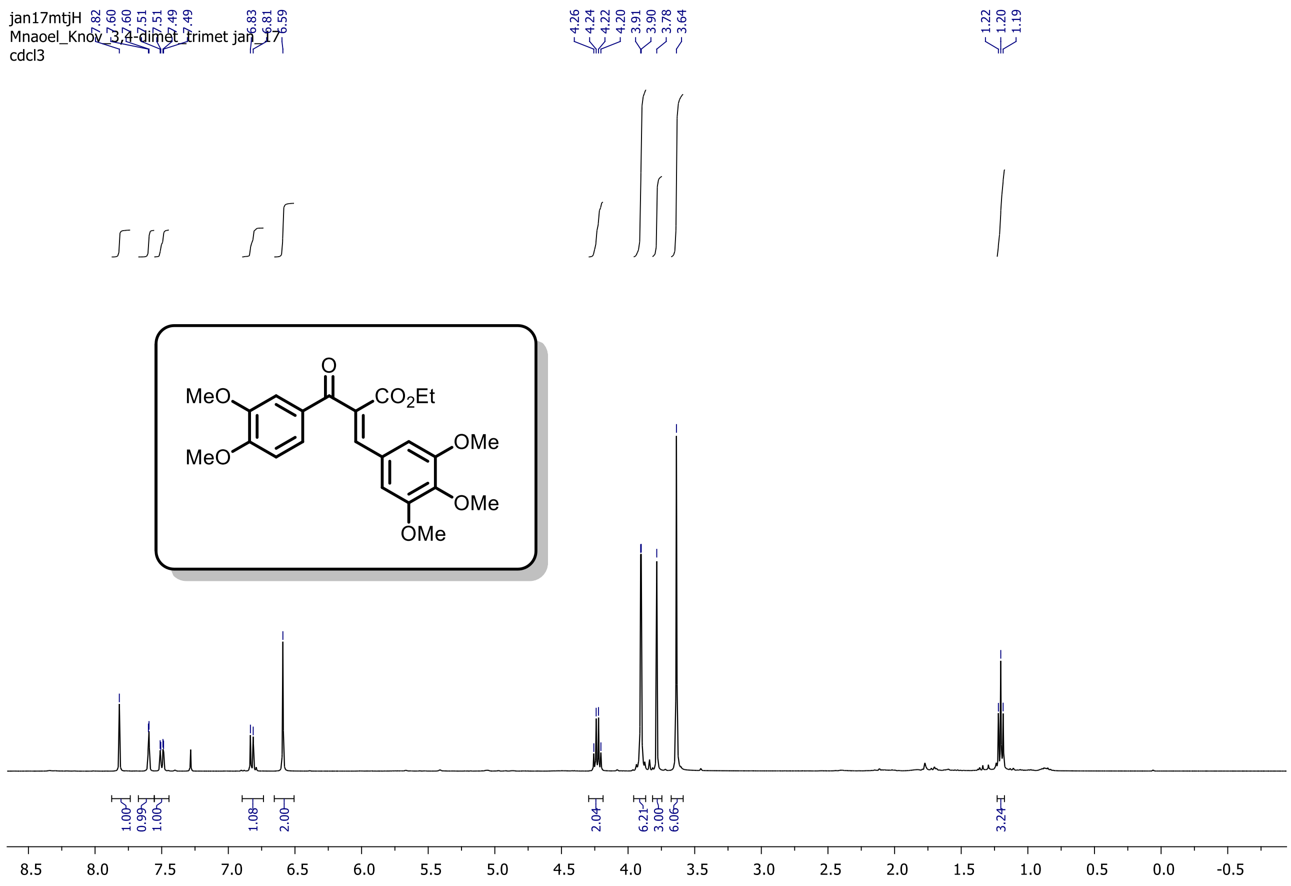

Figure S27. ^{1}H NMR spectrum (400 MHz, $CDCl_3$) of compound **9cc**.

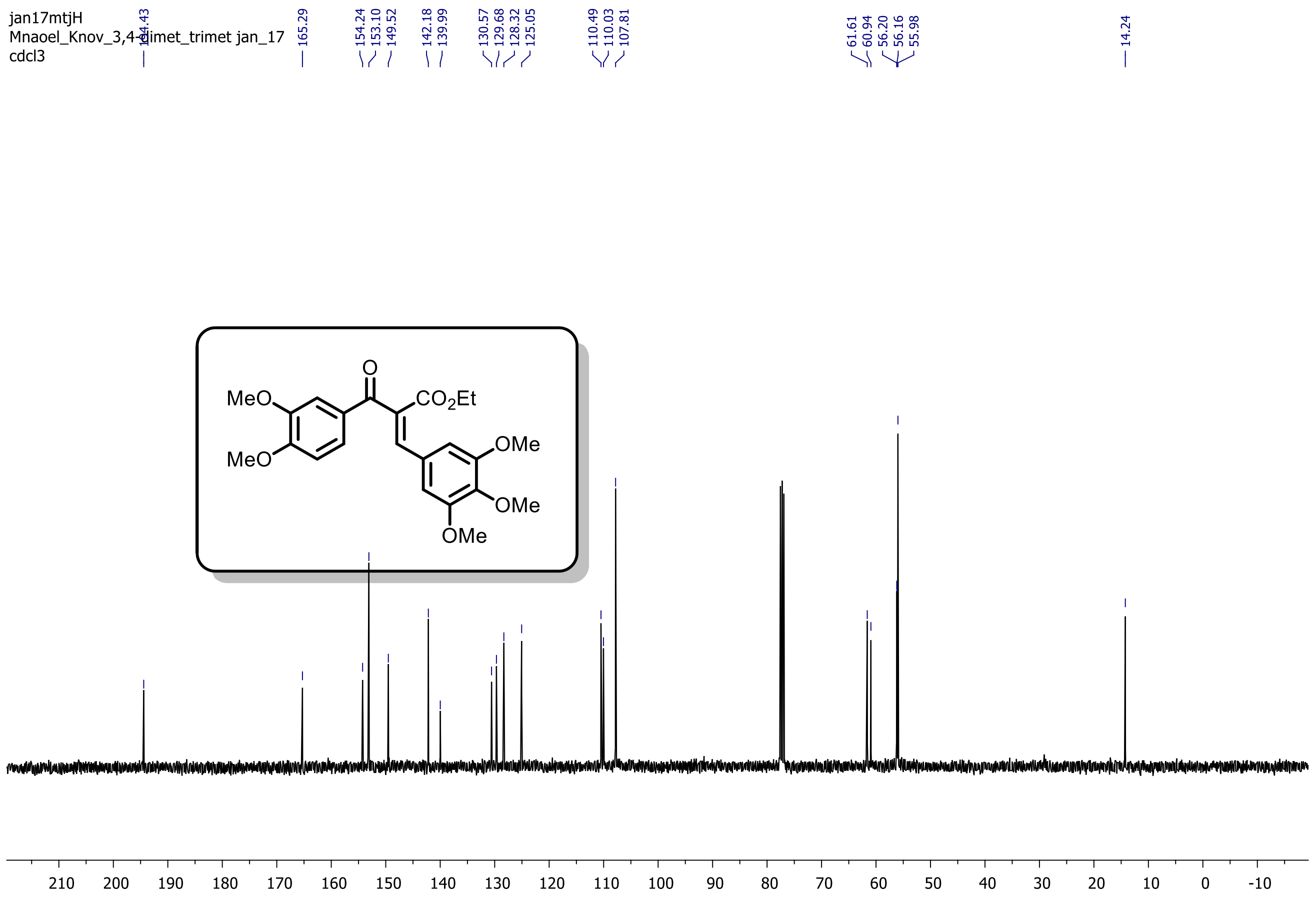

Figure S28. ^{13}C NMR spectrum (101 MHz, $CDCl_3$) of compound **9cc**.

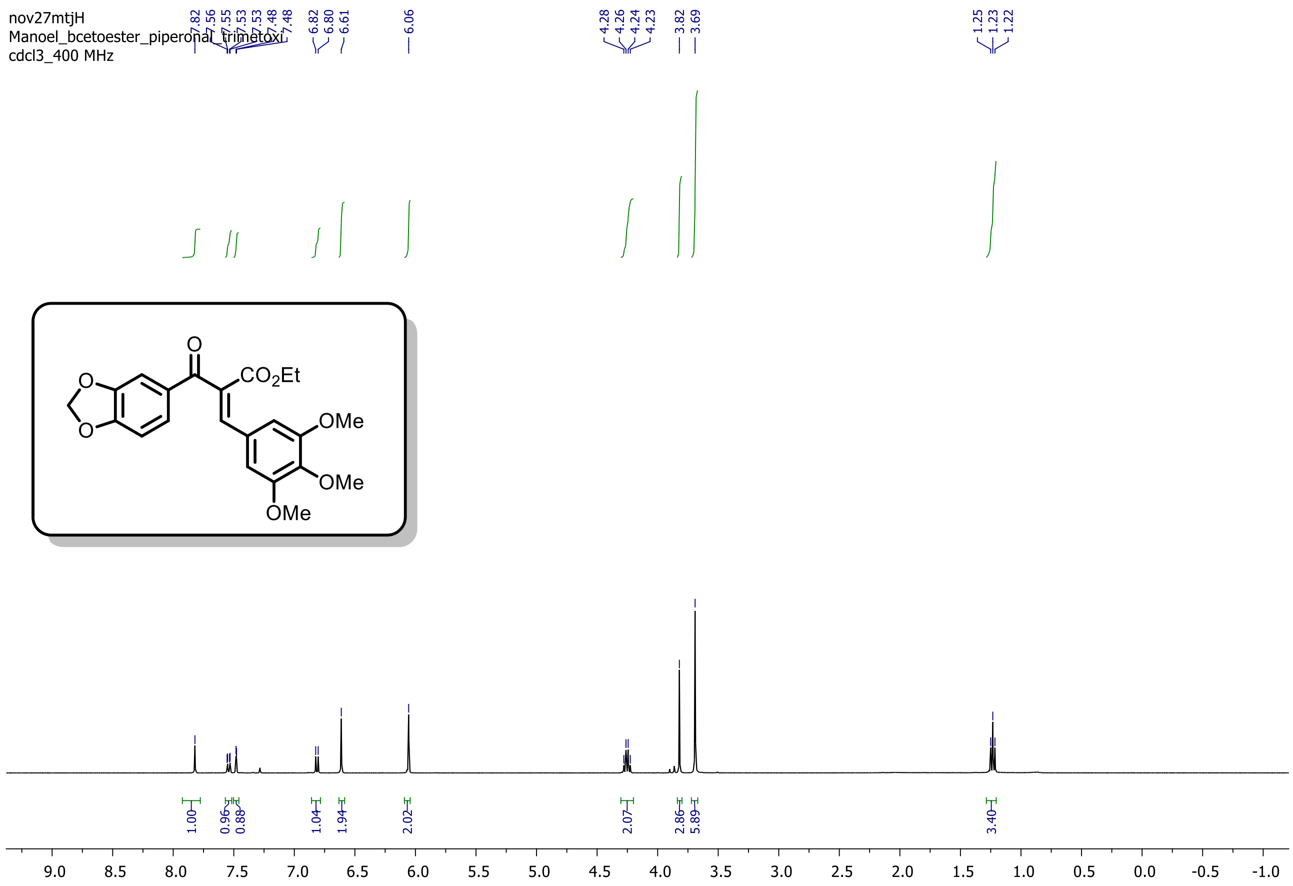

Figure S29. ^{1}H NMR spectrum (400 MHz, $CDCl_3$) of compound **9dc**.

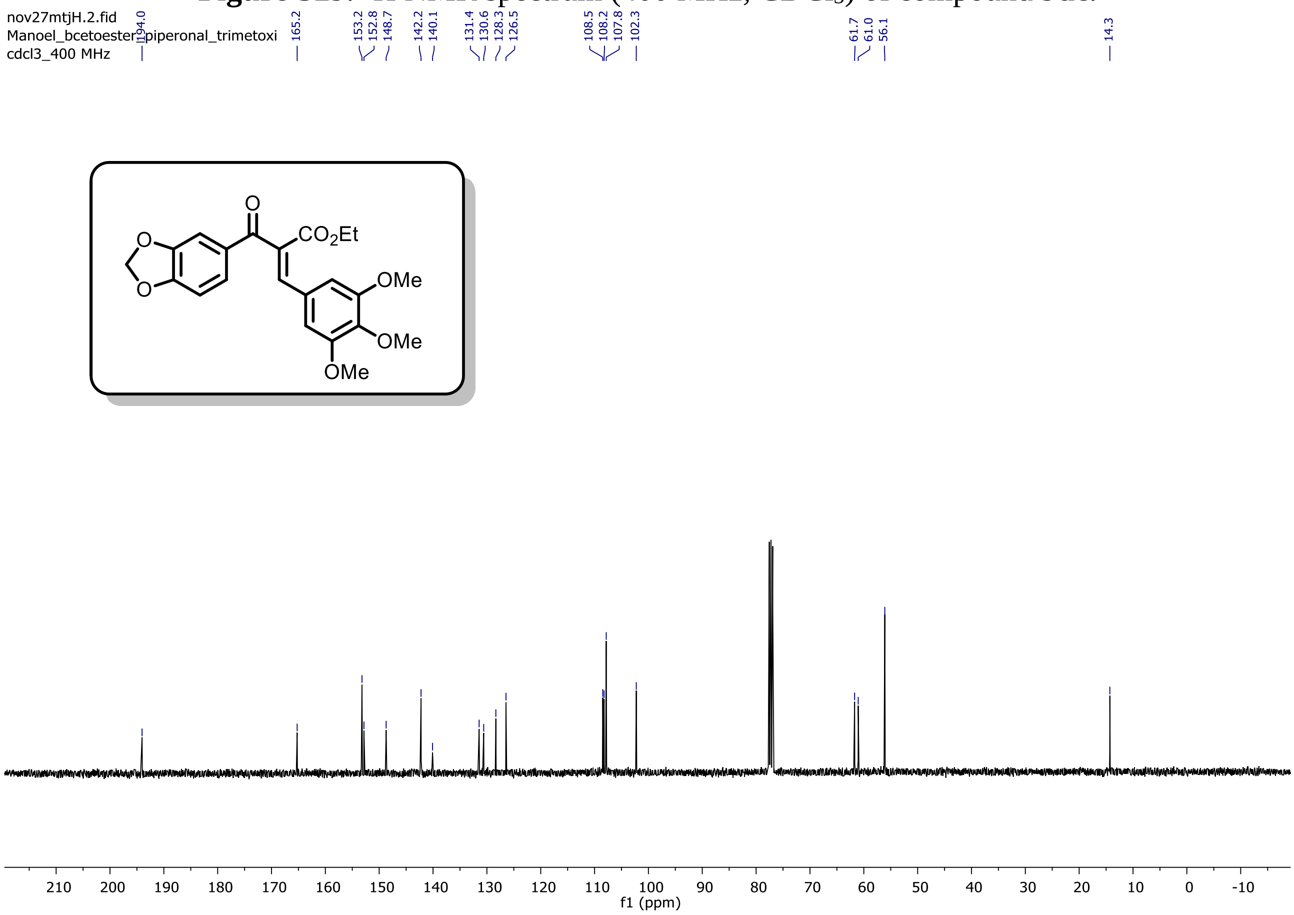

Figure S30. ^{13}C NMR spectrum (101 MHz, $CDCl_3$) of compound **9dc**.

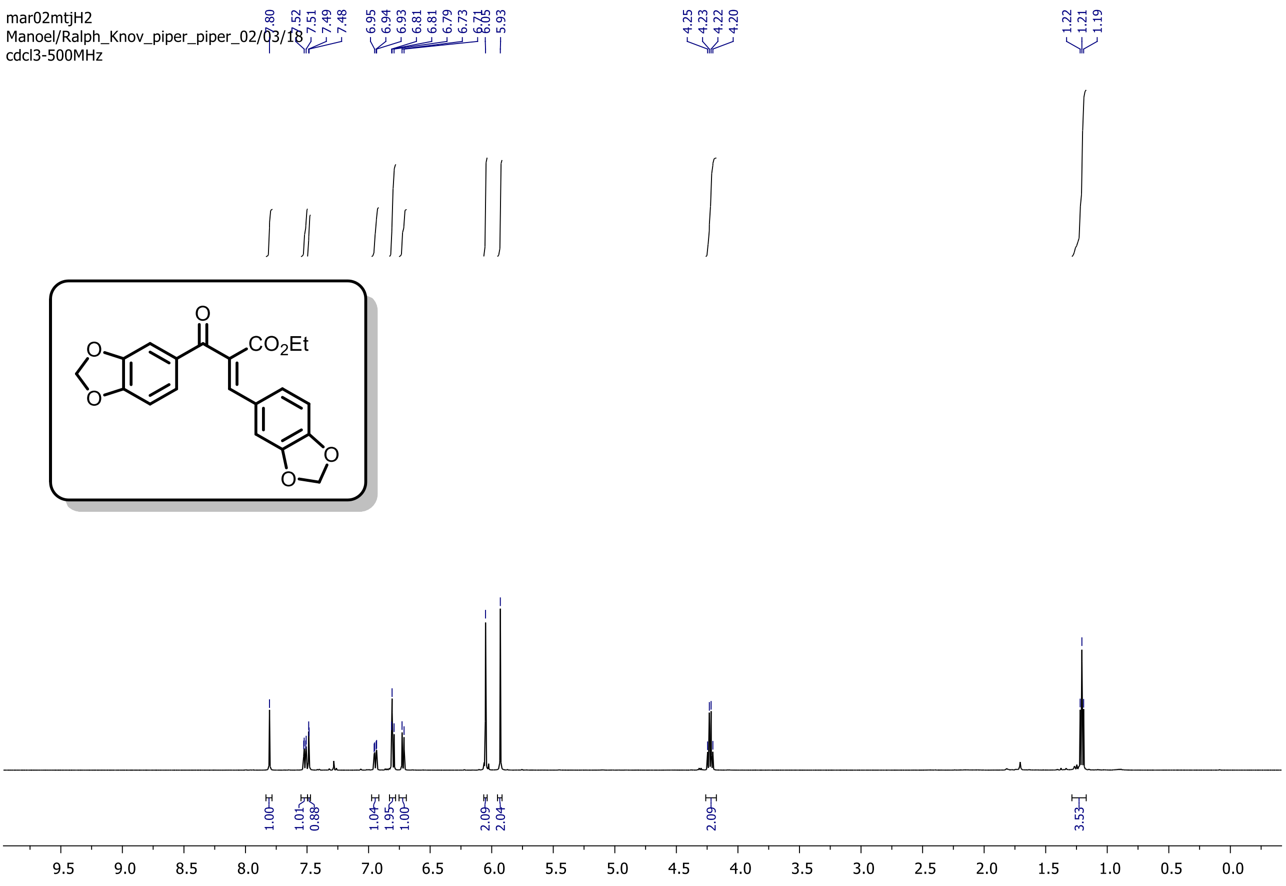

Figure S31. ^{1}H NMR spectrum (500 MHz, $CDCl_3$) of compound **9dl**.

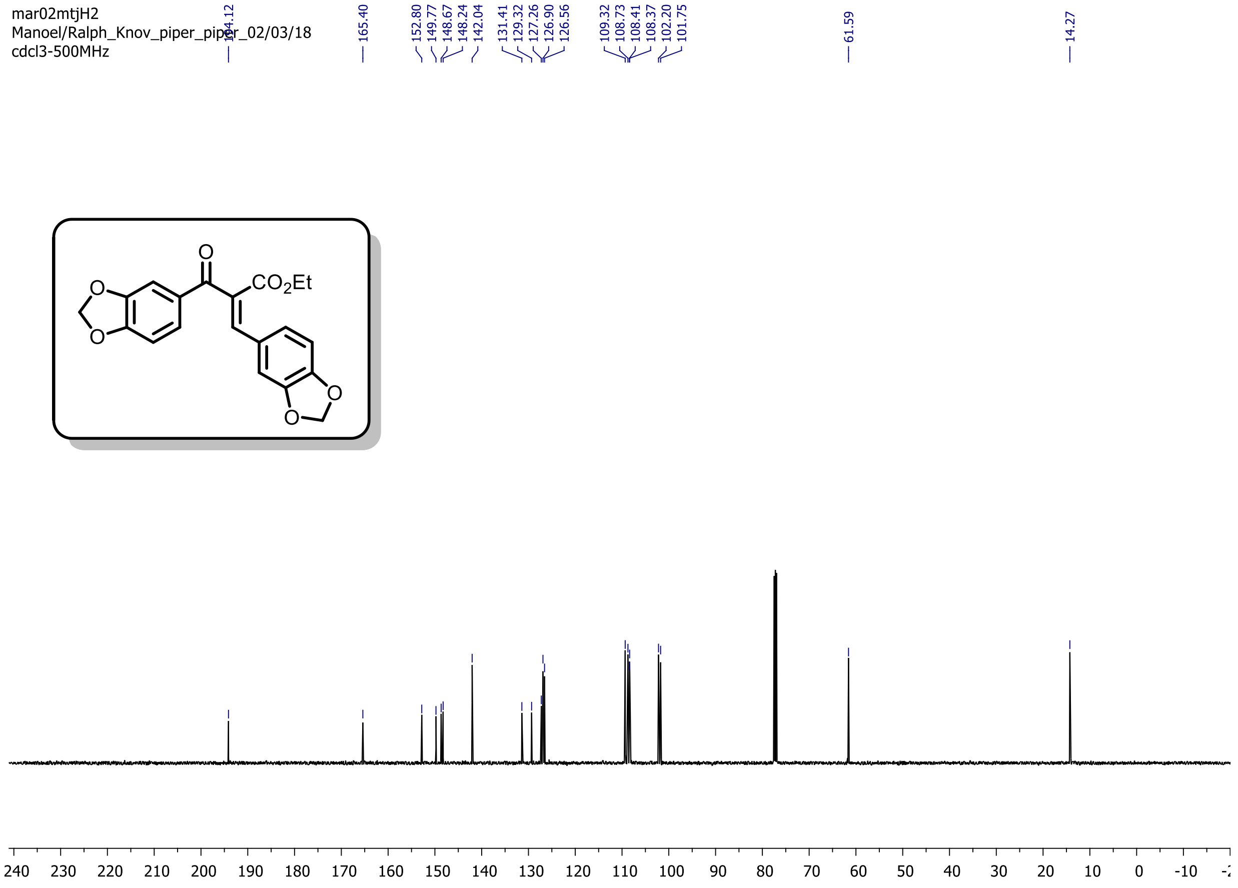

Figure S32. ^{13}C NMR spectrum (126 MHz, $CDCl_3$) of compound **9dl**.

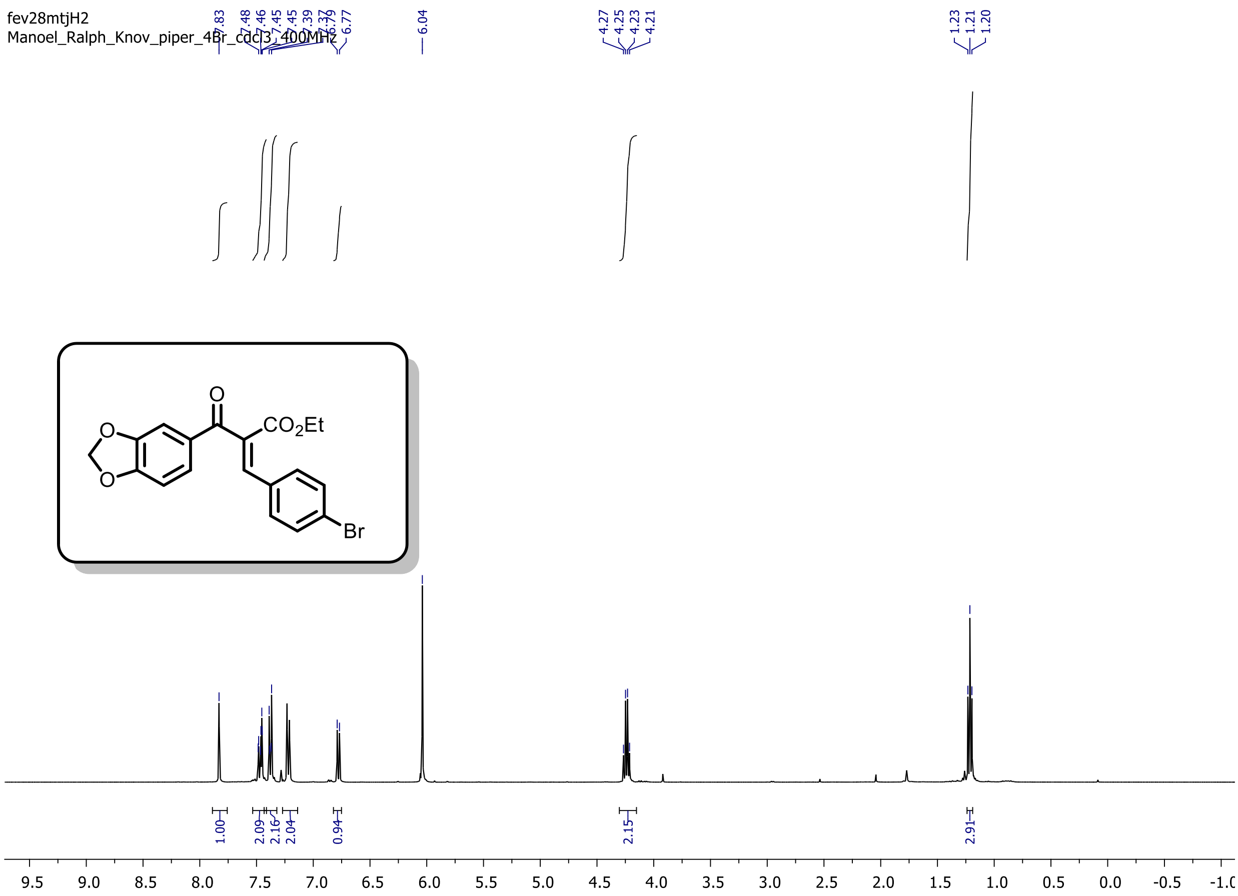

Figure S33. ^{1}H NMR spectrum (400 MHz, $CDCl_3$) of compound **9dm**.

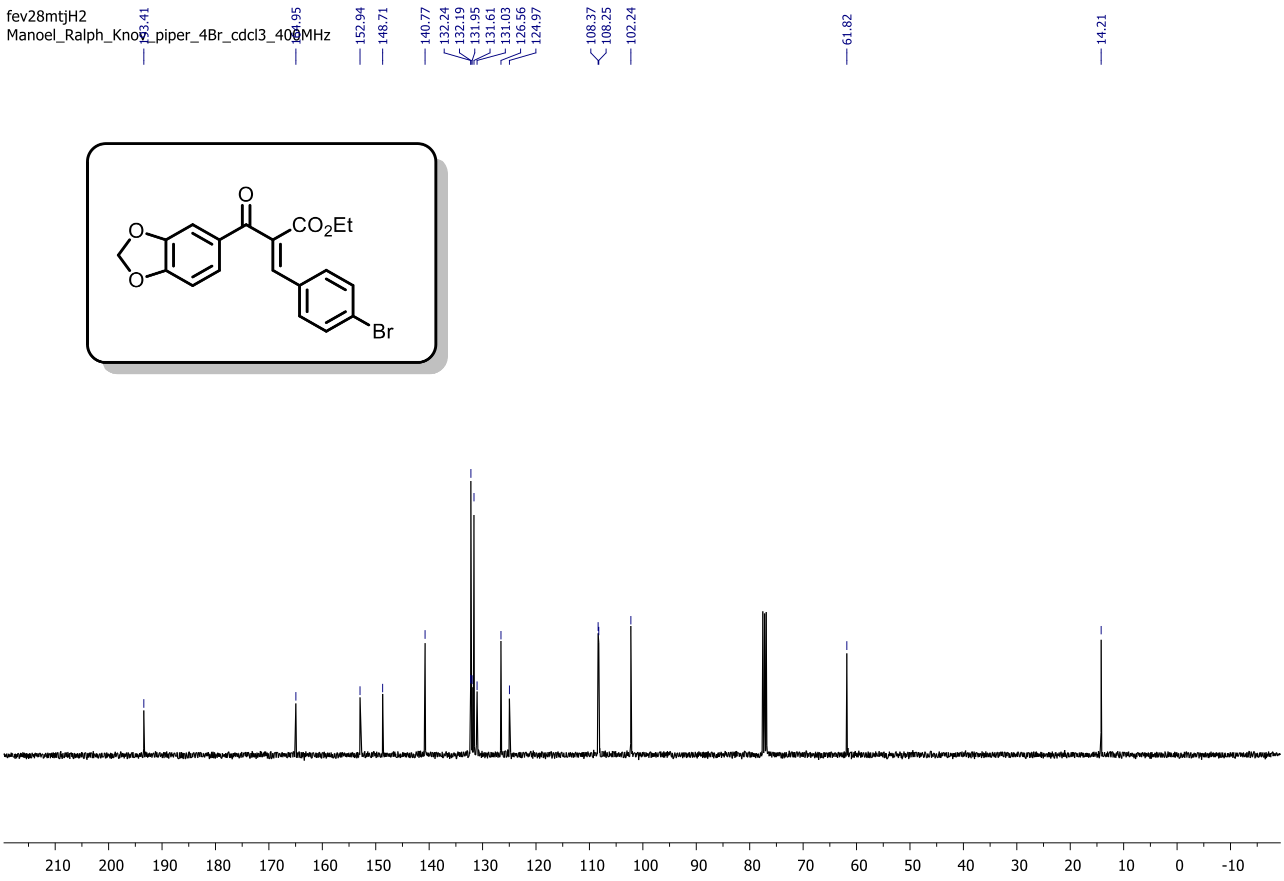

Figure S34. ^{13}C NMR spectrum (101 MHz, $CDCl_3$) of compound **9dm**.

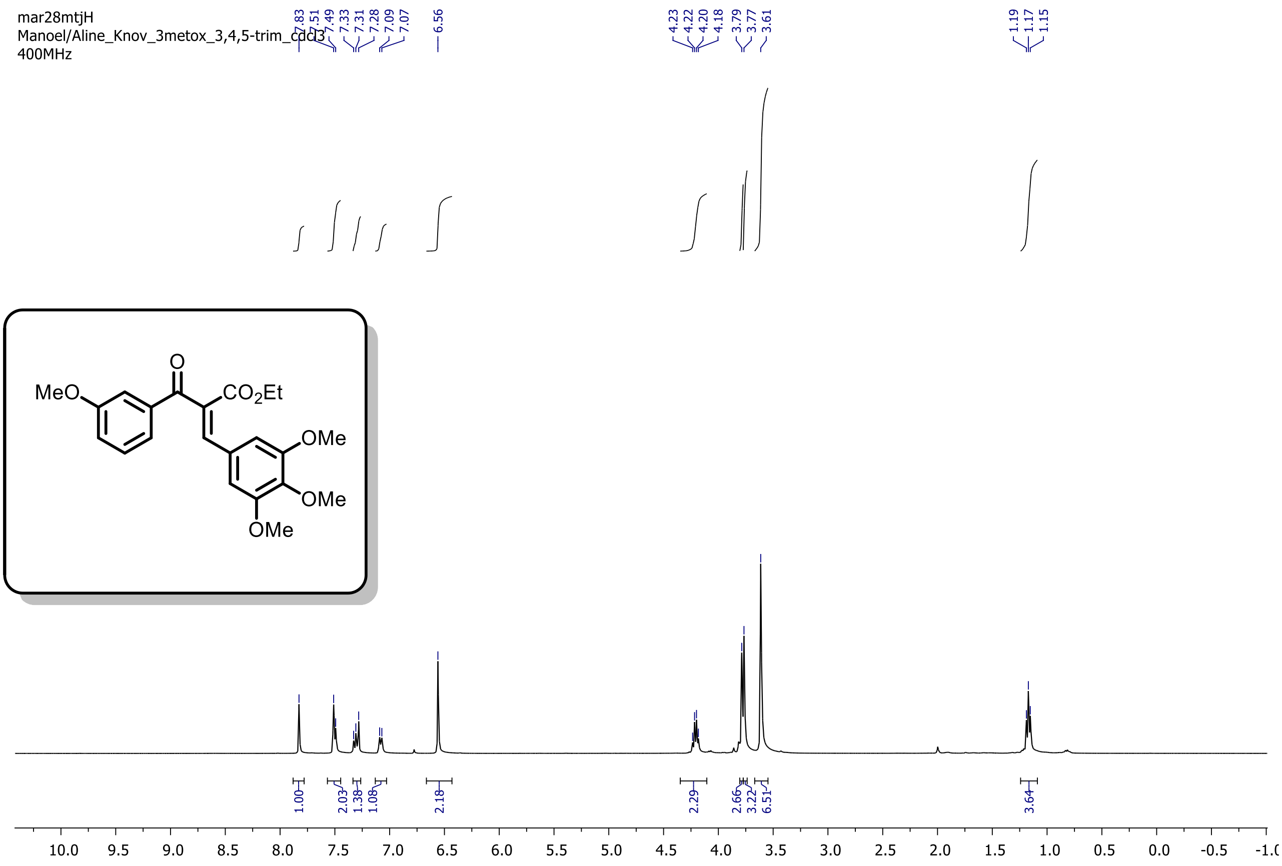

Figure S35. ^{1}H NMR spectrum (400 MHz, $CDCl_3$) of compound **9ec**.

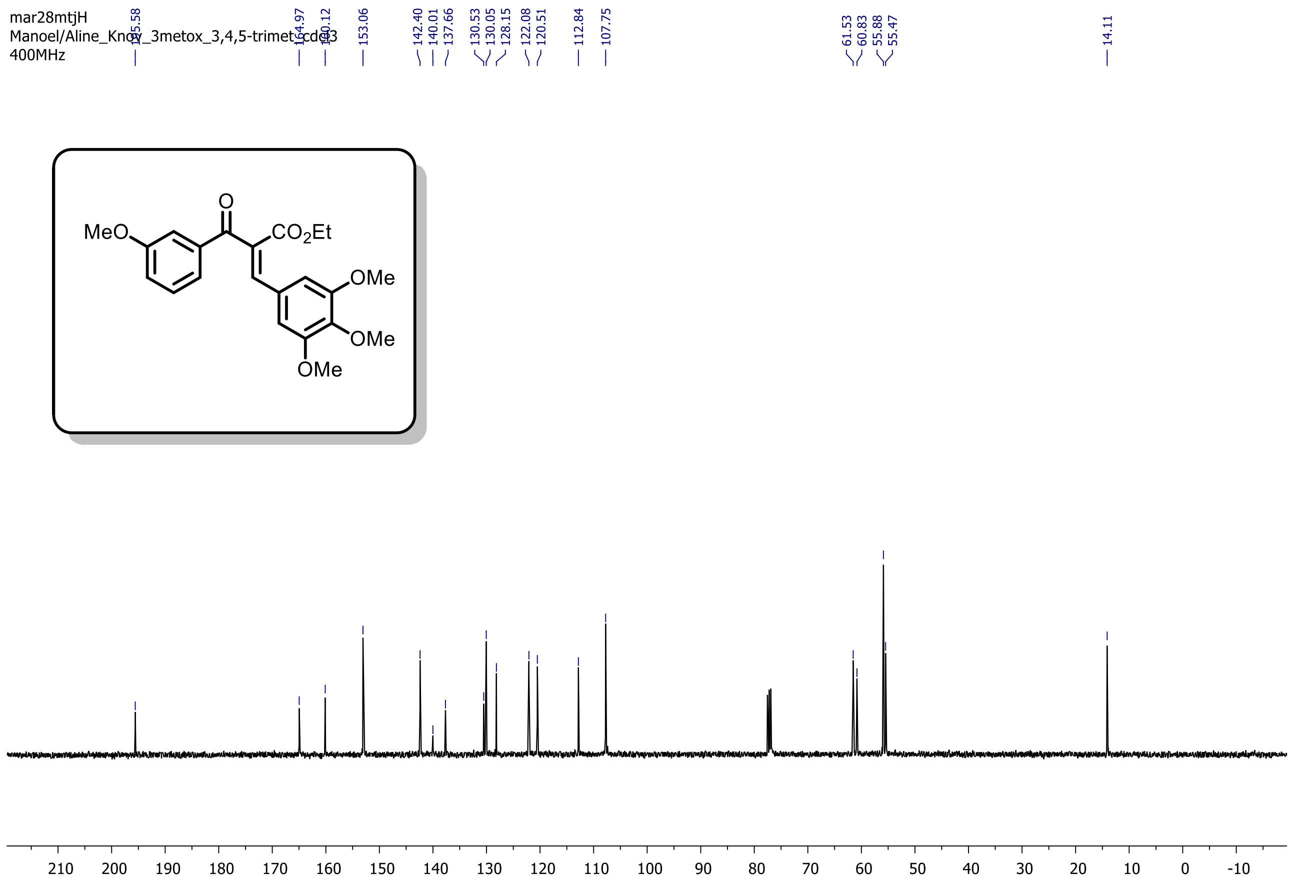

Figure S36. ^{13}C NMR spectrum (101 MHz, $CDCl_3$) of compound **9ec**.

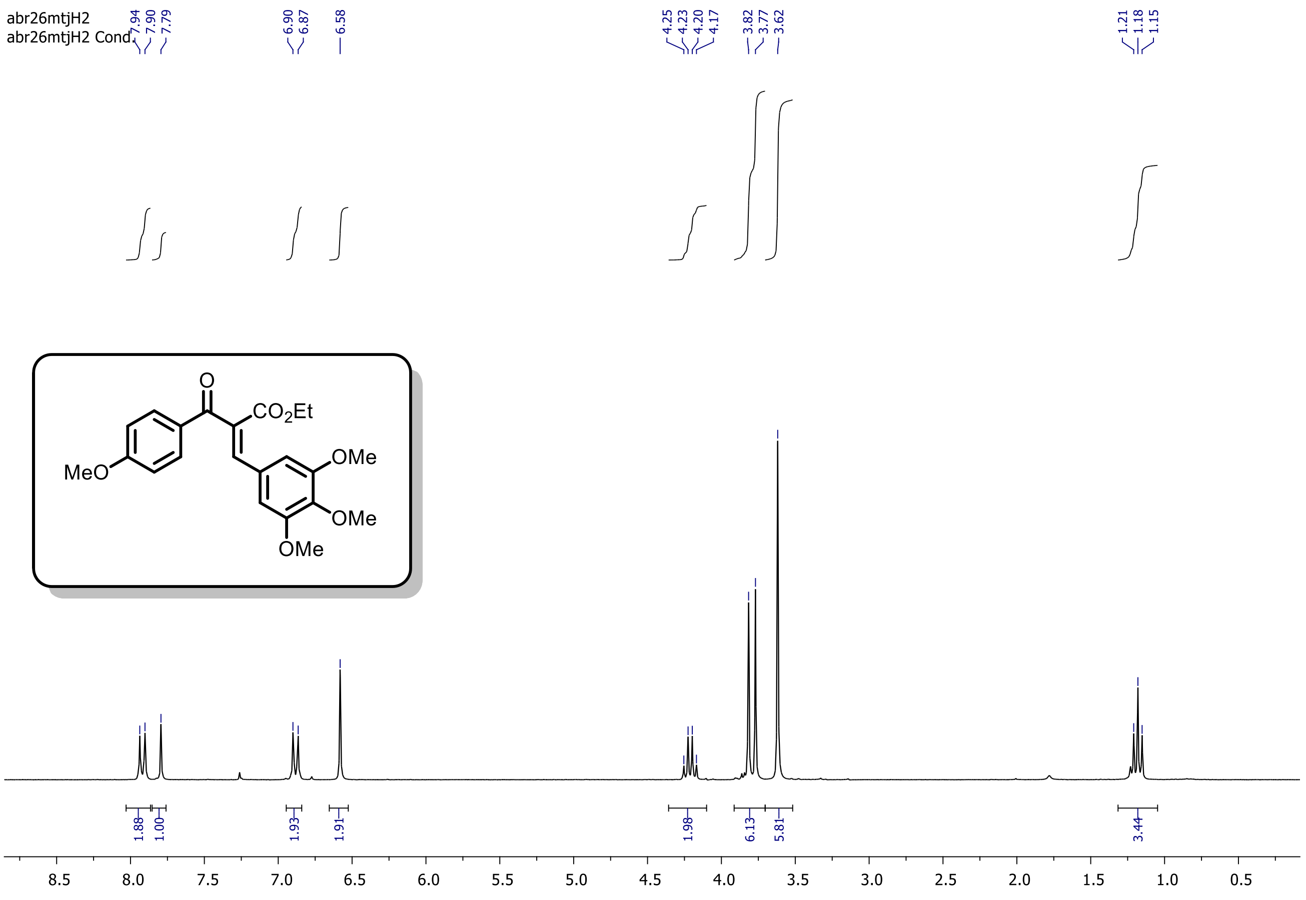

Figure S37. ^{1}H NMR spectrum (250 MHz, $CDCl_3$) of compound **9fc**.

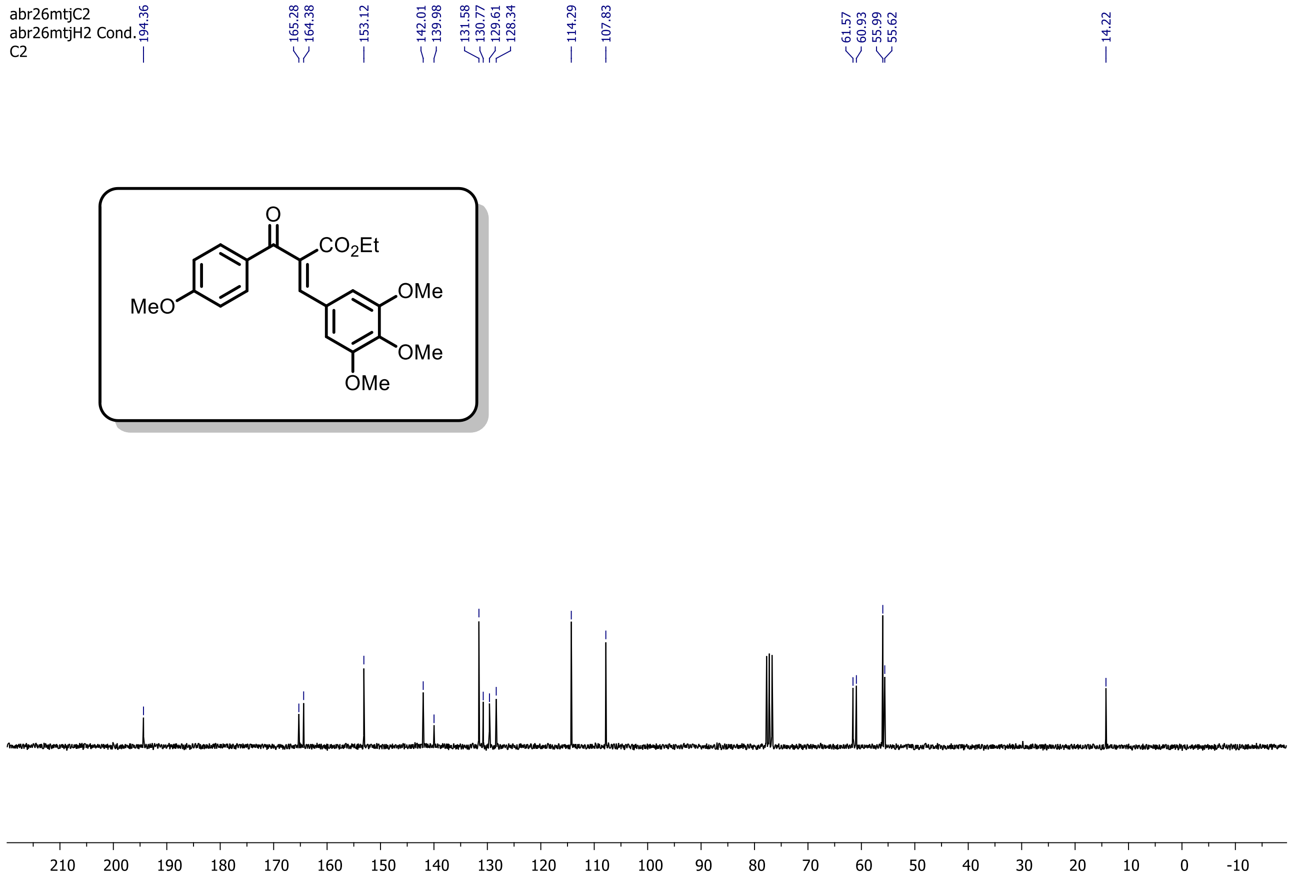

Figure S38. ^{13}C NMR spectrum (63 MHz, $CDCl_3$) of compound **9fc**.

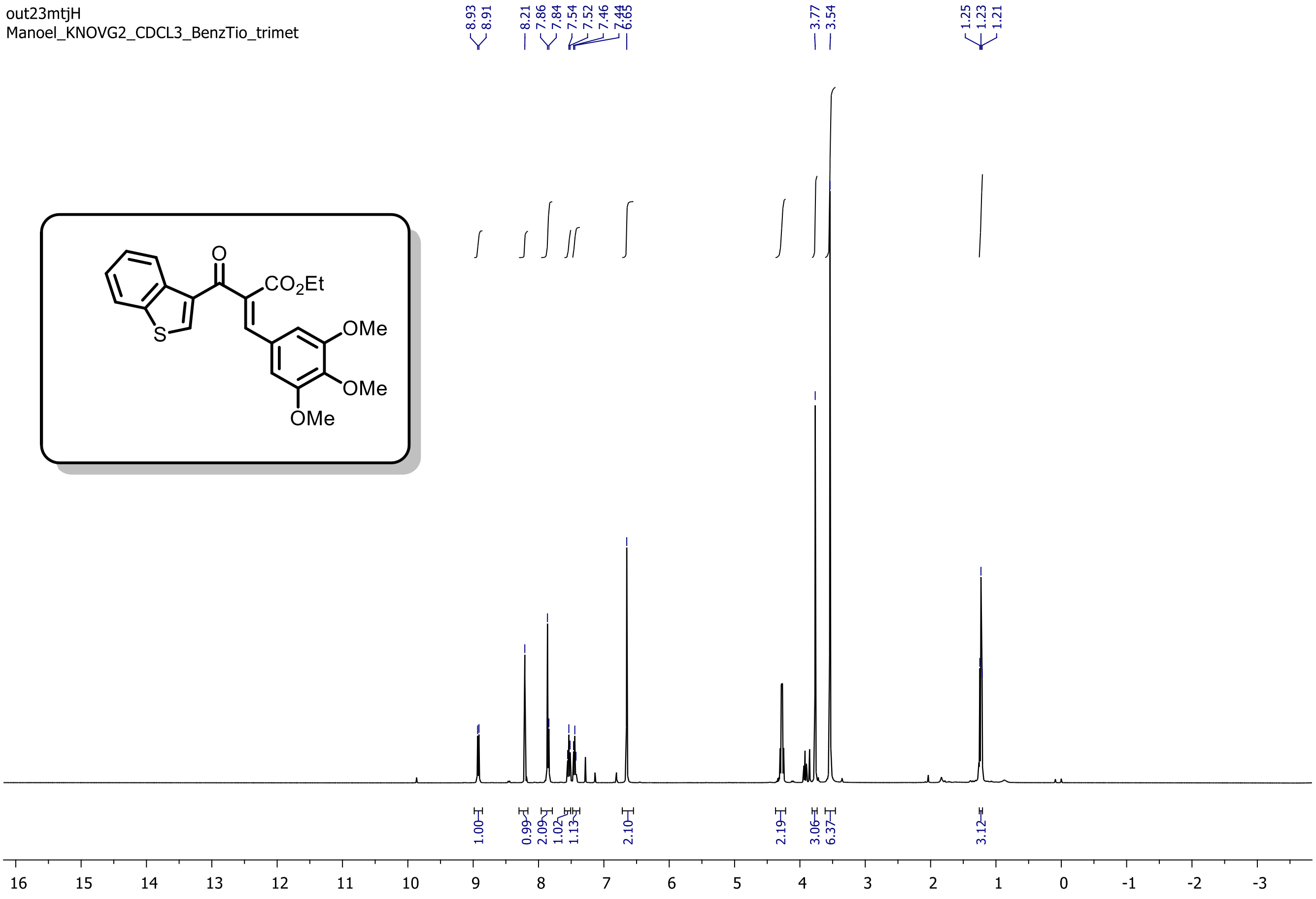

Figure S39. ^{1}H NMR spectrum (400 MHz, $CDCl_3$) of compound **9gc**.

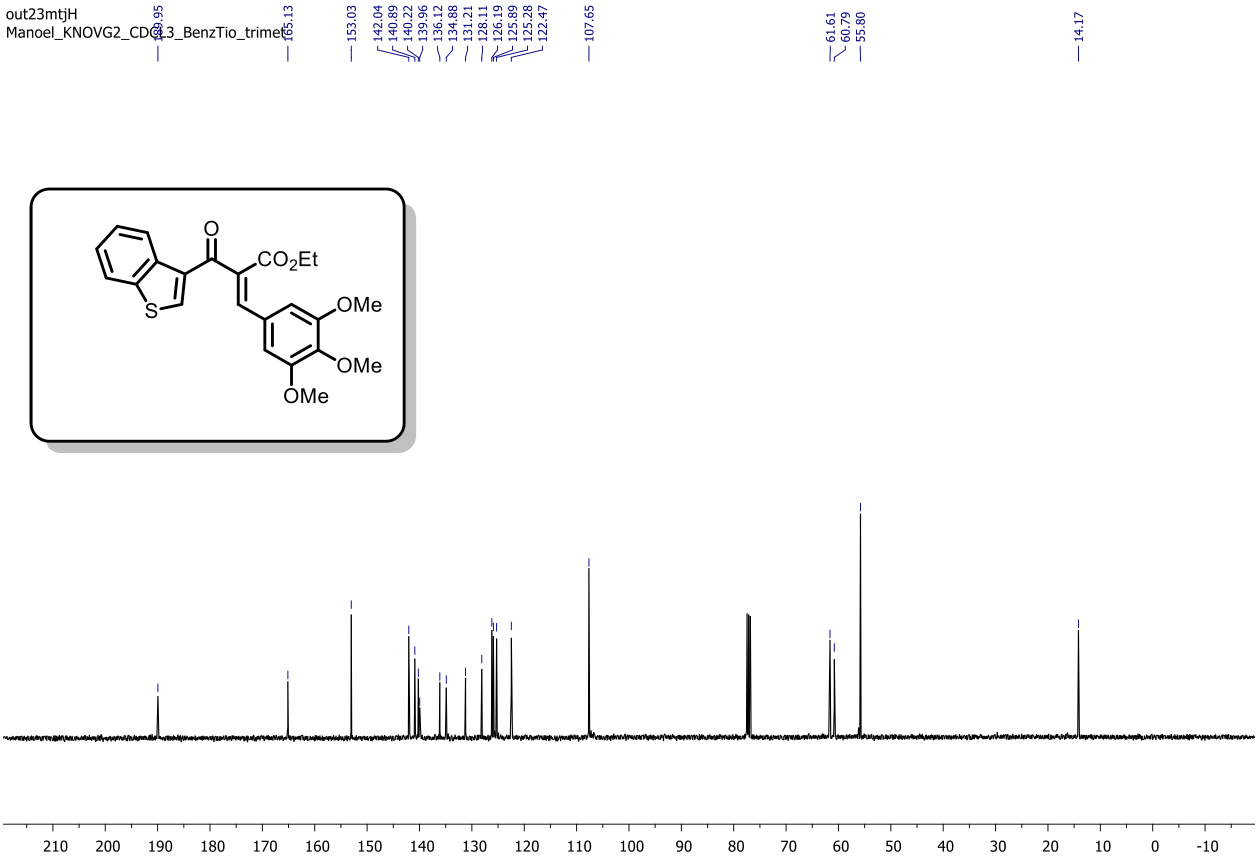

Figure S40. ^{13}C NMR spectrum (101 MHz, $CDCl_3$) of compound **9gc**.

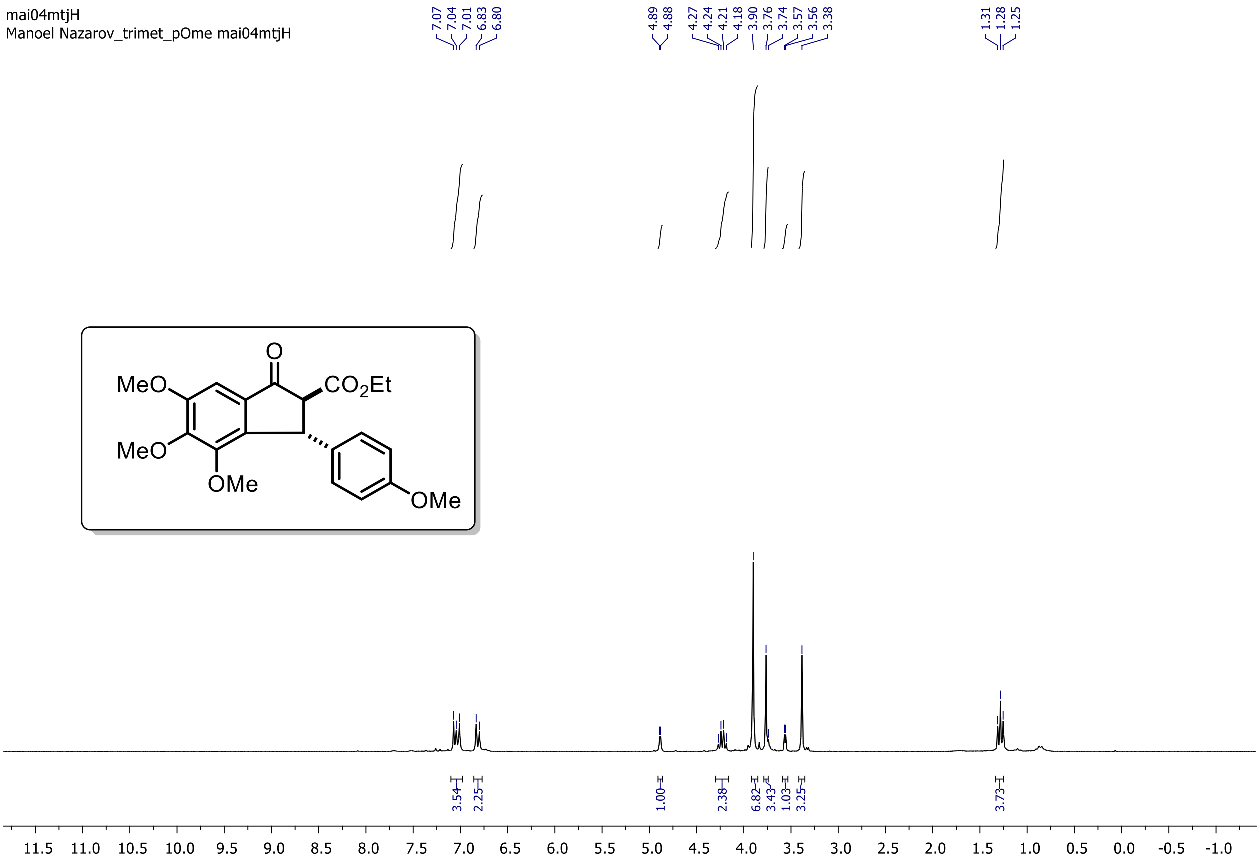

Figure S41. ^{1}H NMR spectrum (250 MHz, $CDCl_3$) of compound **10aa**.

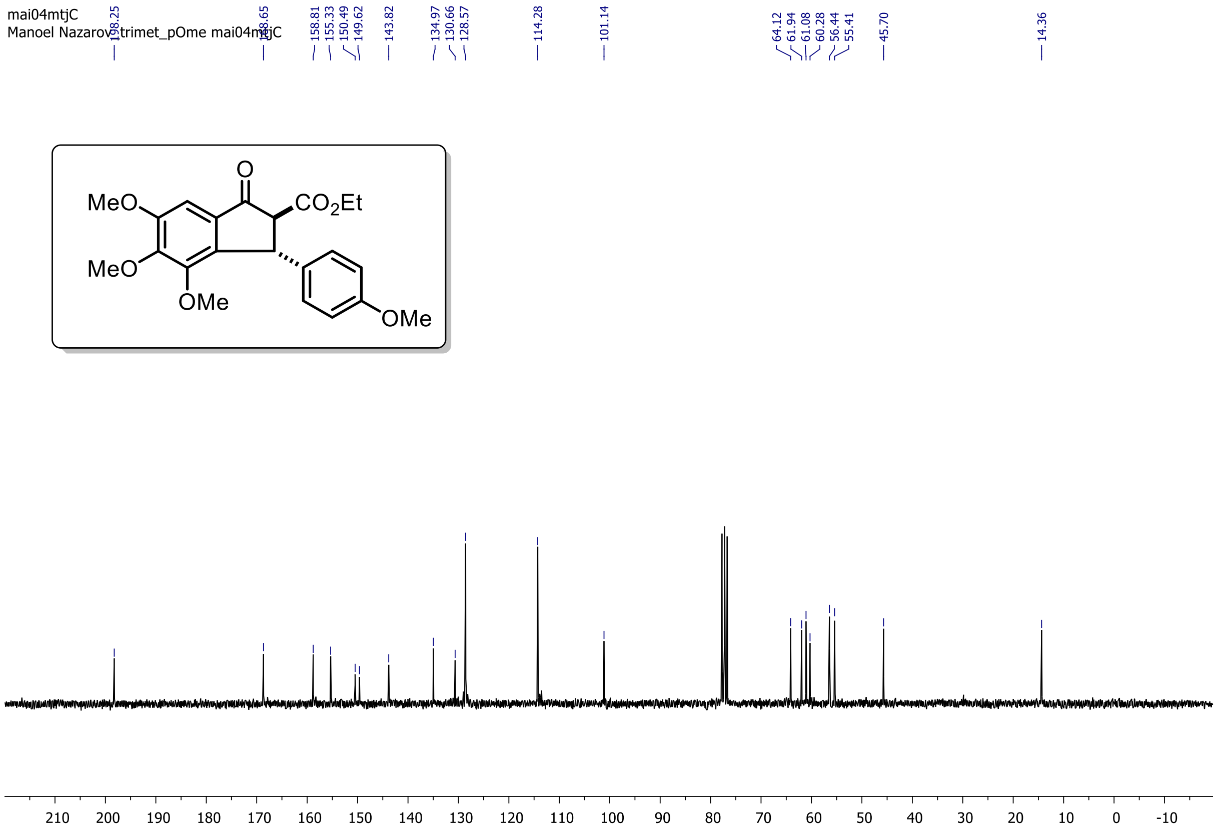

Figure S42. ^{13}C NMR spectrum (63 MHz, $CDCl_3$) of compound 10**aa**.

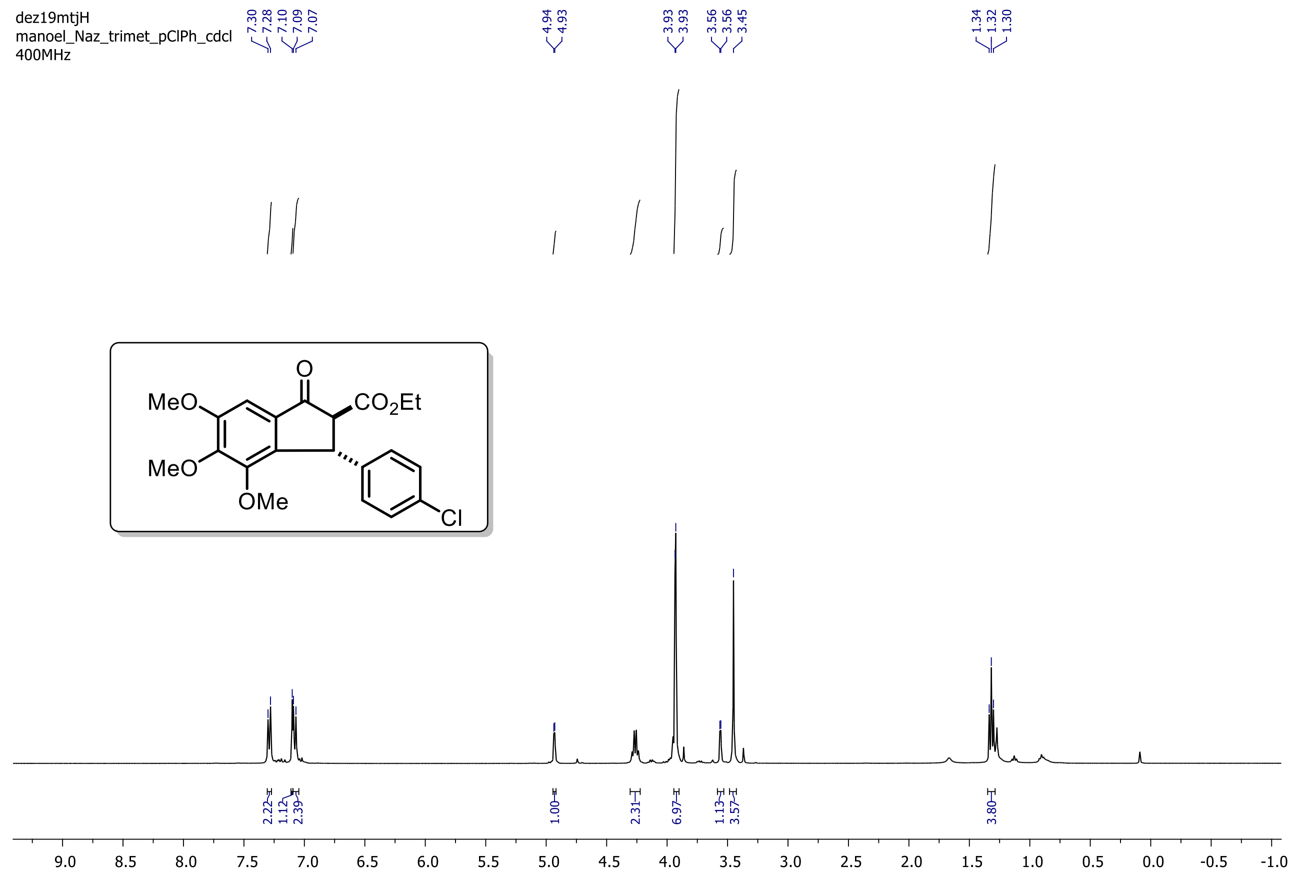

Figure S43. ^{1}H NMR spectrum (400 MHz, $CDCl_3$) of compound **10ab**.

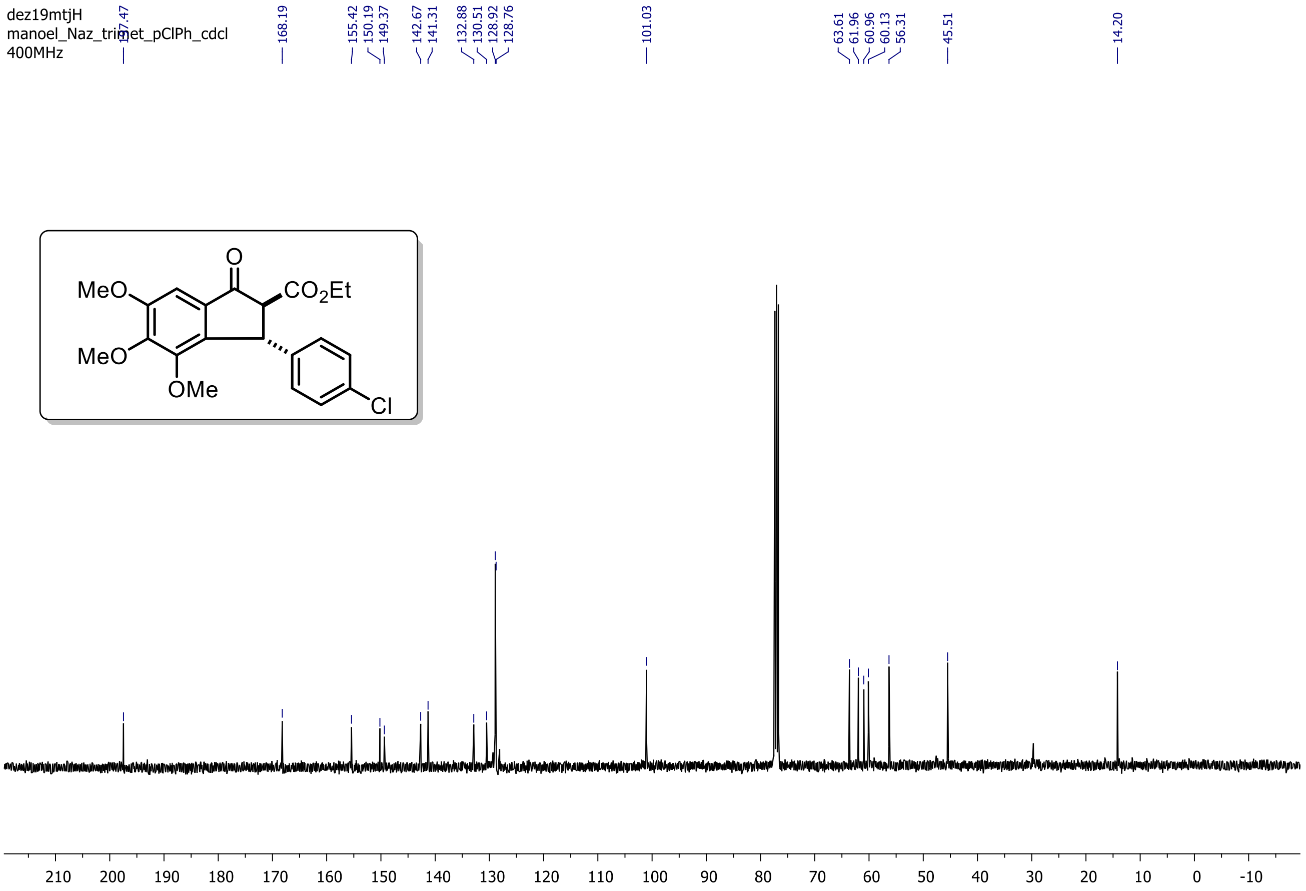

Figure S44. ^{13}C NMR spectrum (101 MHz, $CDCl_3$) of compound **10ab**.

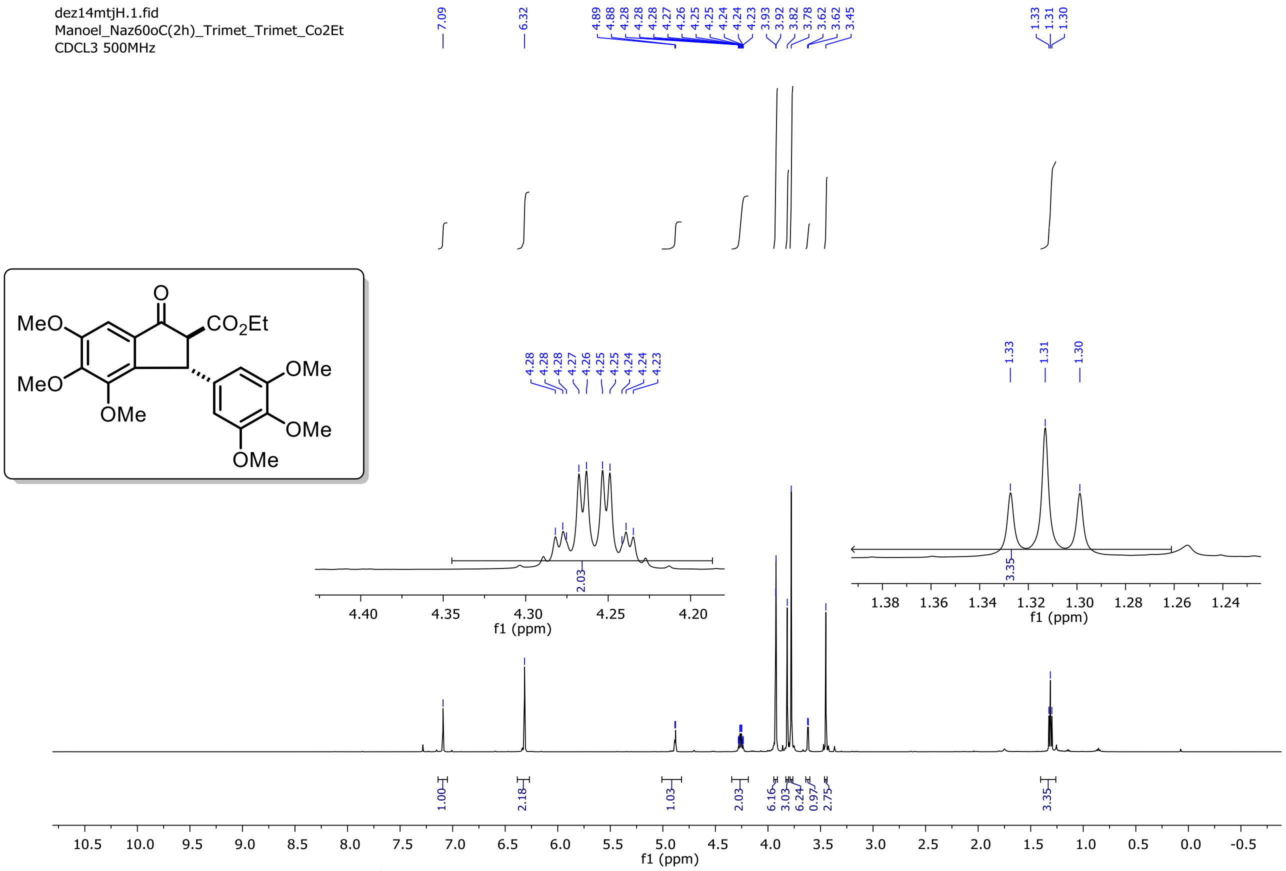

Figure S45. ^{1}H NMR spectrum (500 MHz, $CDCl_3$) of compound **10ac**.

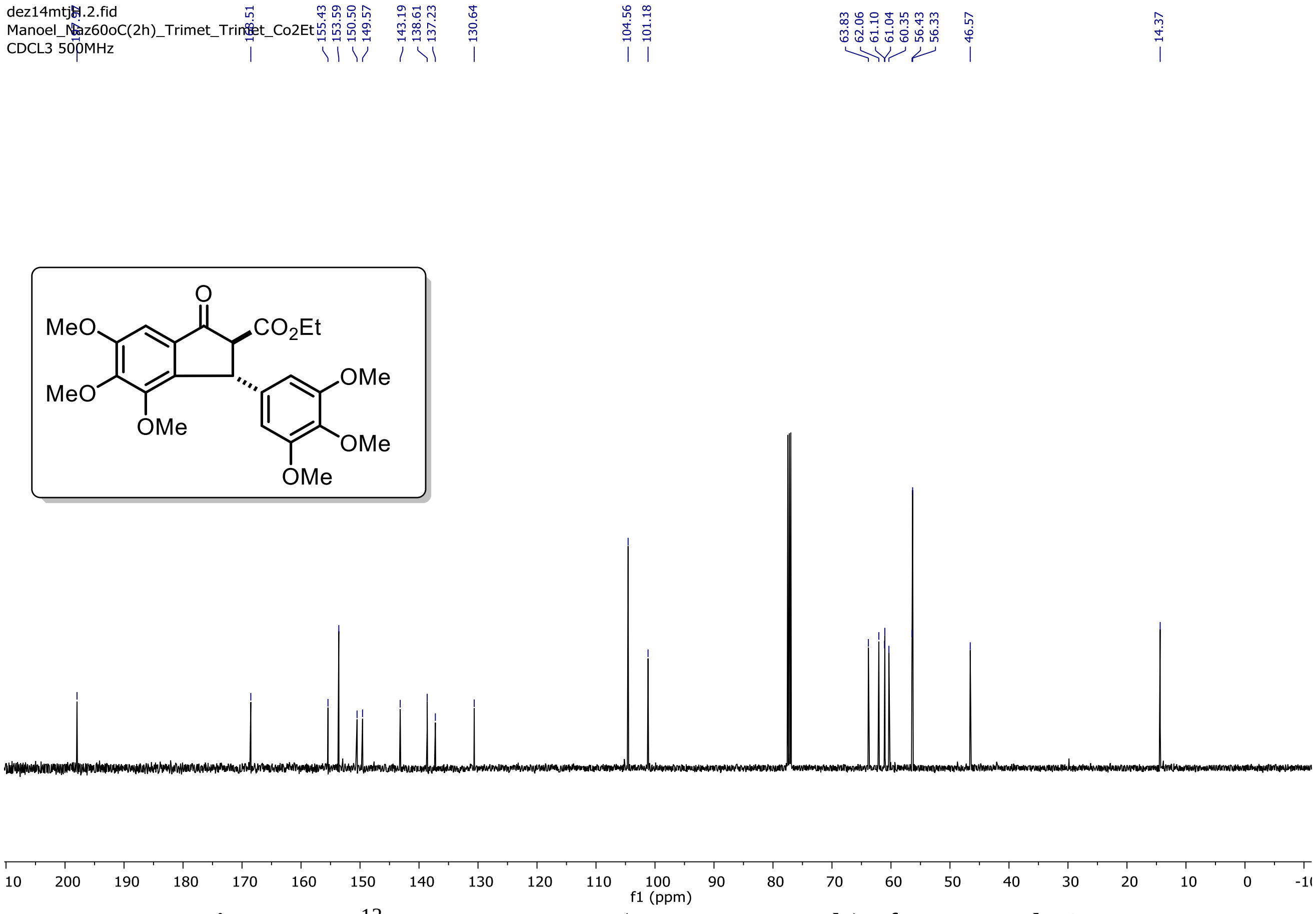

Figure S46. ^{13}C NMR spectrum (126 MHz, $CDCl_3$) of compound **10ac**.

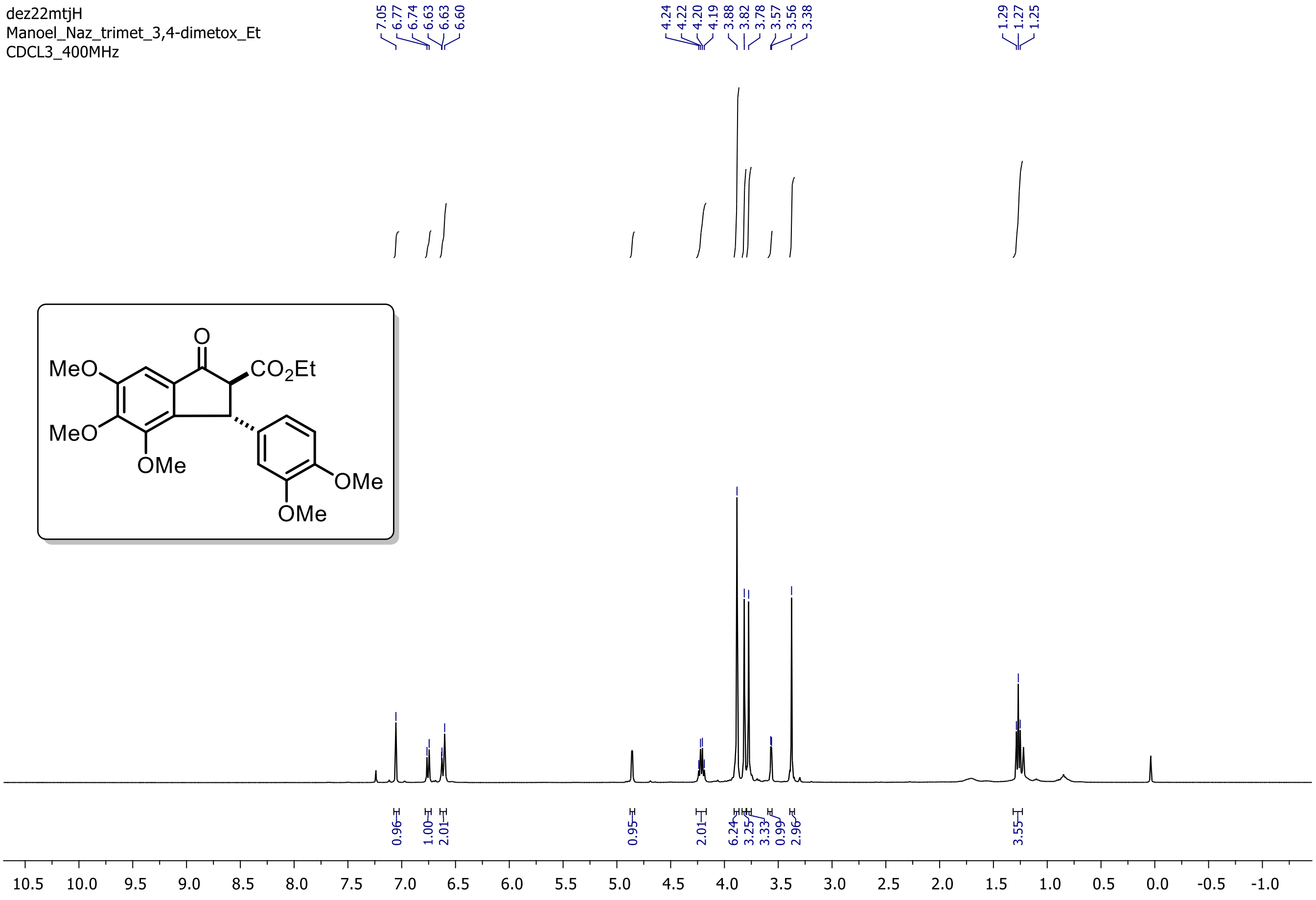

Figure S47. ^{1}H NMR spectrum (400 MHz, $CDCl_3$) of compound **10ad**.

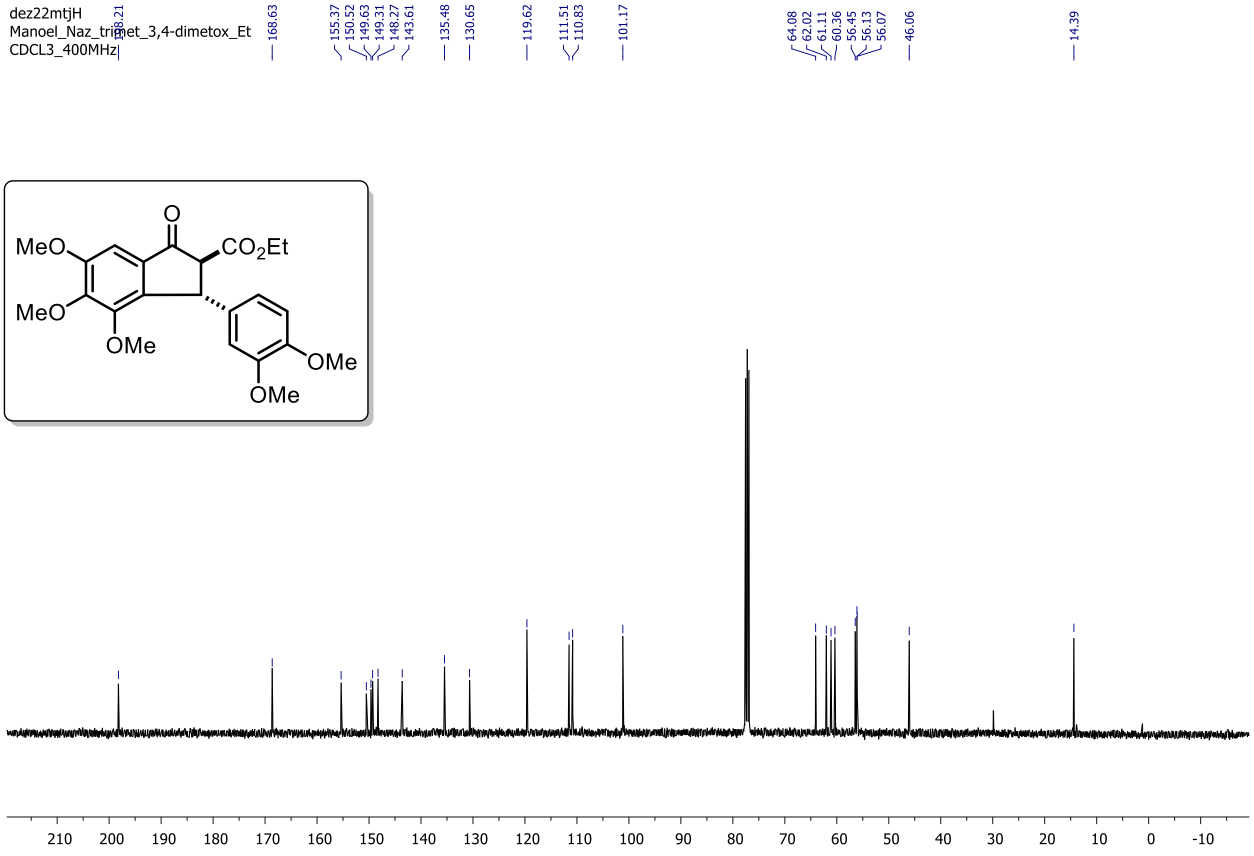

Figure S48. ^{13}C NMR spectrum (101 MHz, $CDCl_3$) of compound **10ad**.

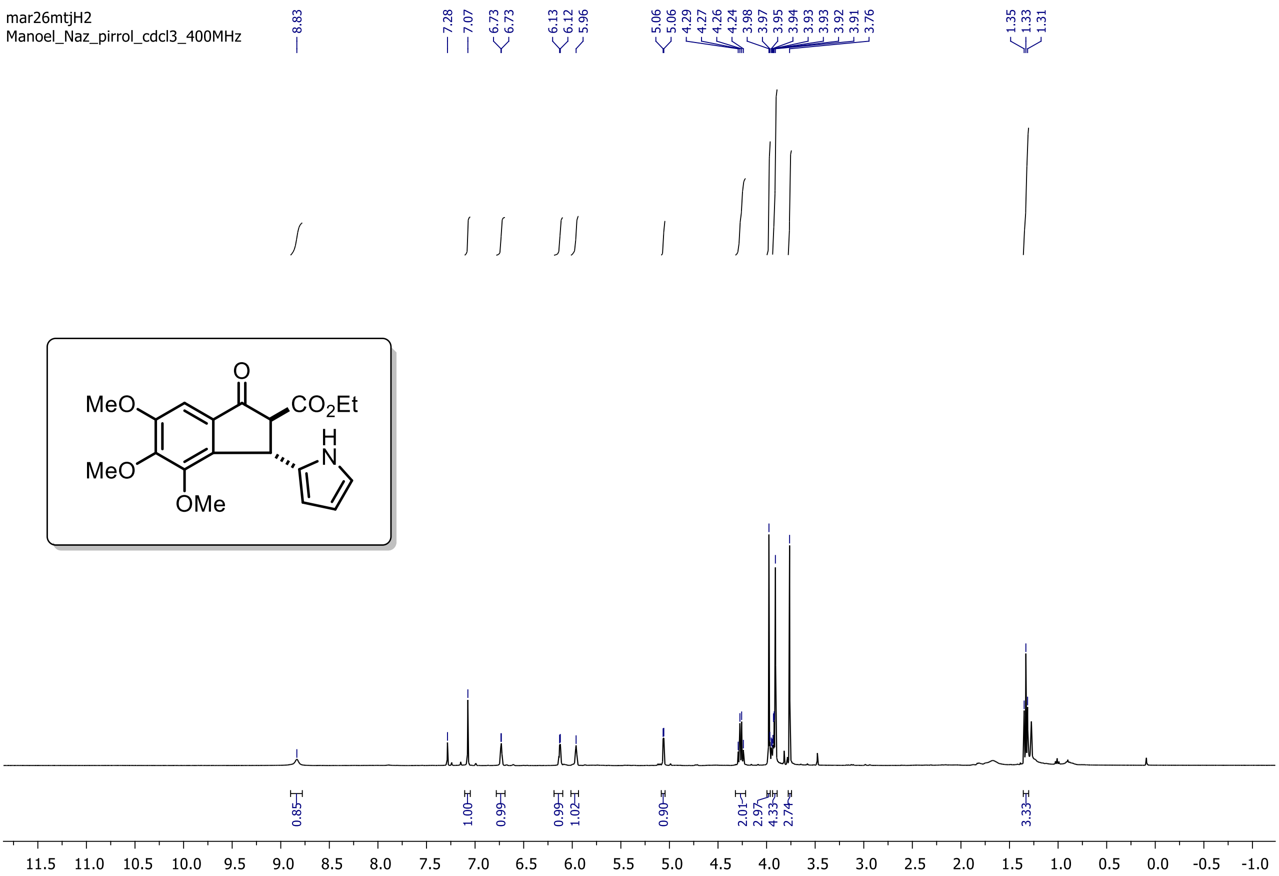

Figure S49. ^{1}H NMR spectrum (400 MHz, $CDCl_3$) of compound **10ag**.

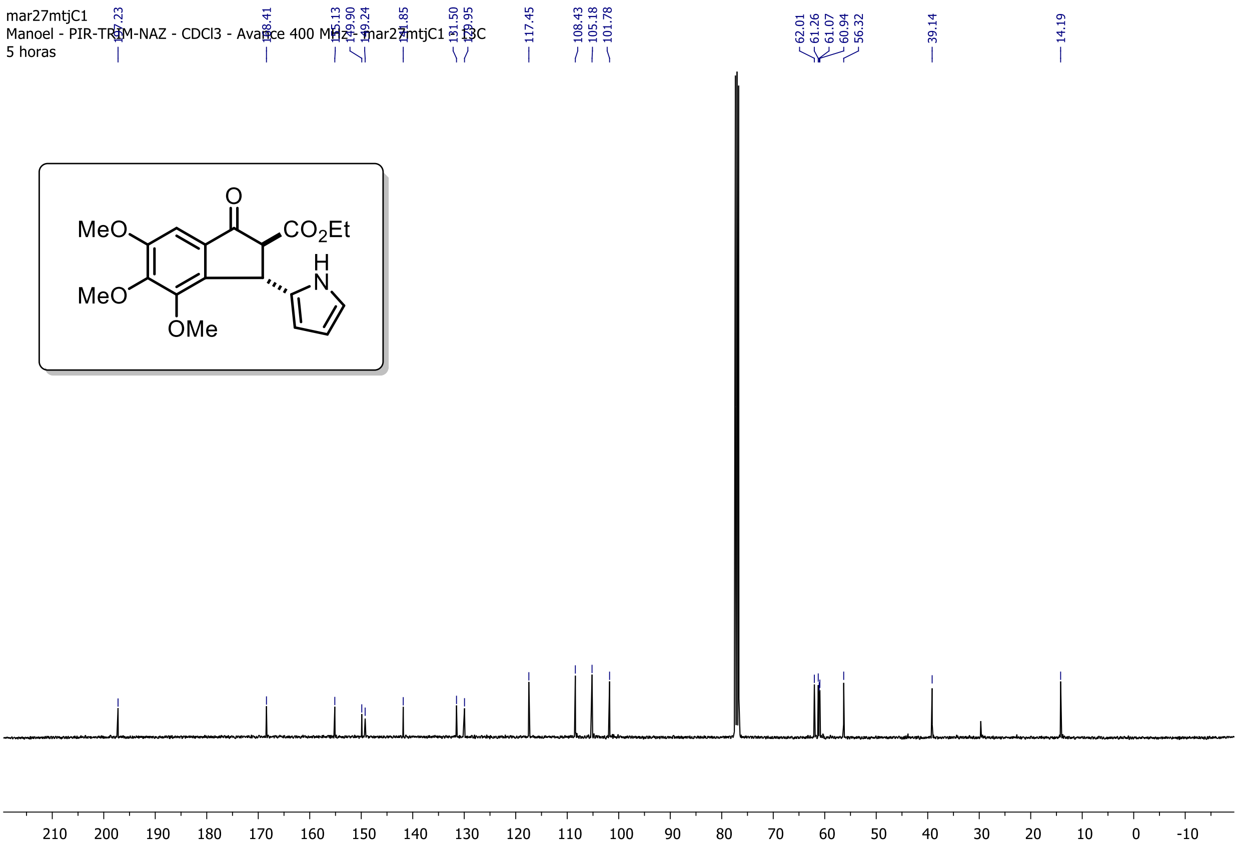

Figure S50. ^{13}C NMR spectrum (101 MHz, $CDCl_3$) of compound **10ag**.

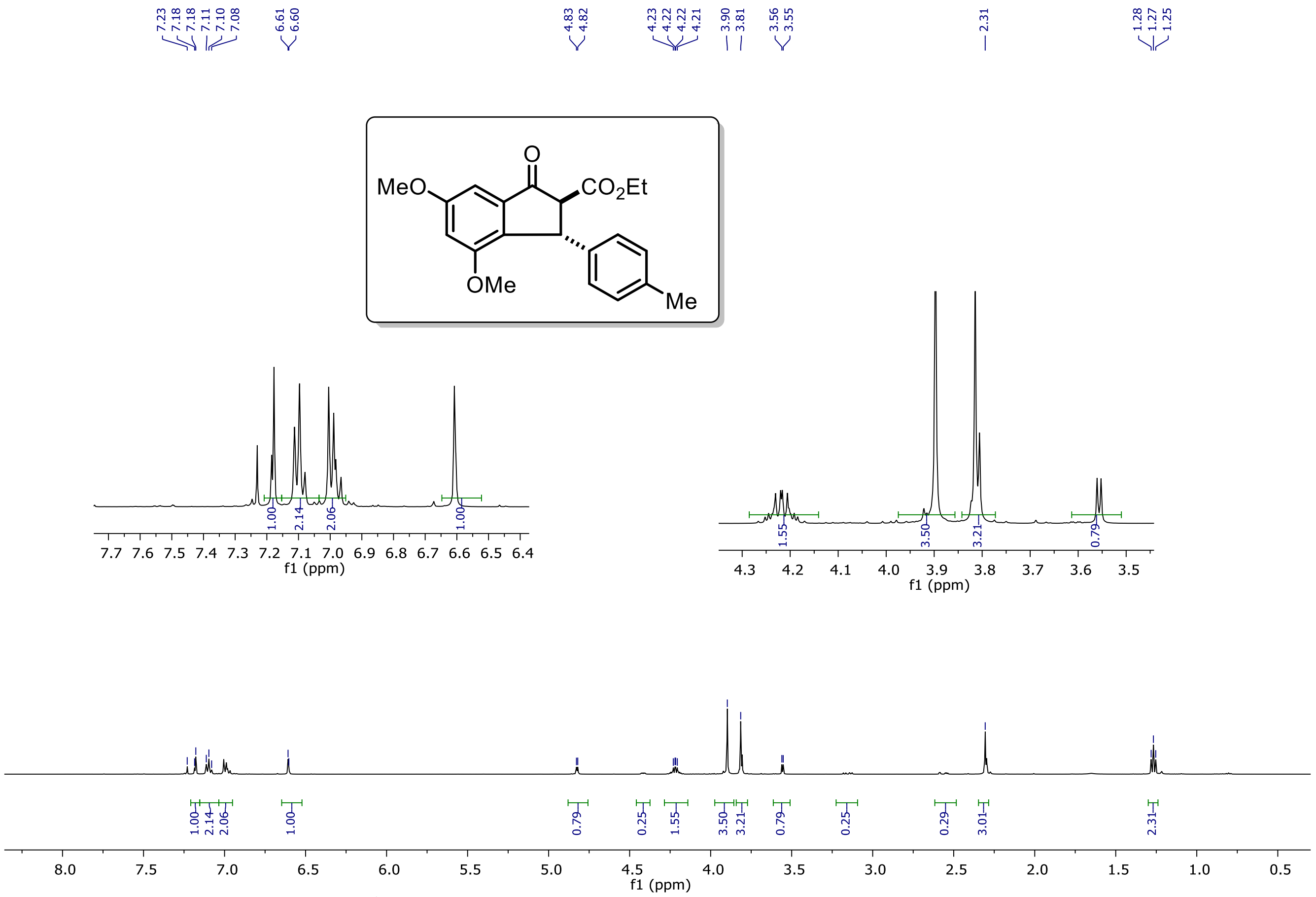

Figure S51. ^{1}H NMR spectrum (500 MHz, $CDCl_3$) of compound **10bj**.

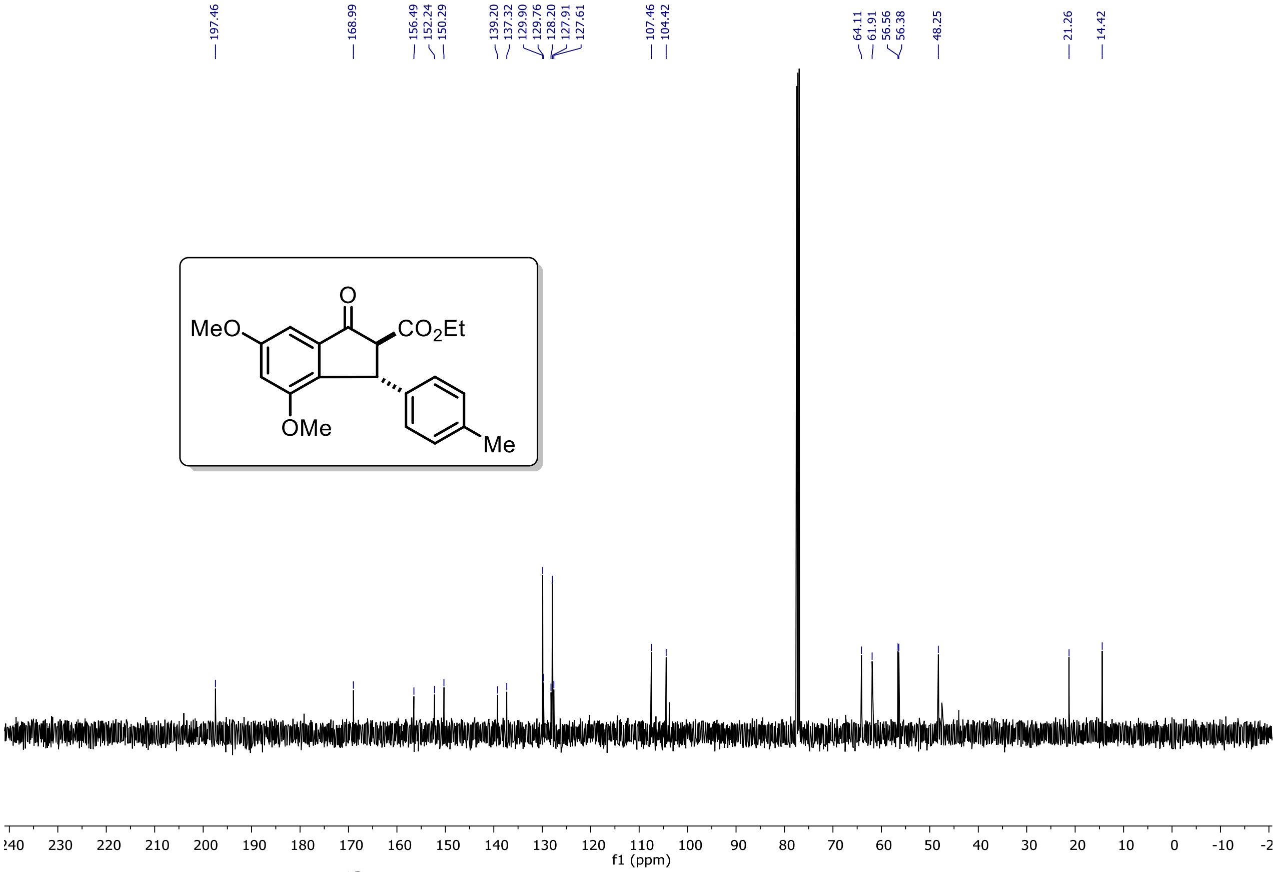

Figure S52. ^{13}C NMR spectrum (126 MHz, $CDCl_3$) of compound **10bj**.

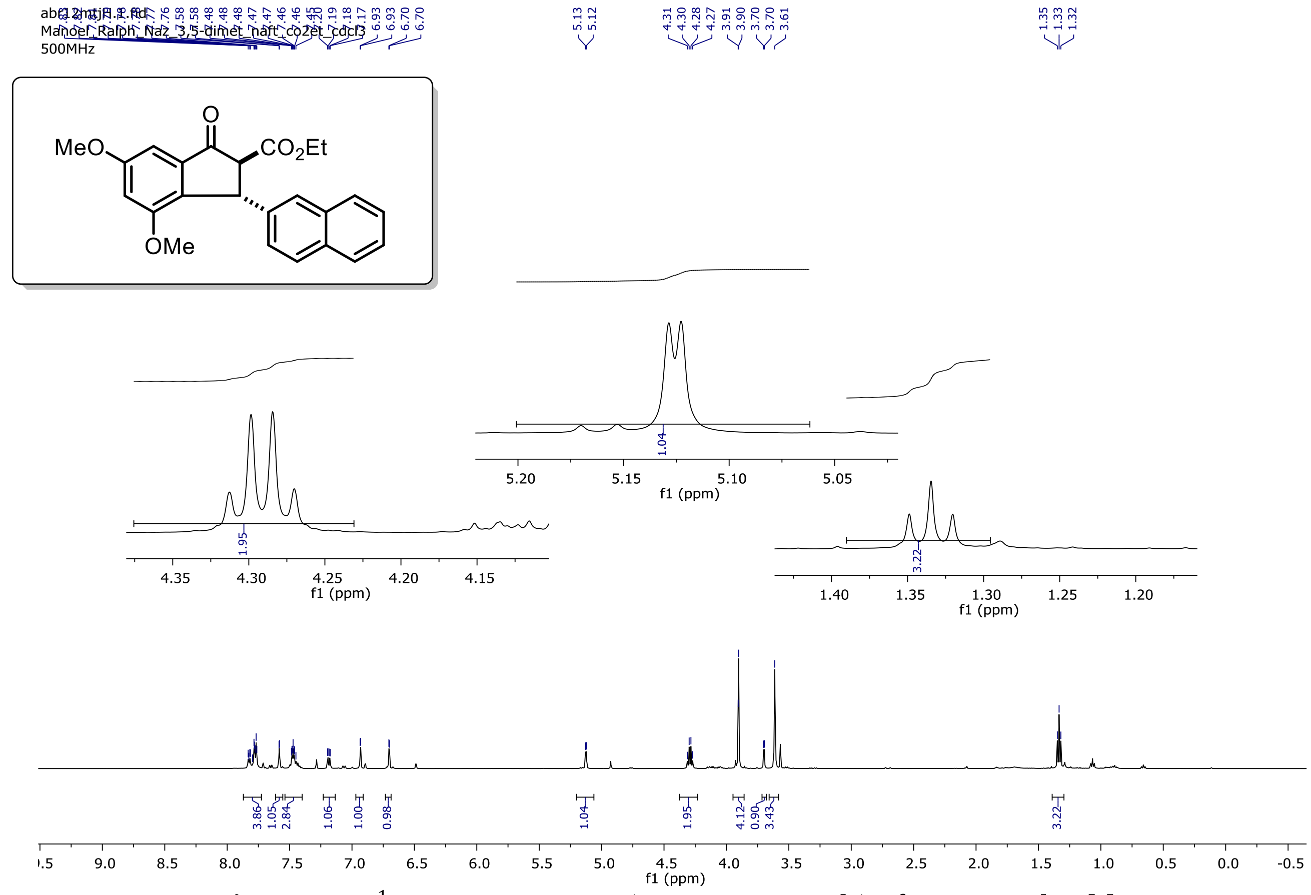

Figure S53. ^{1}H NMR spectrum (500 MHz, $CDCl_3$) of compound **10bk**.

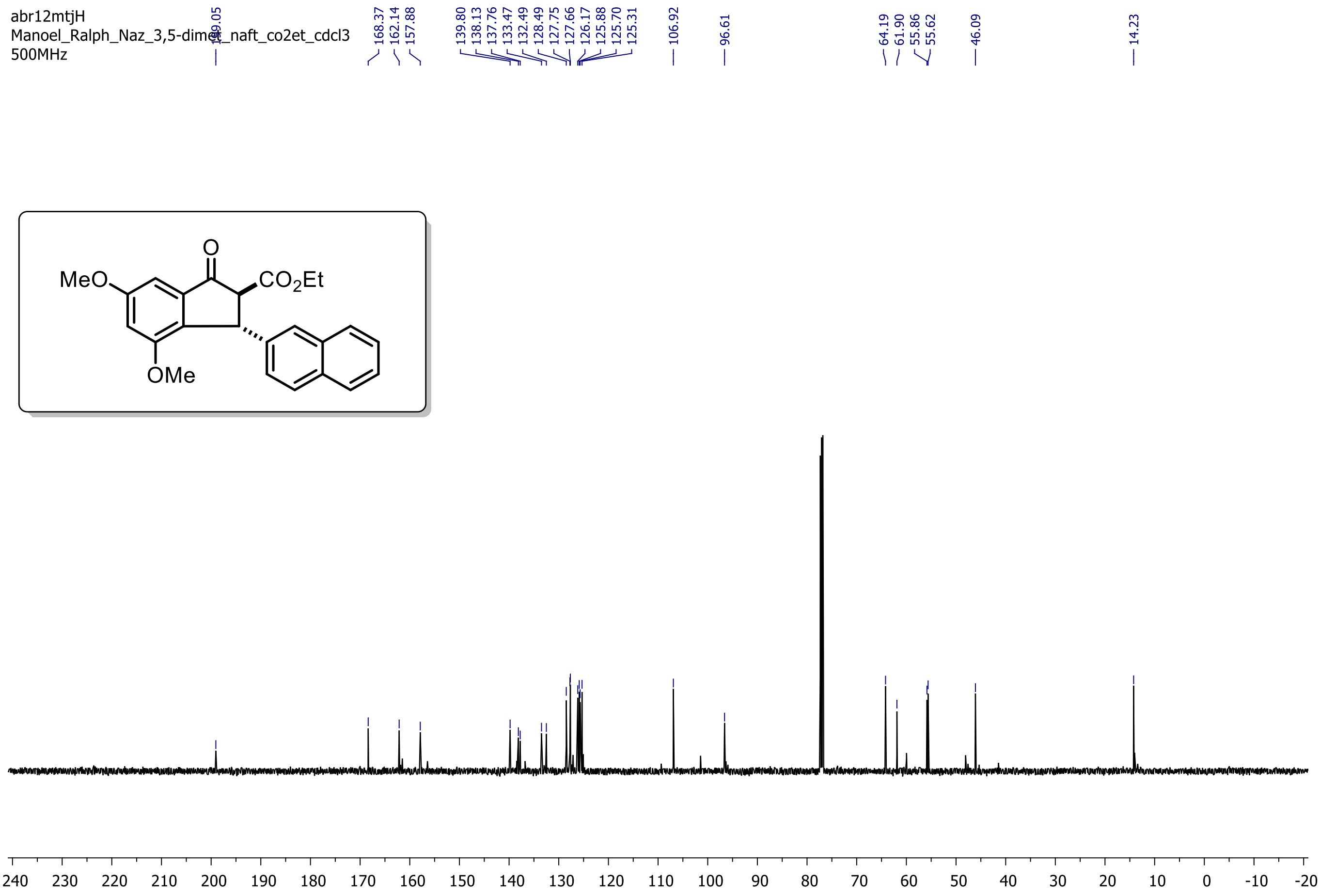

Figure S54. ^{13}C NMR spectrum (126 MHz, $CDCl_3$) of compound **10bk**.

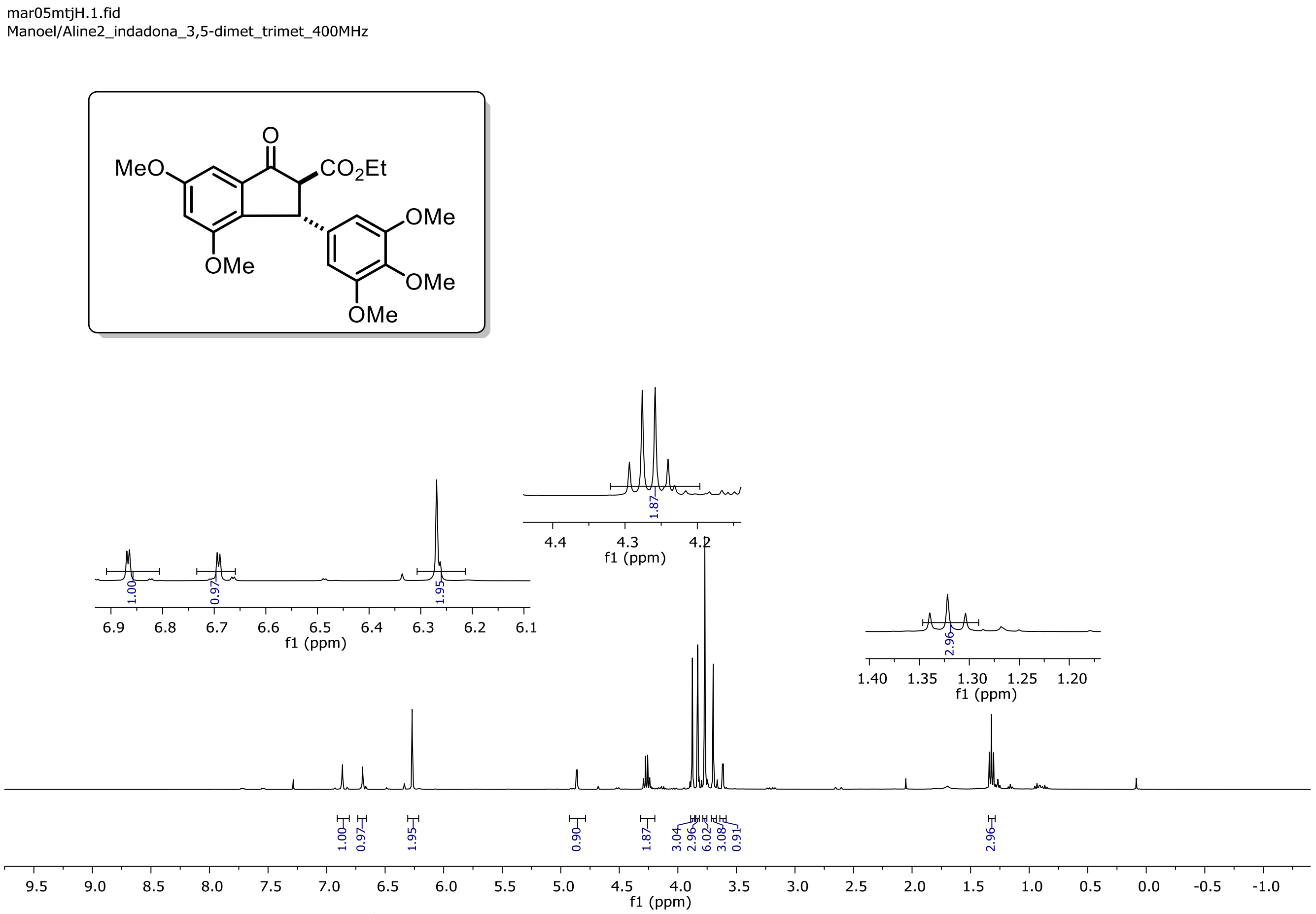

Figure S55. ^{1}H NMR spectrum (400 MHz, $CDCl_3$) of compound **10bc**.

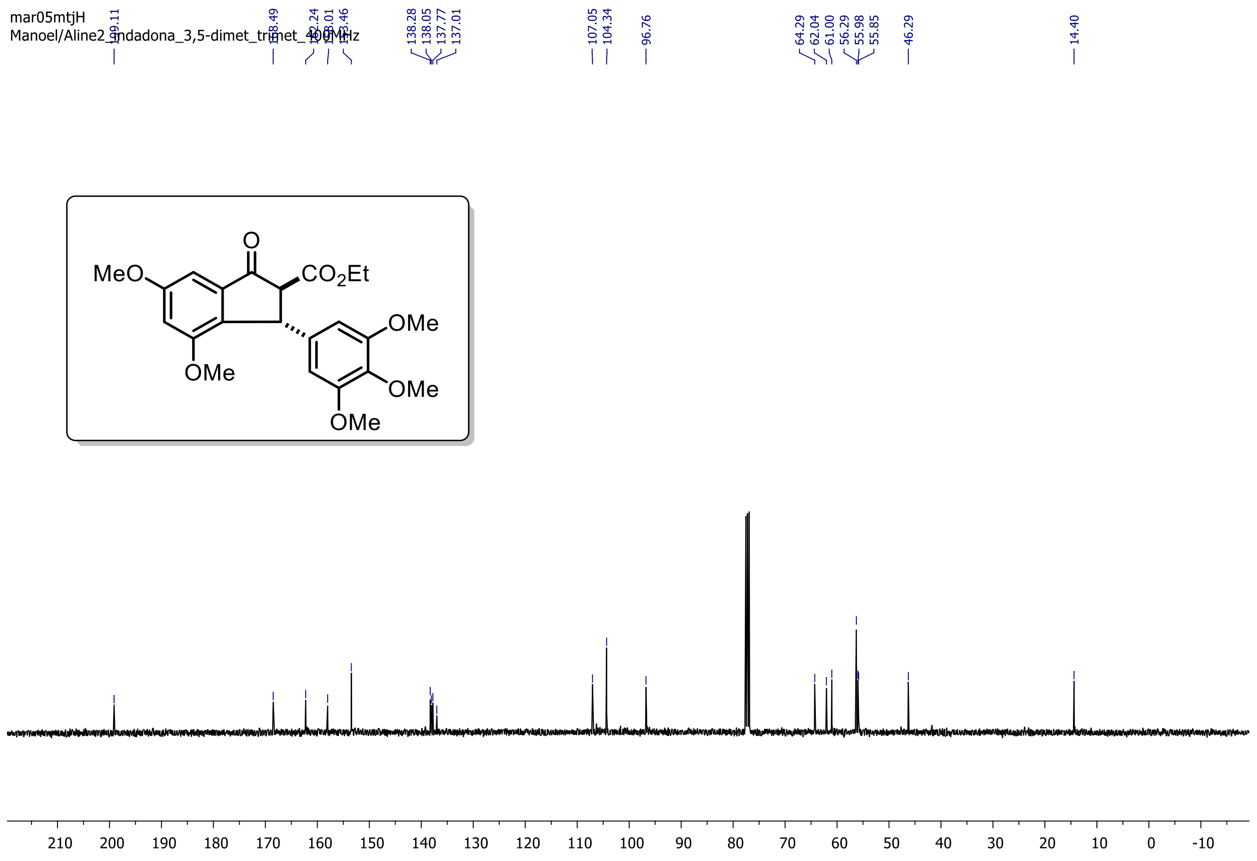

Figure S56. ^{13}C NMR spectrum (101 MHz, $CDCl_3$) of compound **10bc**.

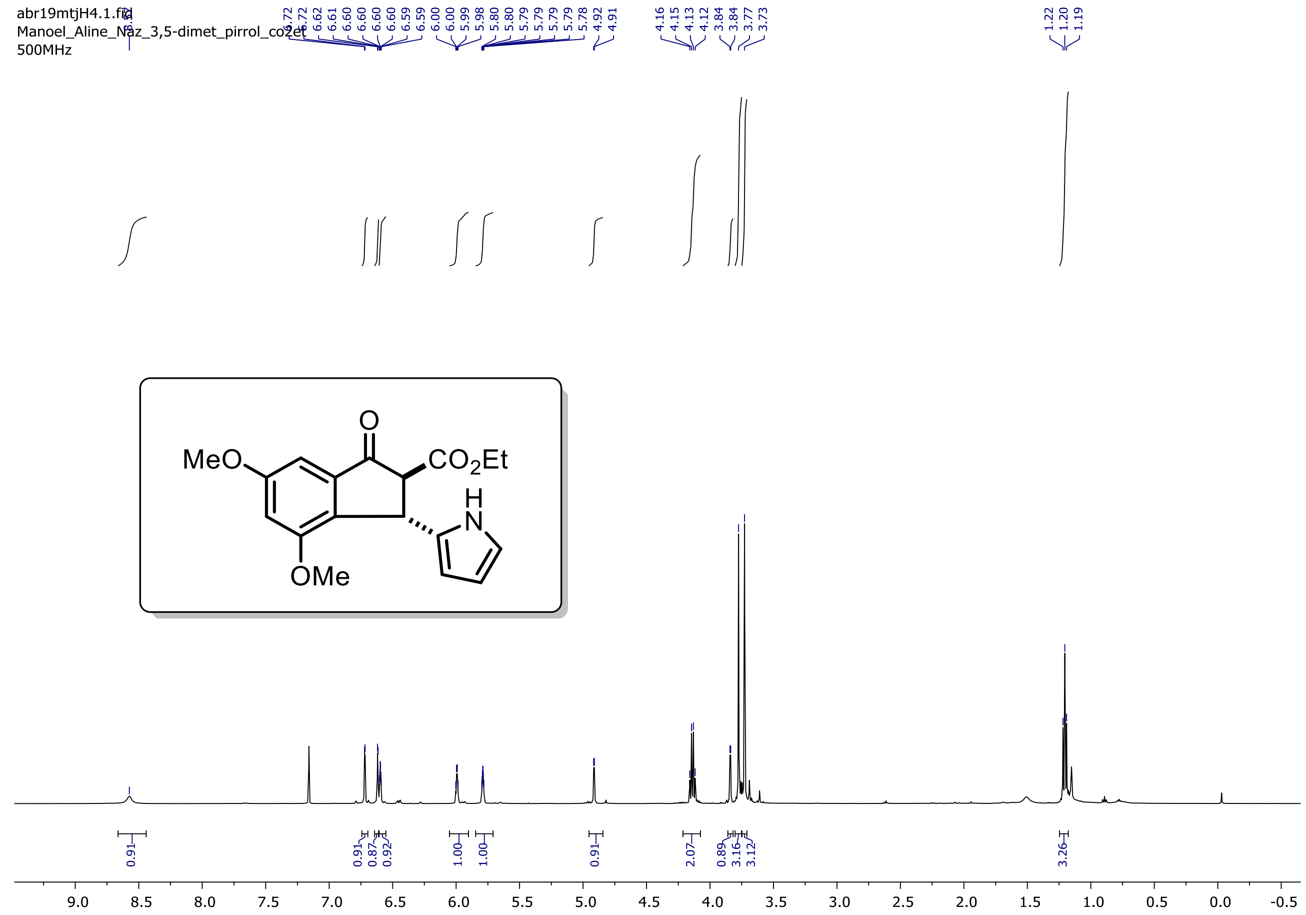

Figure S57. ^{1}H NMR spectrum (500 MHz, $CDCl_3$) of compound **10bg**.

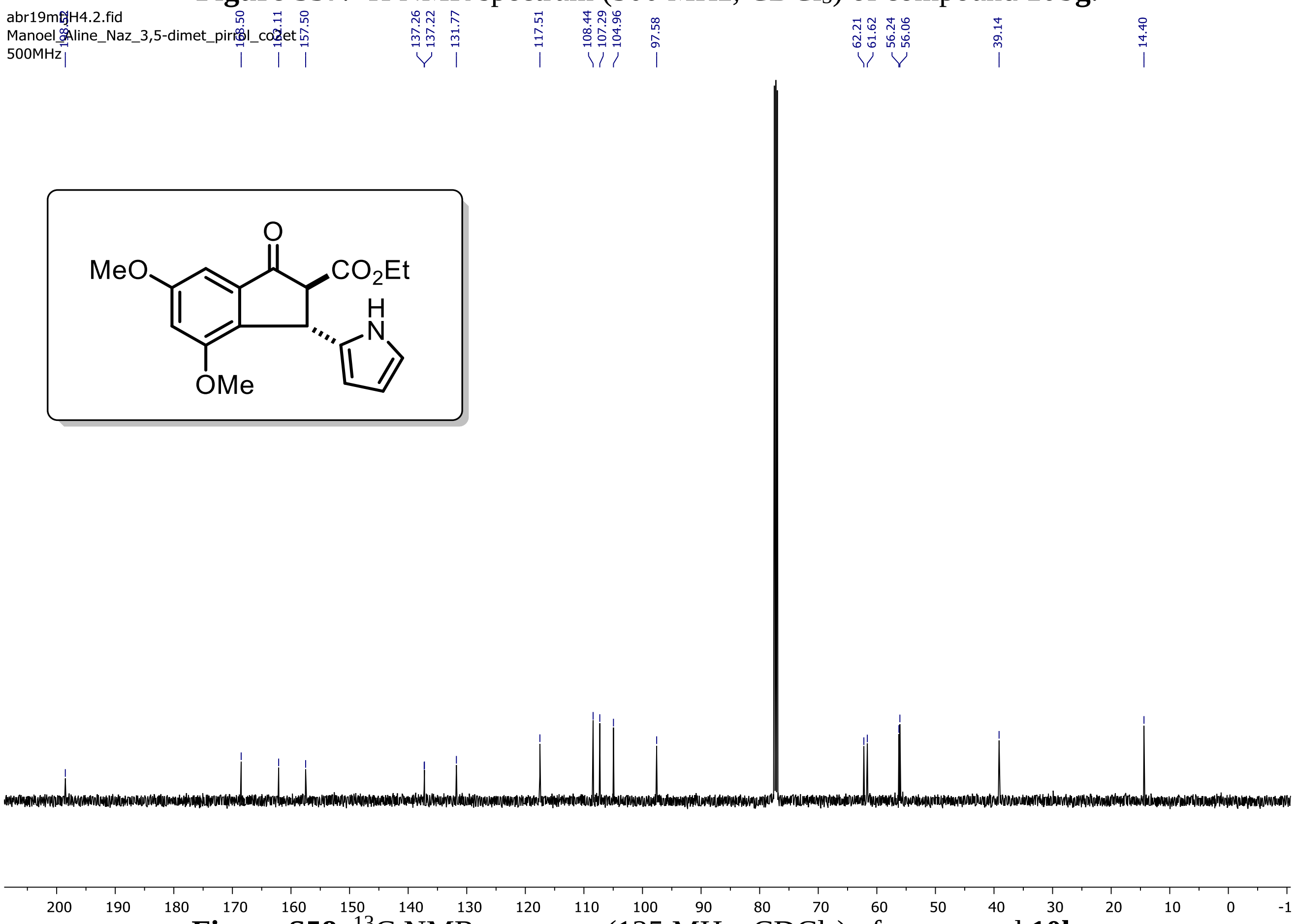

Figure S58. ^{13}C NMR spectrum (125 MHz, $CDCl_3$) of compound **10bg**.

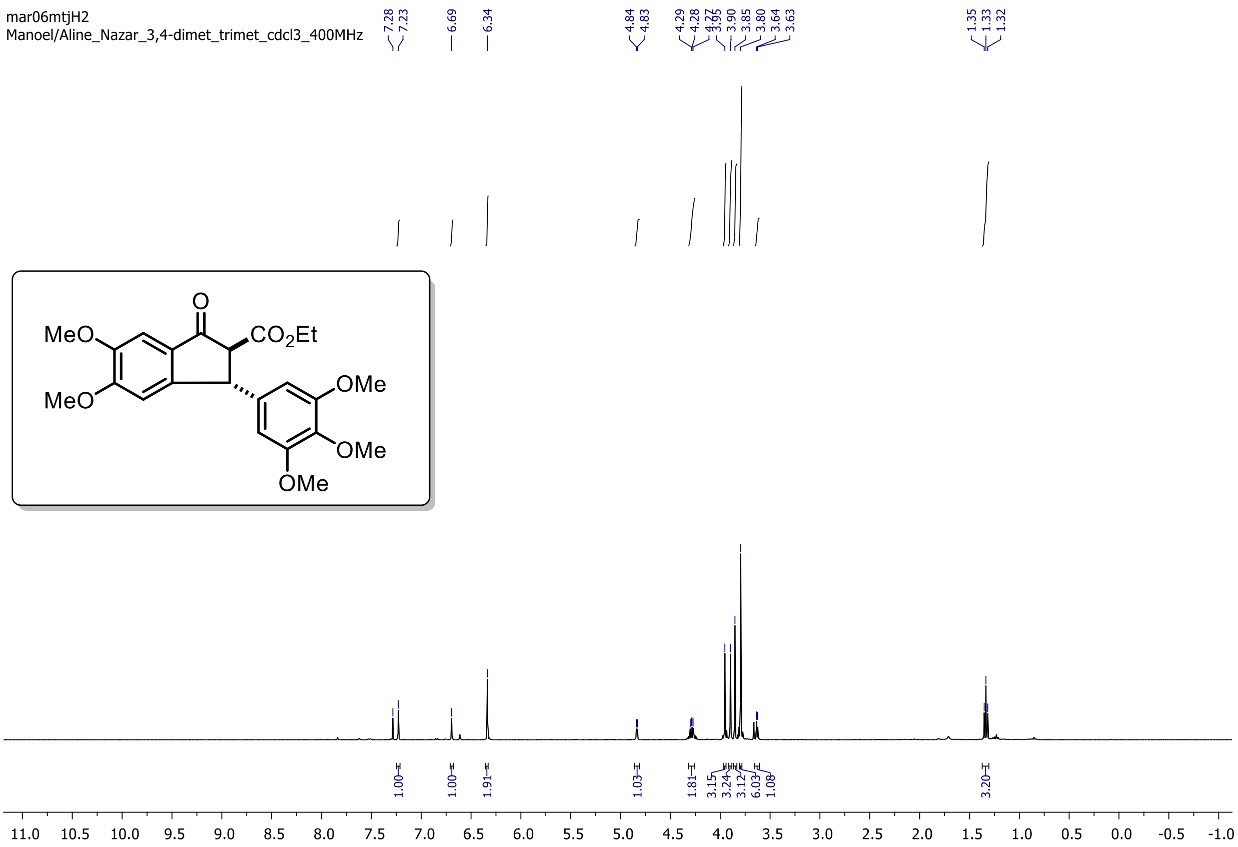

Figure S59. ^{1}H NMR spectrum (400 MHz, $CDCl_3$) of compound **10cc**.

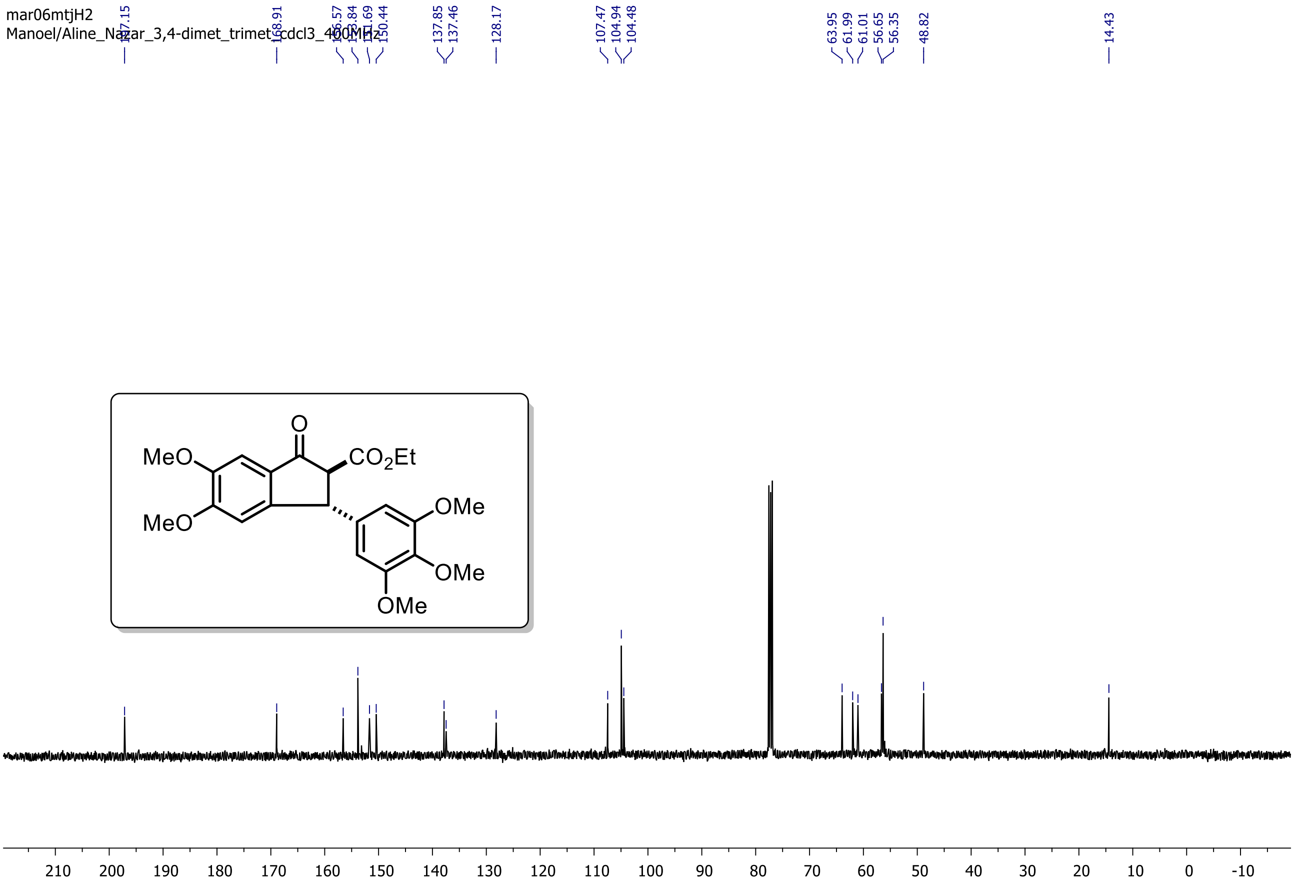

Figure S60. ^{13}C NMR spectrum (101 MHz, $CDCl_3$) of compound **10cc**.

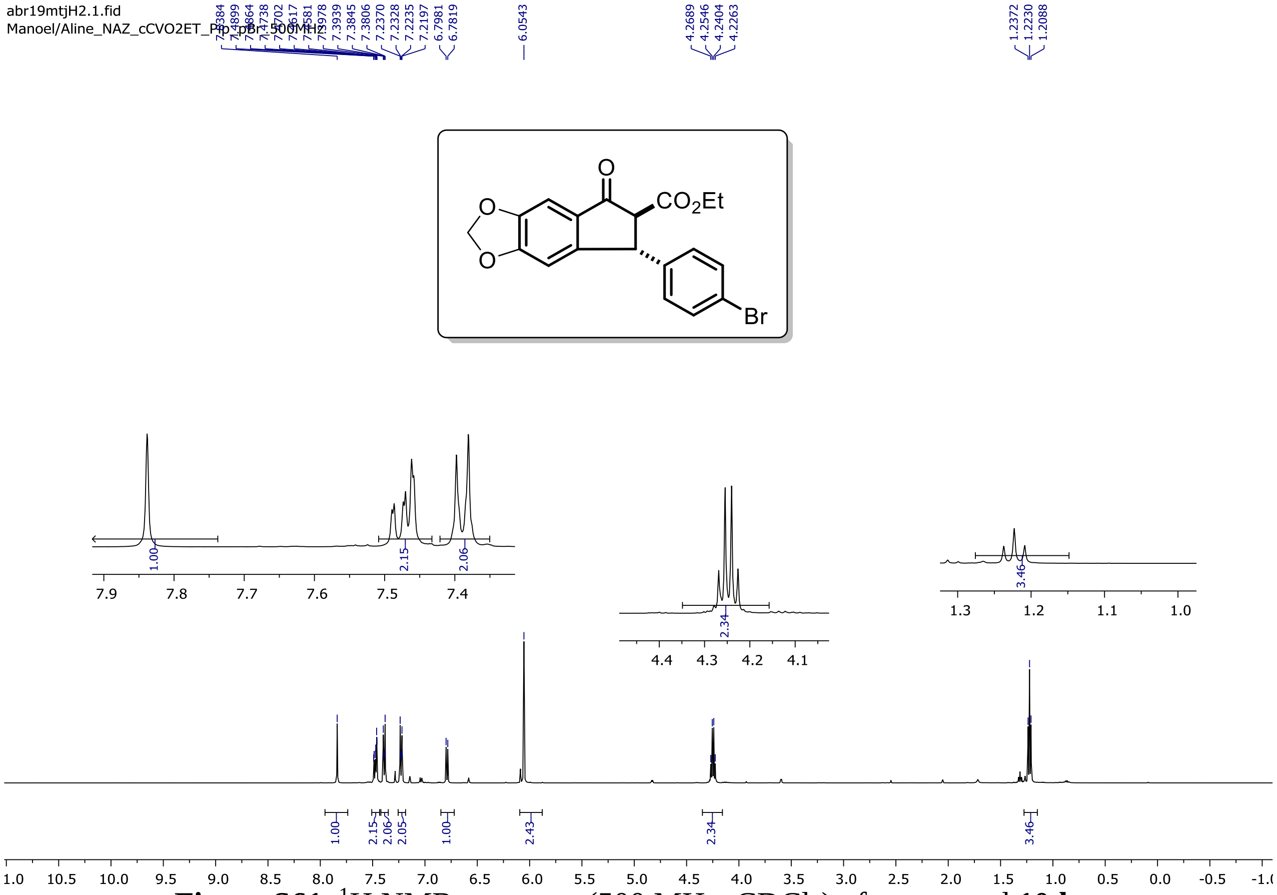

Figure S61. ^{1}H NMR spectrum (500 MHz, $CDCl_3$) of compound **10dm**.

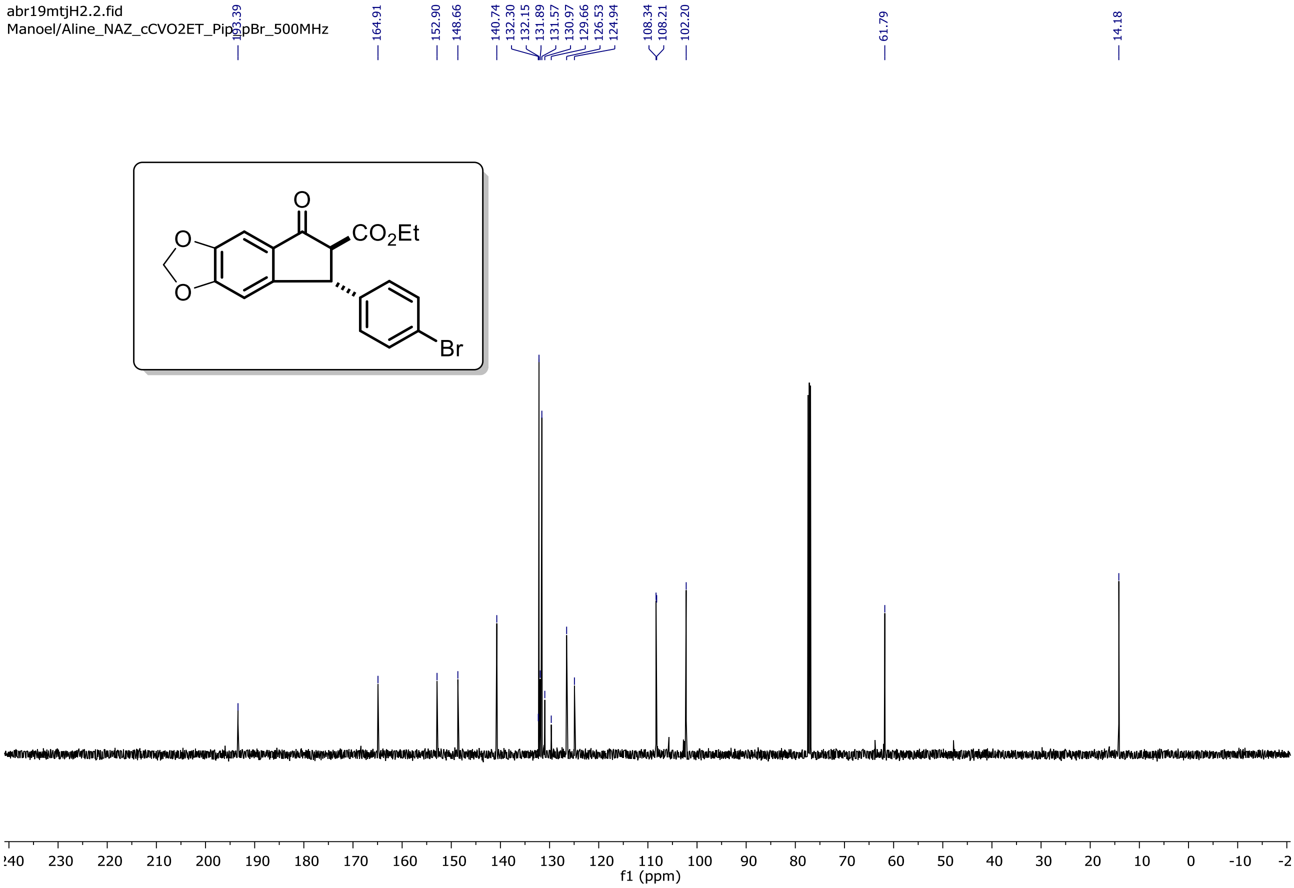

Figure S62. ^{13}C NMR spectrum (125 MHz, $CDCl_3$) of compound **10dm**.

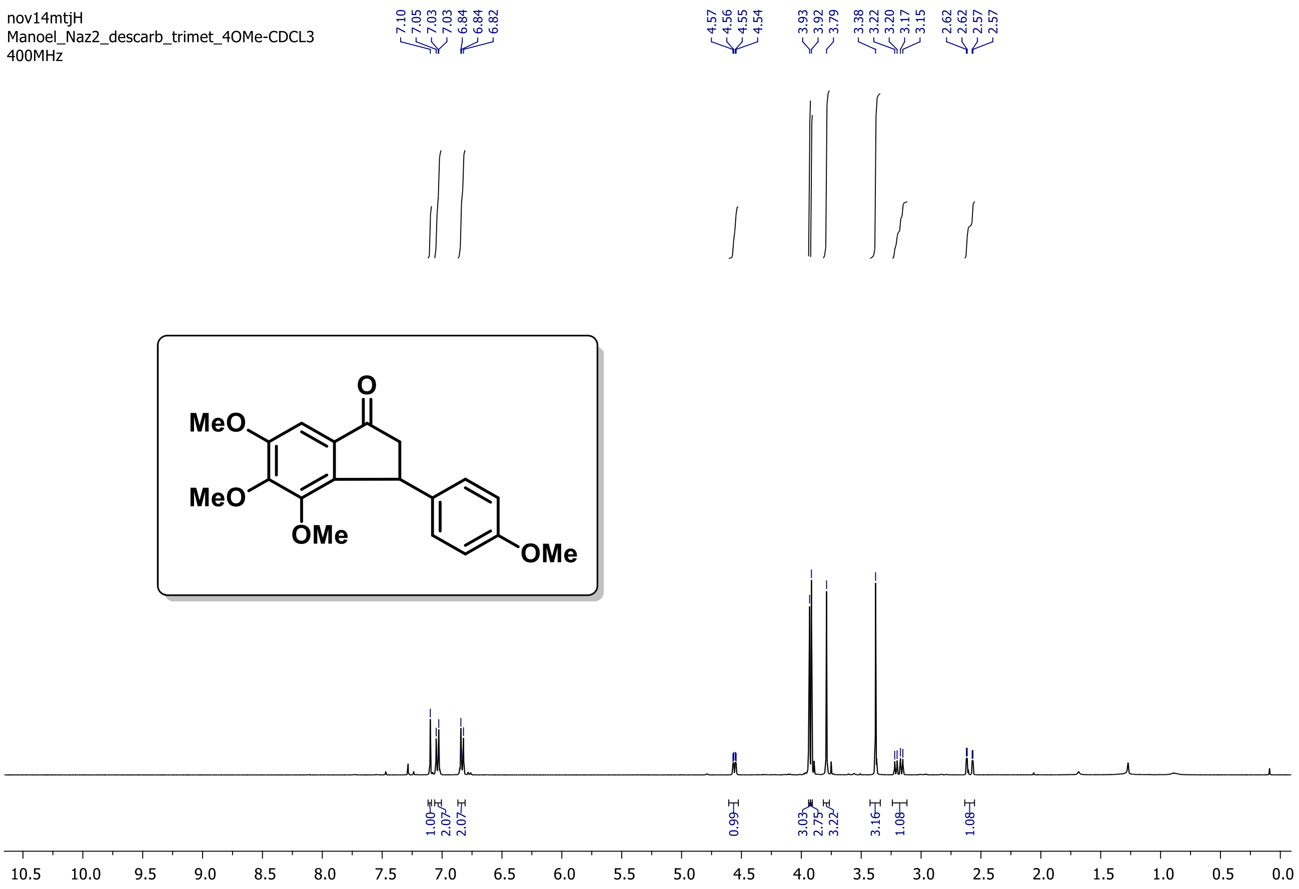

Figure S63. ^{1}H NMR spectrum (400 MHz, $CDCl_3$) of compound **11aa**.

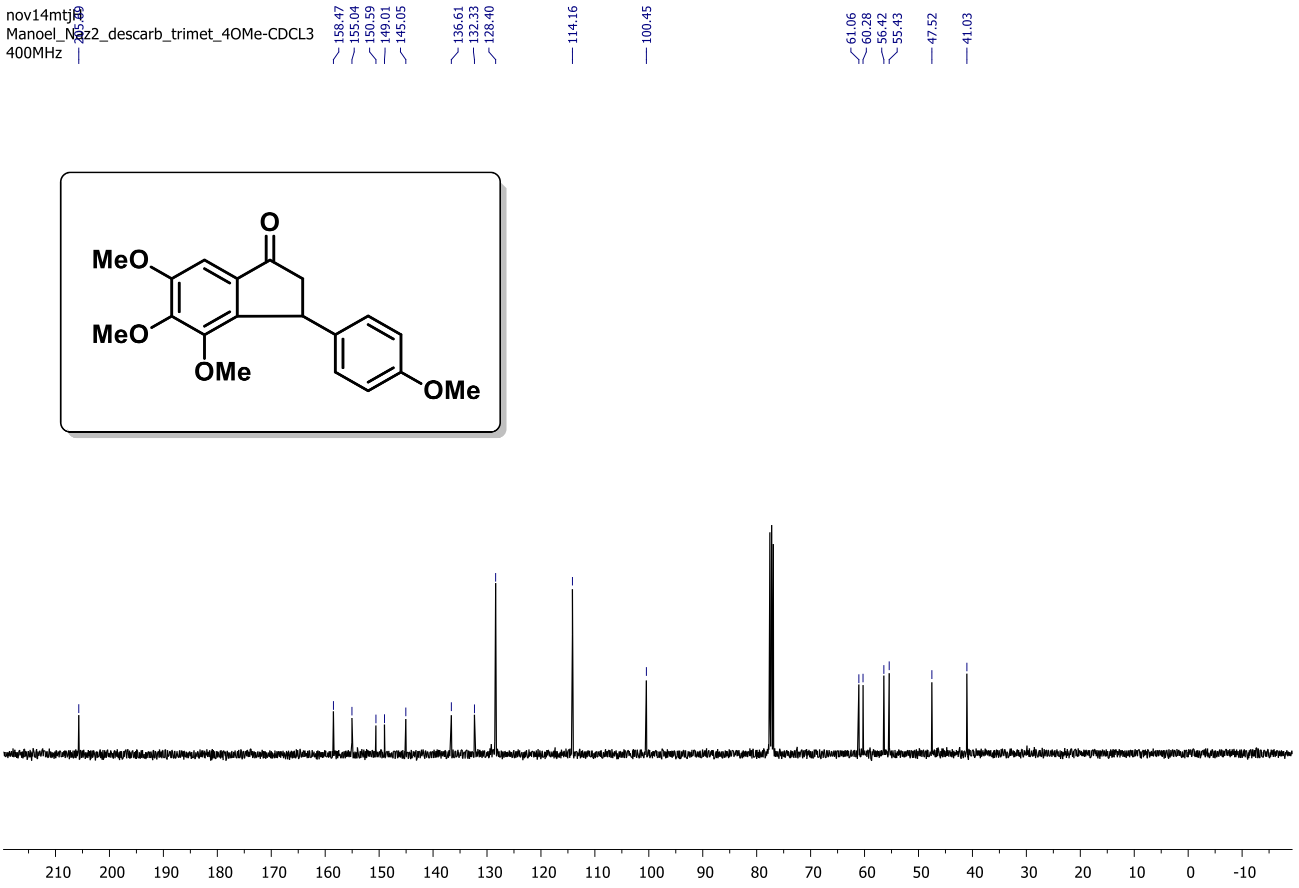

Figure S64. ^{13}C NMR spectrum (101 MHz, $CDCl_3$) of compound **11aa**.

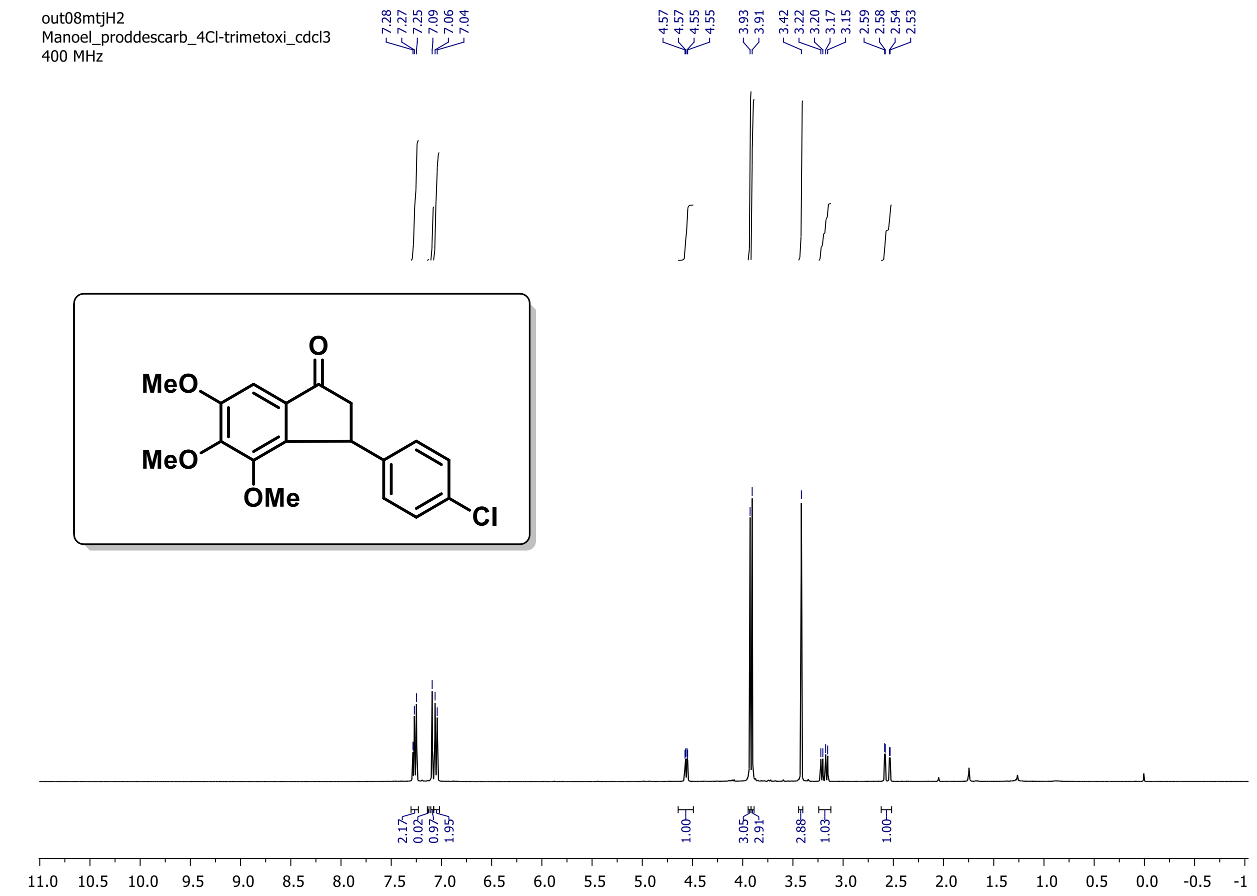

Figure S65. ^{1}H NMR spectrum (400 MHz, $CDCl_3$) of compound **11ab**.

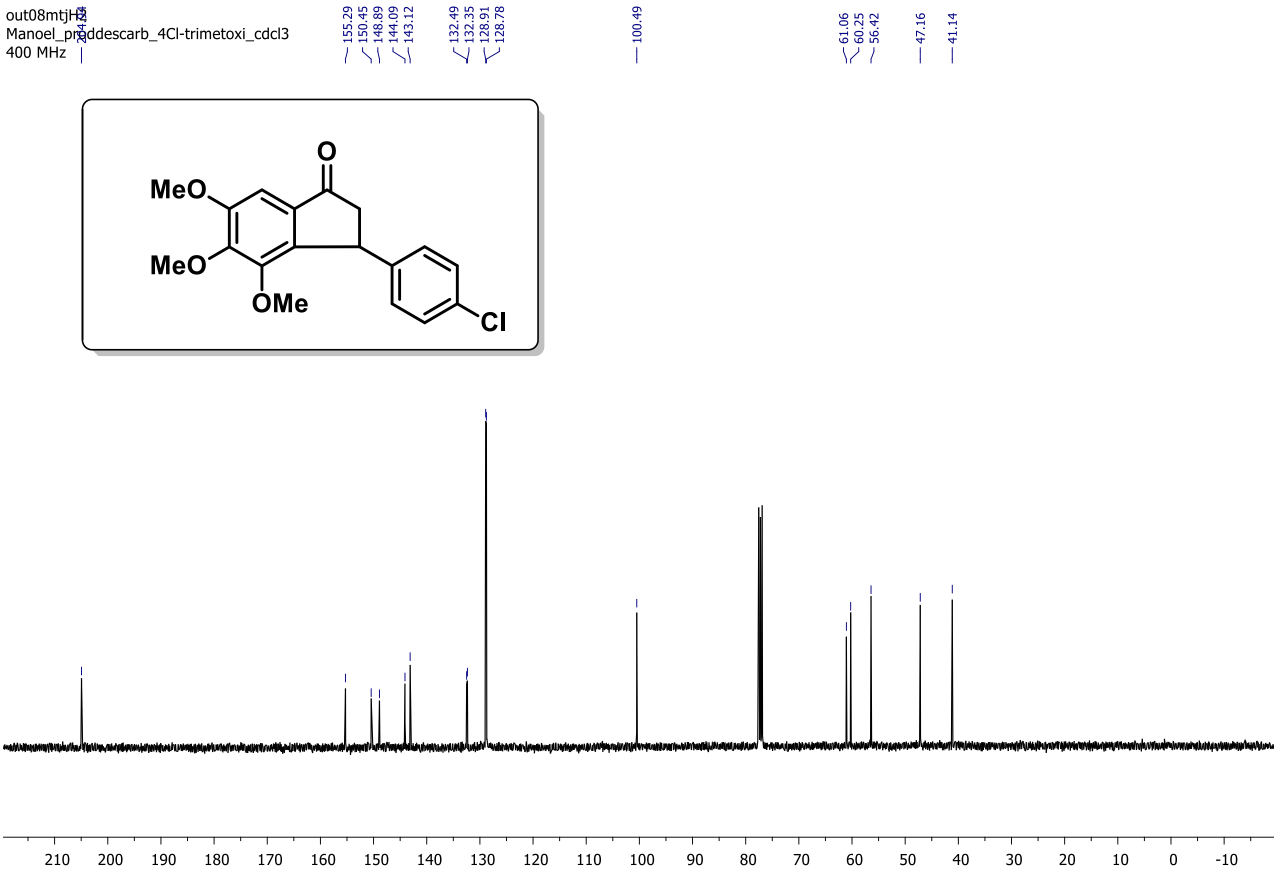

Figure S66. ^{13}C NMR spectrum (101 MHz, $CDCl_3$) of compound **11ab**.

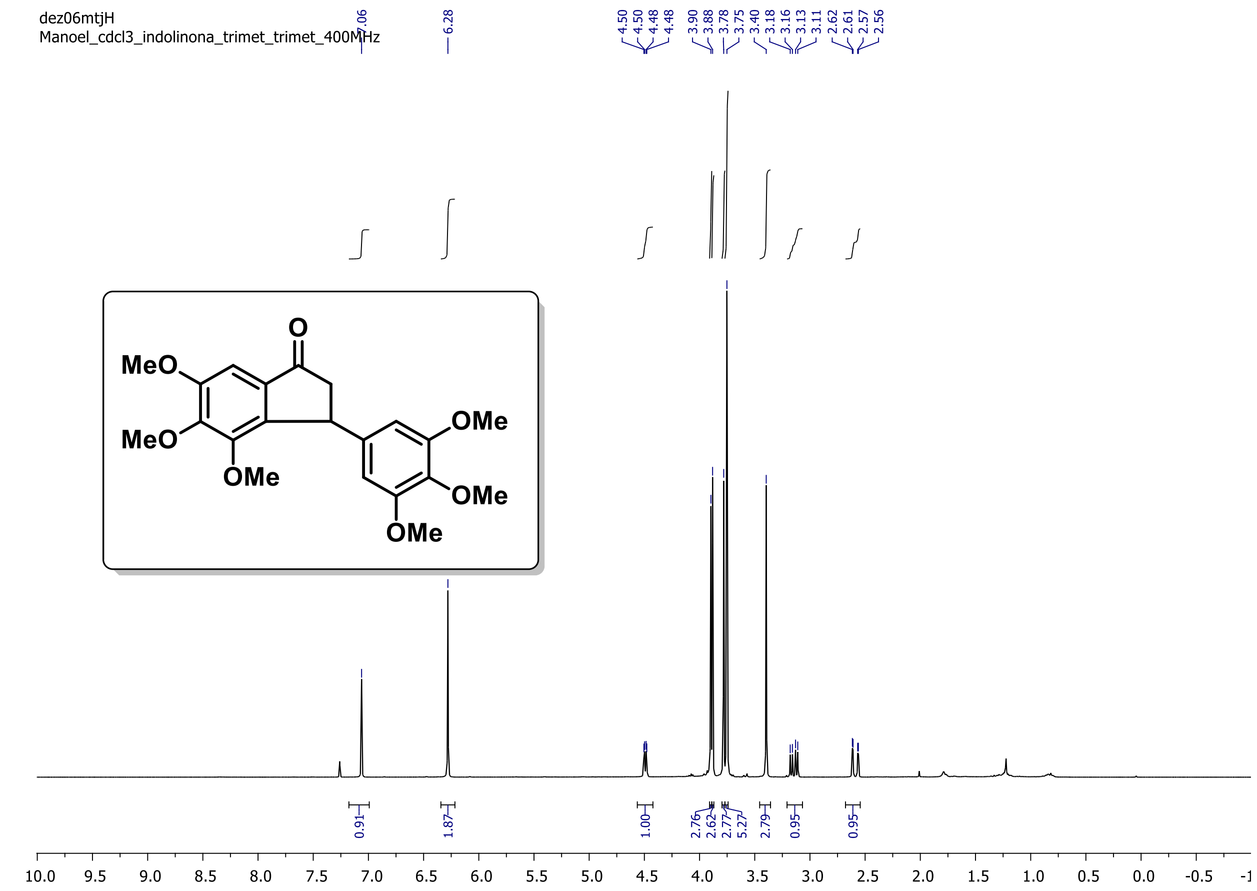

Figure S67. ^{1}H NMR spectrum (400 MHz, $CDCl_3$) of compound **11ac**.

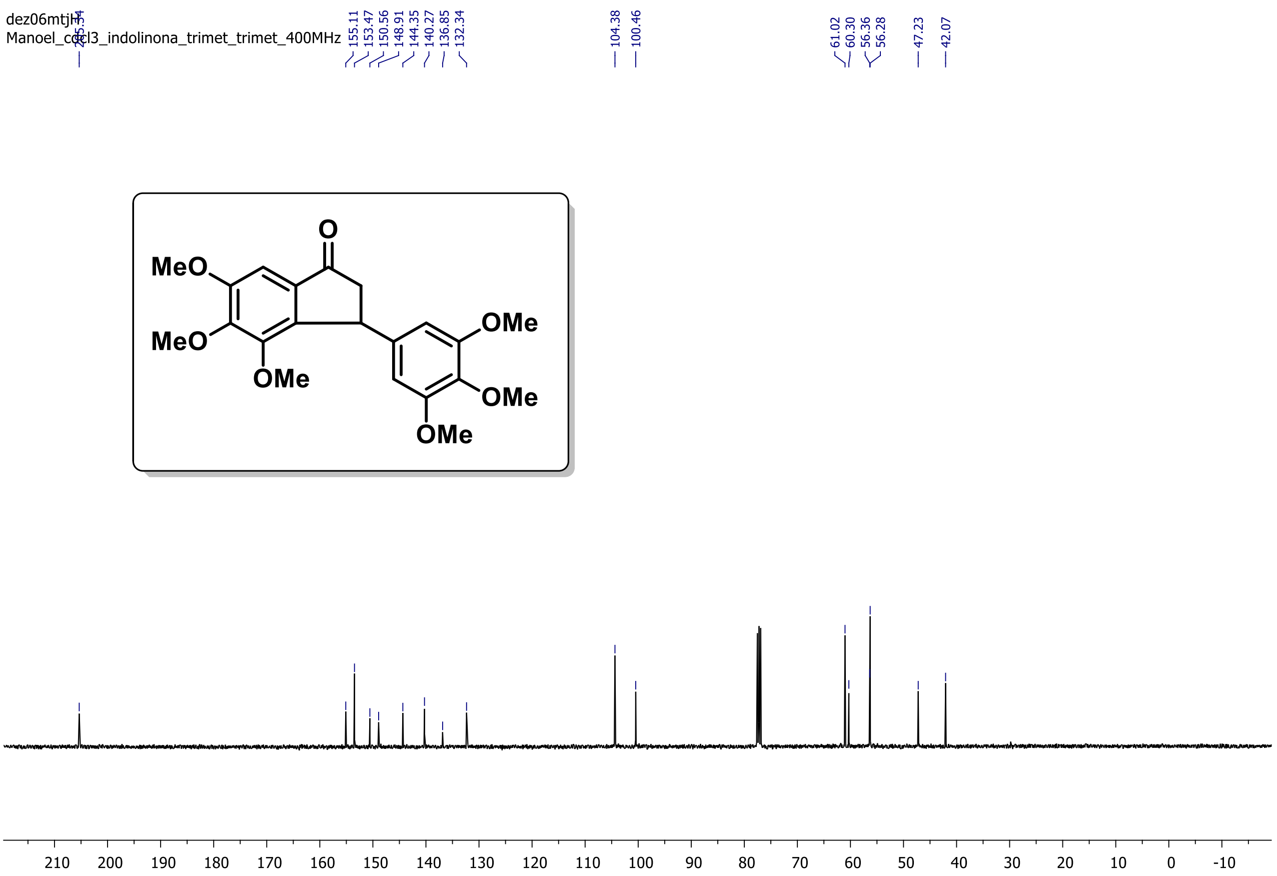

Figure S68. ^{13}C NMR spectrum (101 MHz, $CDCl_3$) of compound **11ac**.

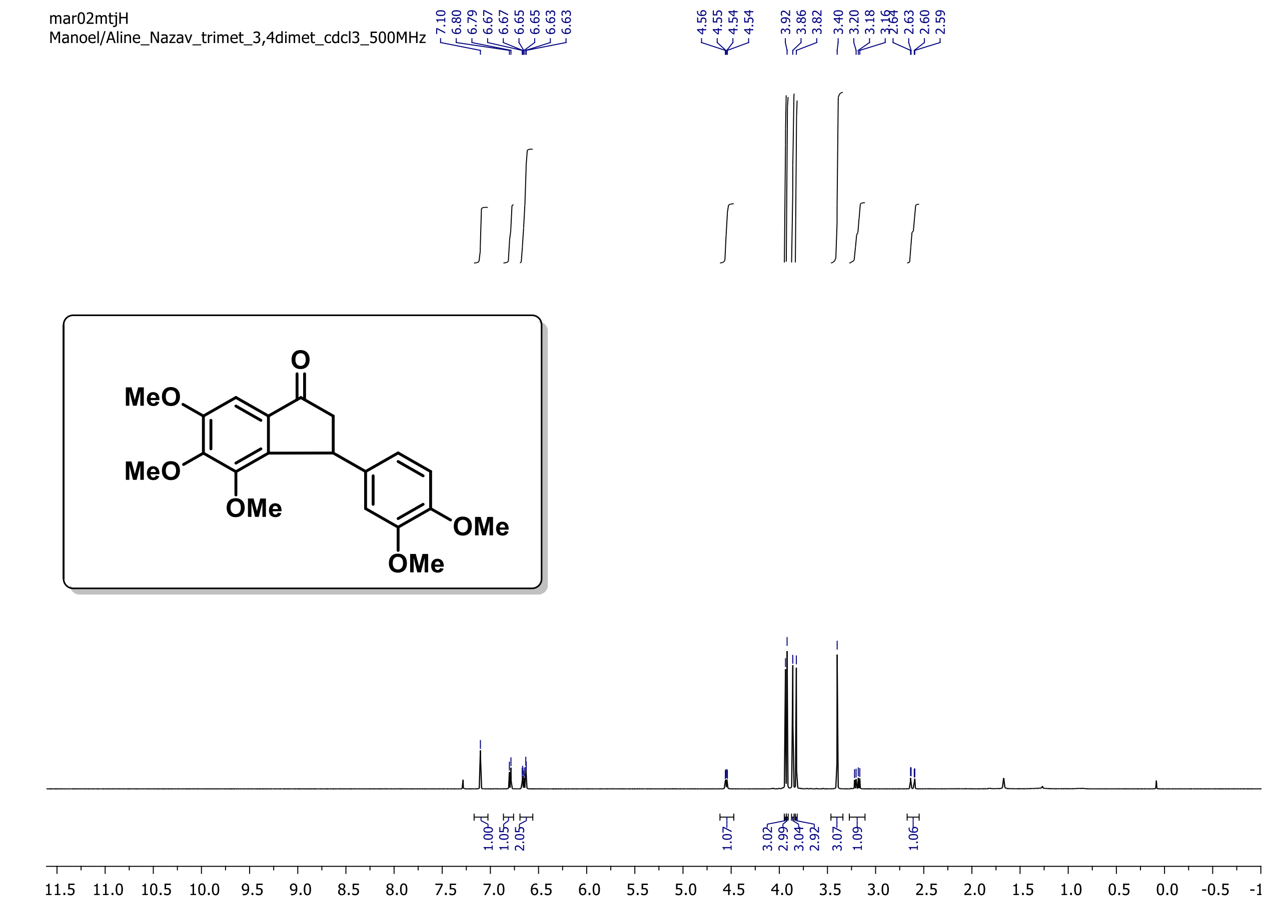

Figure S69. ^{1}H NMR spectrum (500 MHz, $CDCl_3$) of compound **11ad**.

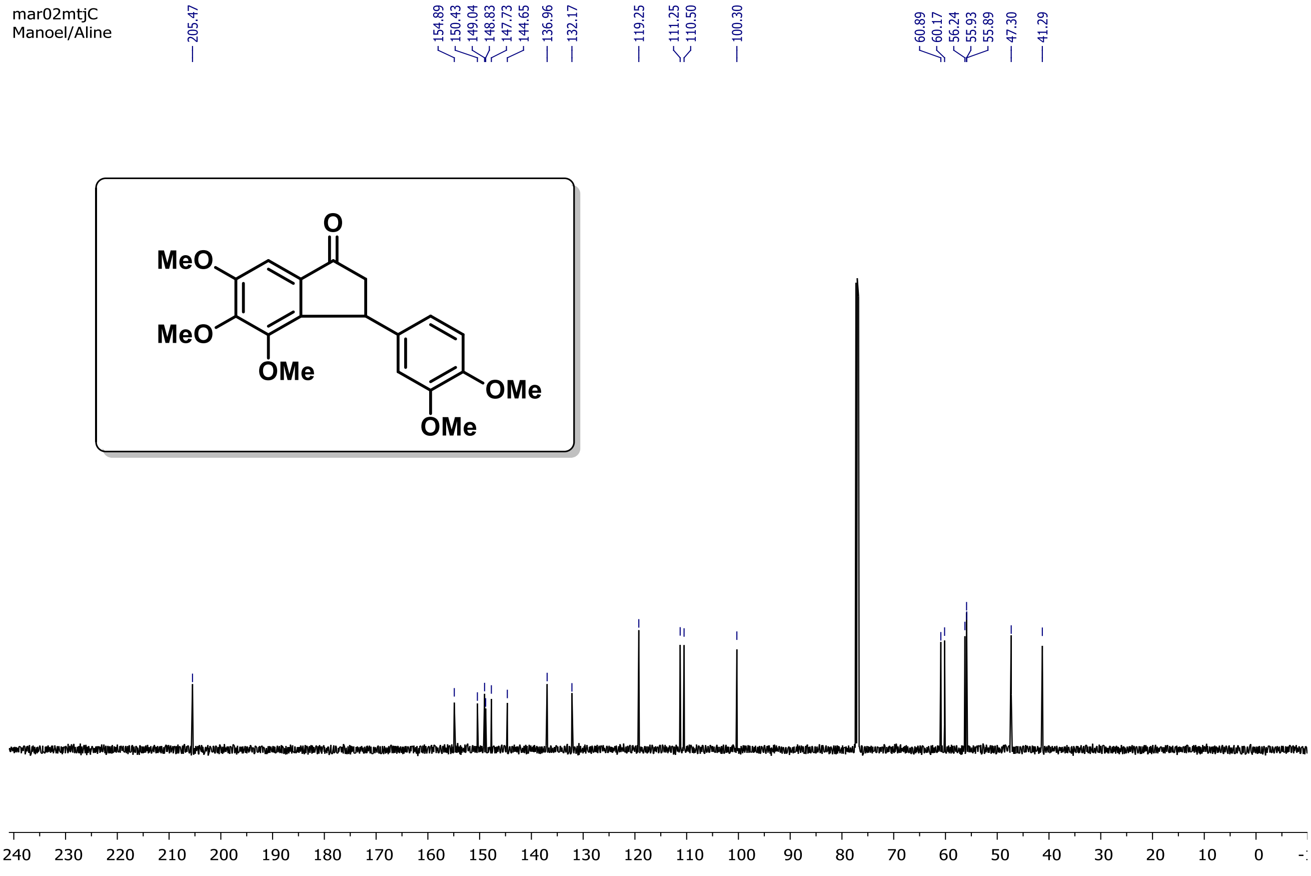

Figure S70. ^{13}C NMR spectrum (126 MHz, $CDCl_3$) of compound **11ad**.

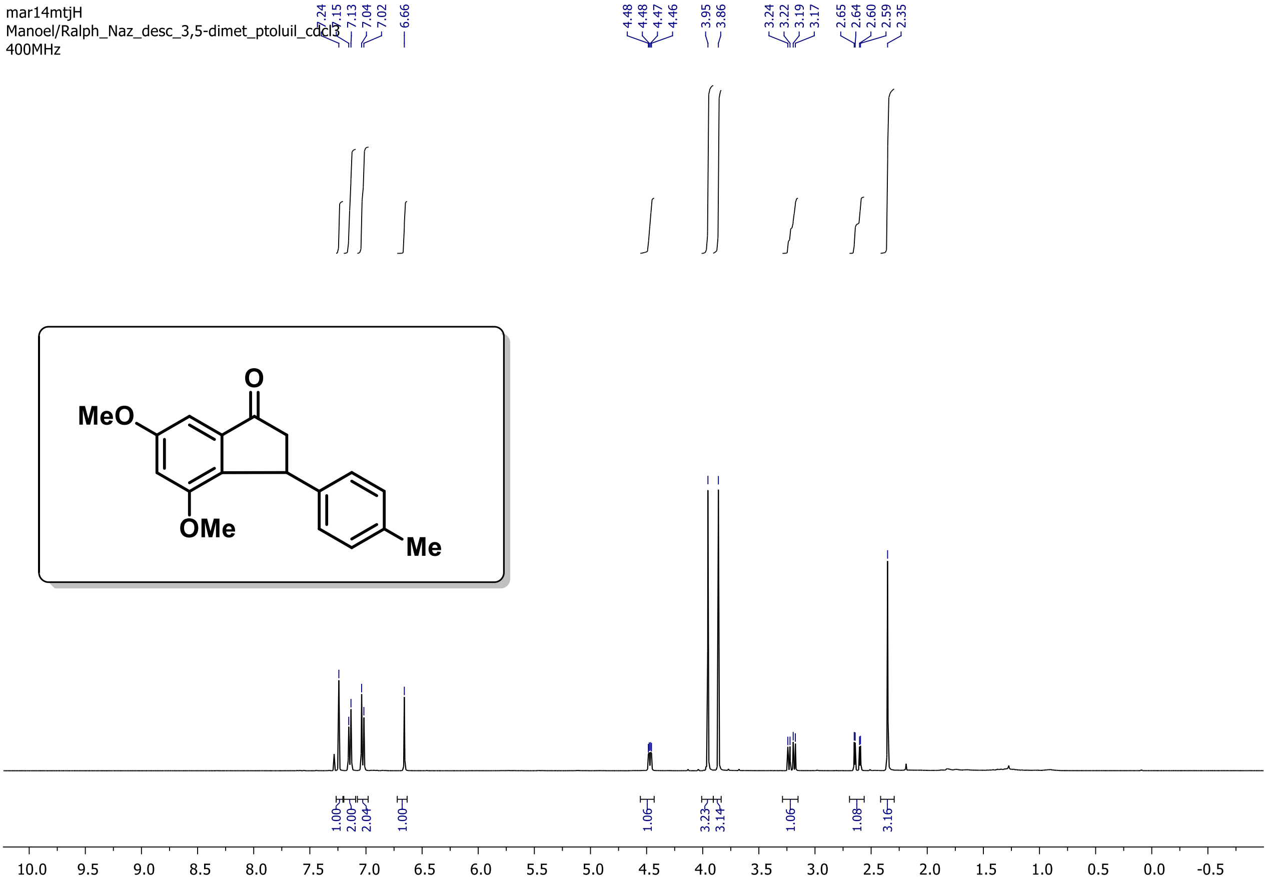

Figure S71. ^{1}H NMR spectrum (400 MHz, $CDCl_3$) of compound **11bj**.

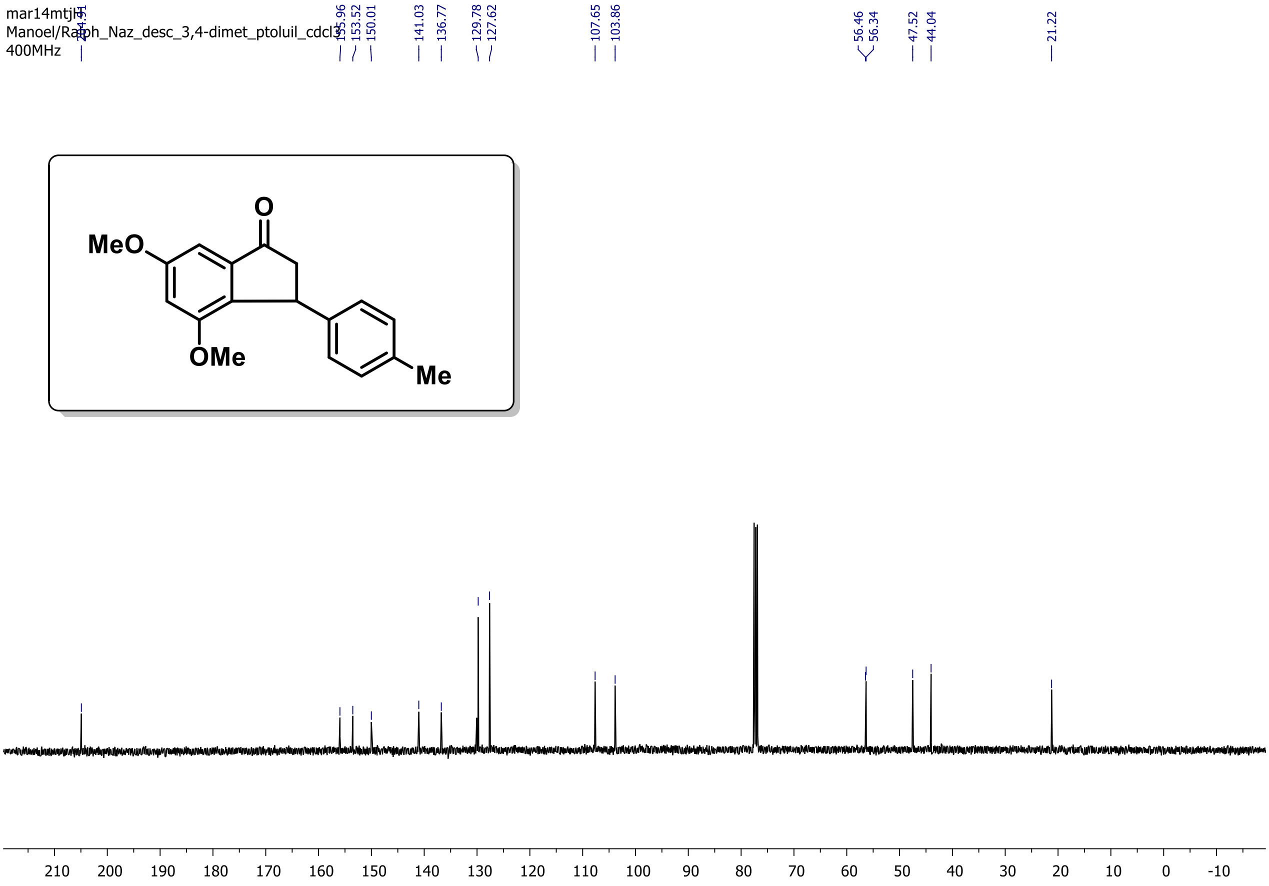

Figure S72. ^{13}C NMR spectrum (101 MHz, $CDCl_3$) of compound **11bj**.

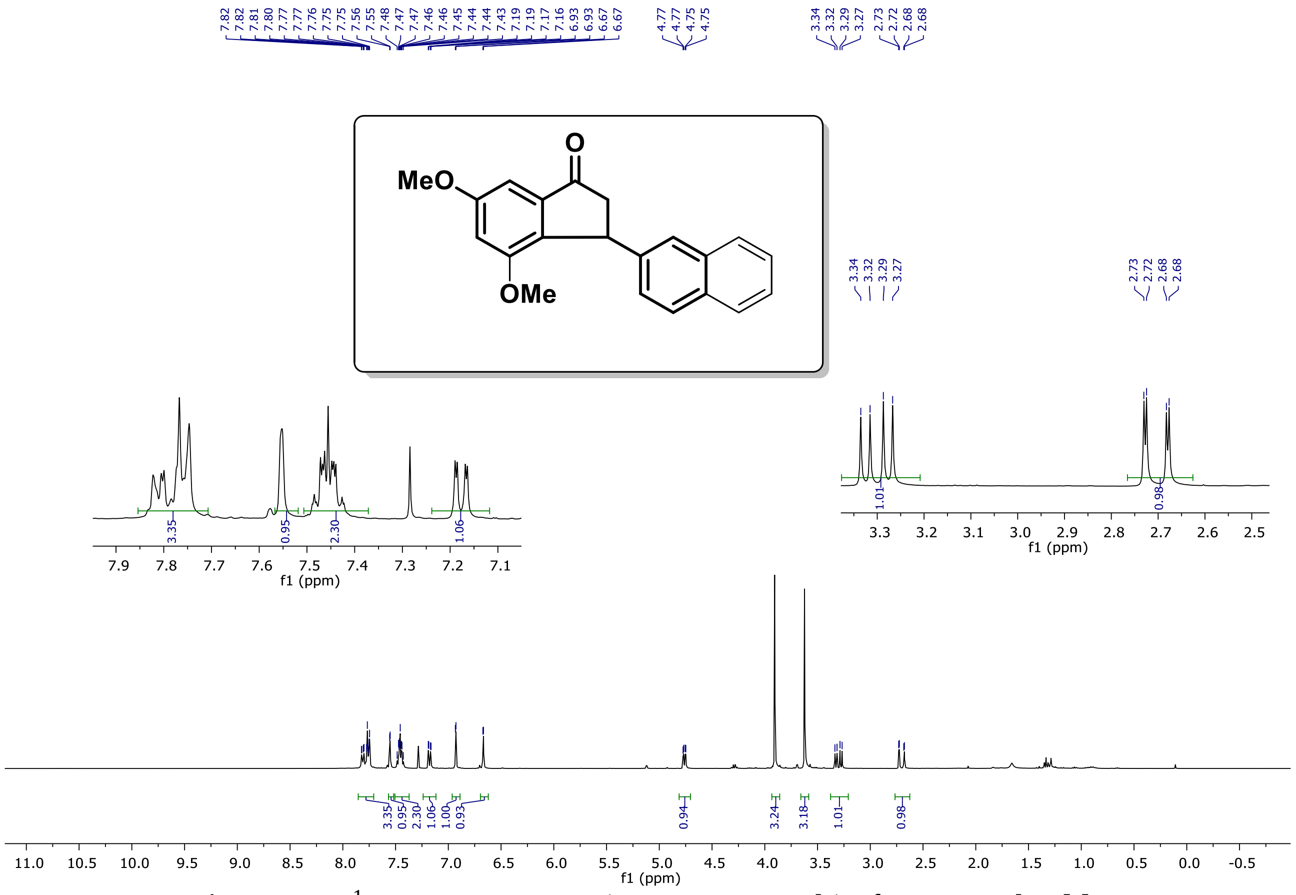

Figure S73. ^{1}H NMR spectrum (400 MHz, $CDCl_3$) of compound **11bk**.

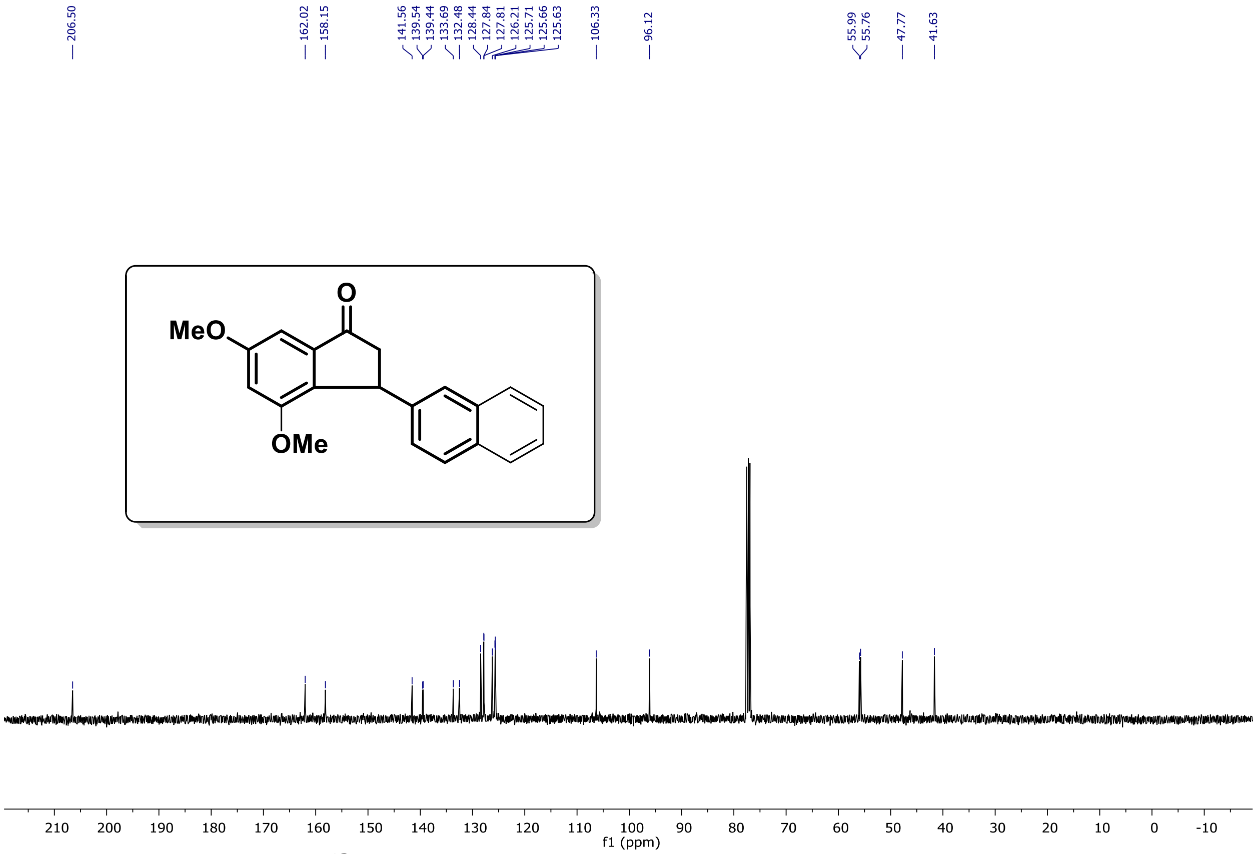

Figure S74. ^{13}C NMR spectrum (101 MHz, $CDCl_3$) of compound **11bk**.

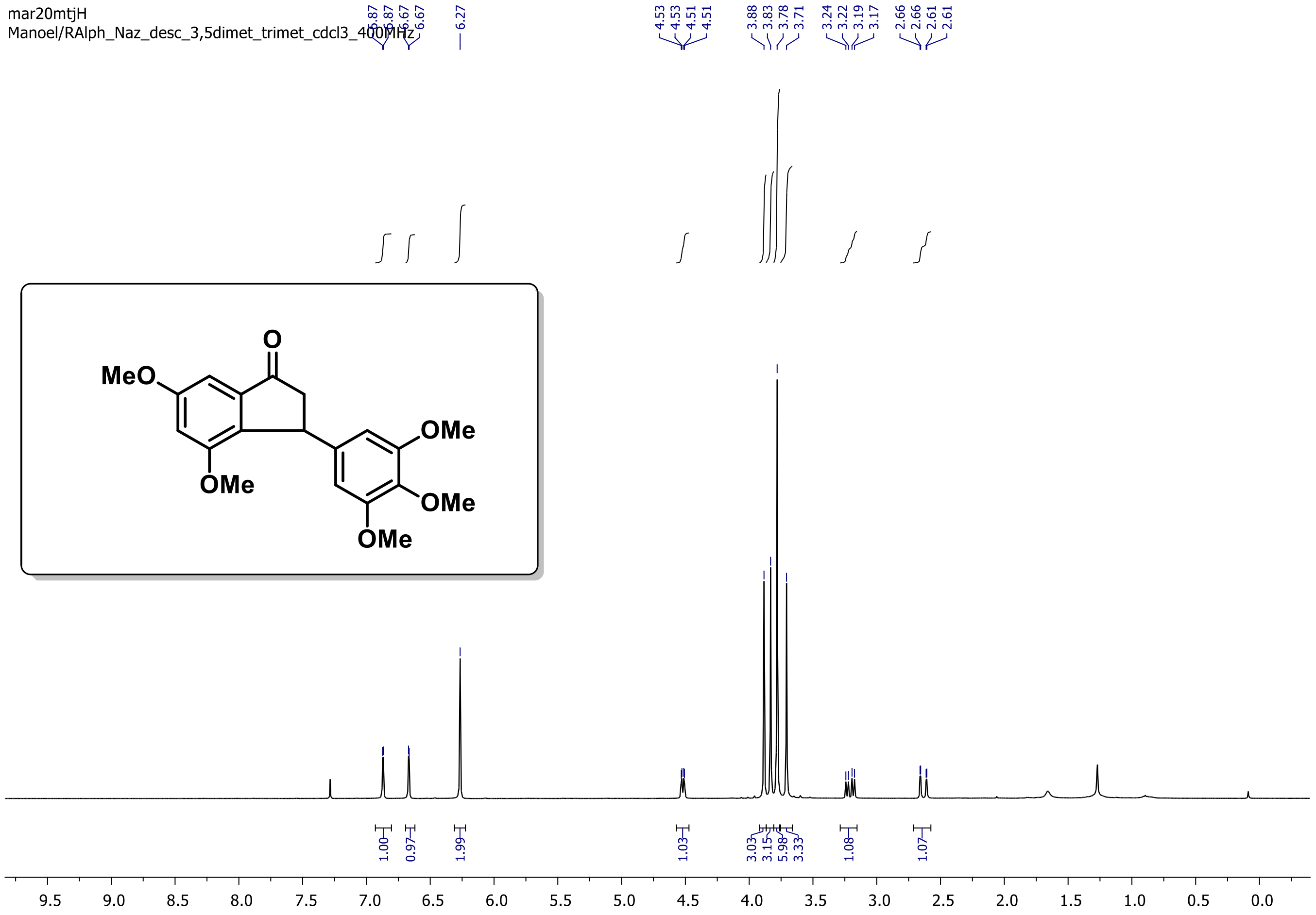

Figure S75. ^{1}H NMR spectrum (400 MHz, $CDCl_3$) of compound **11bc**.

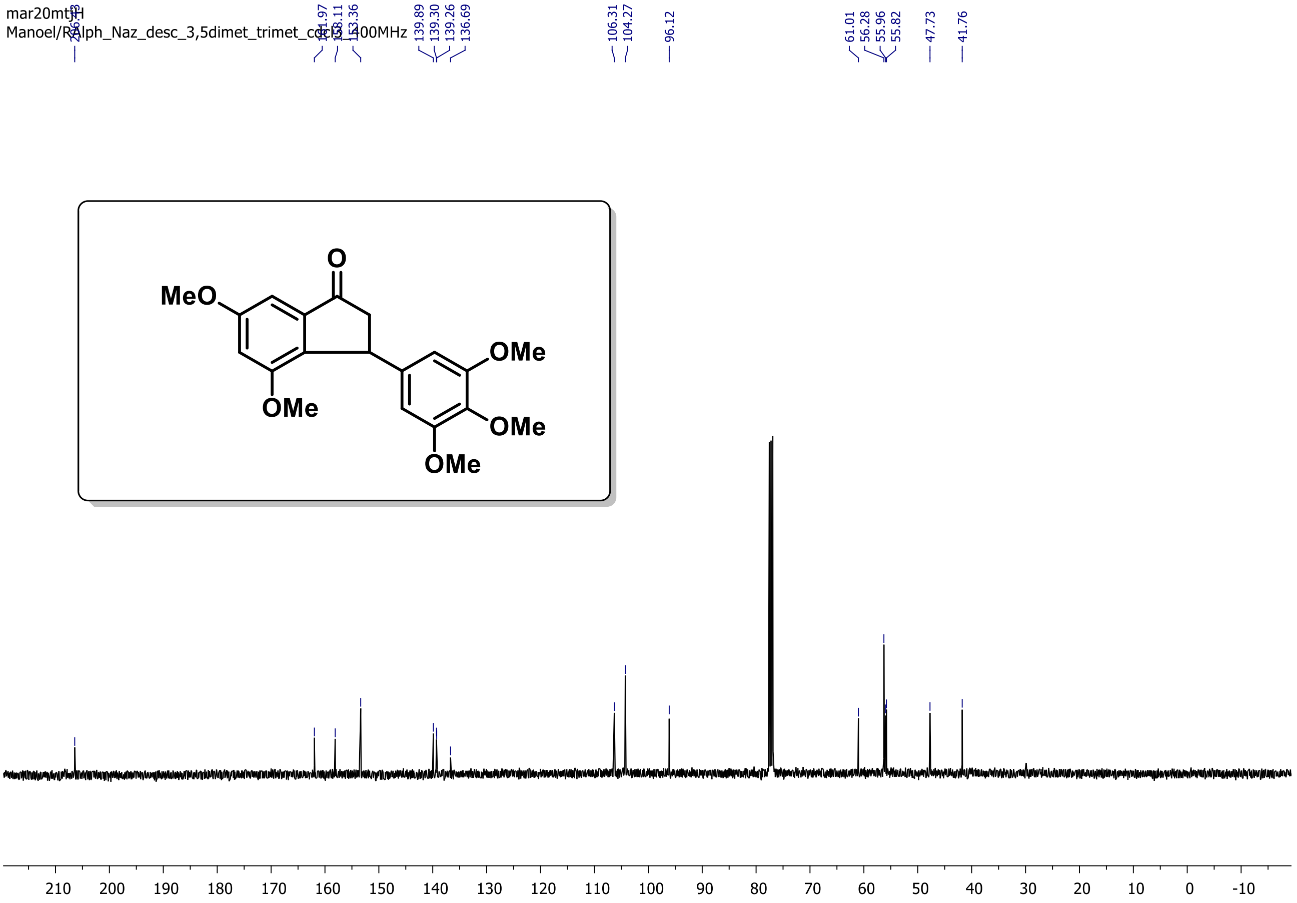

Figure S76. ^{13}C NMR spectrum (101 MHz, $CDCl_3$) of compound **11bc**.

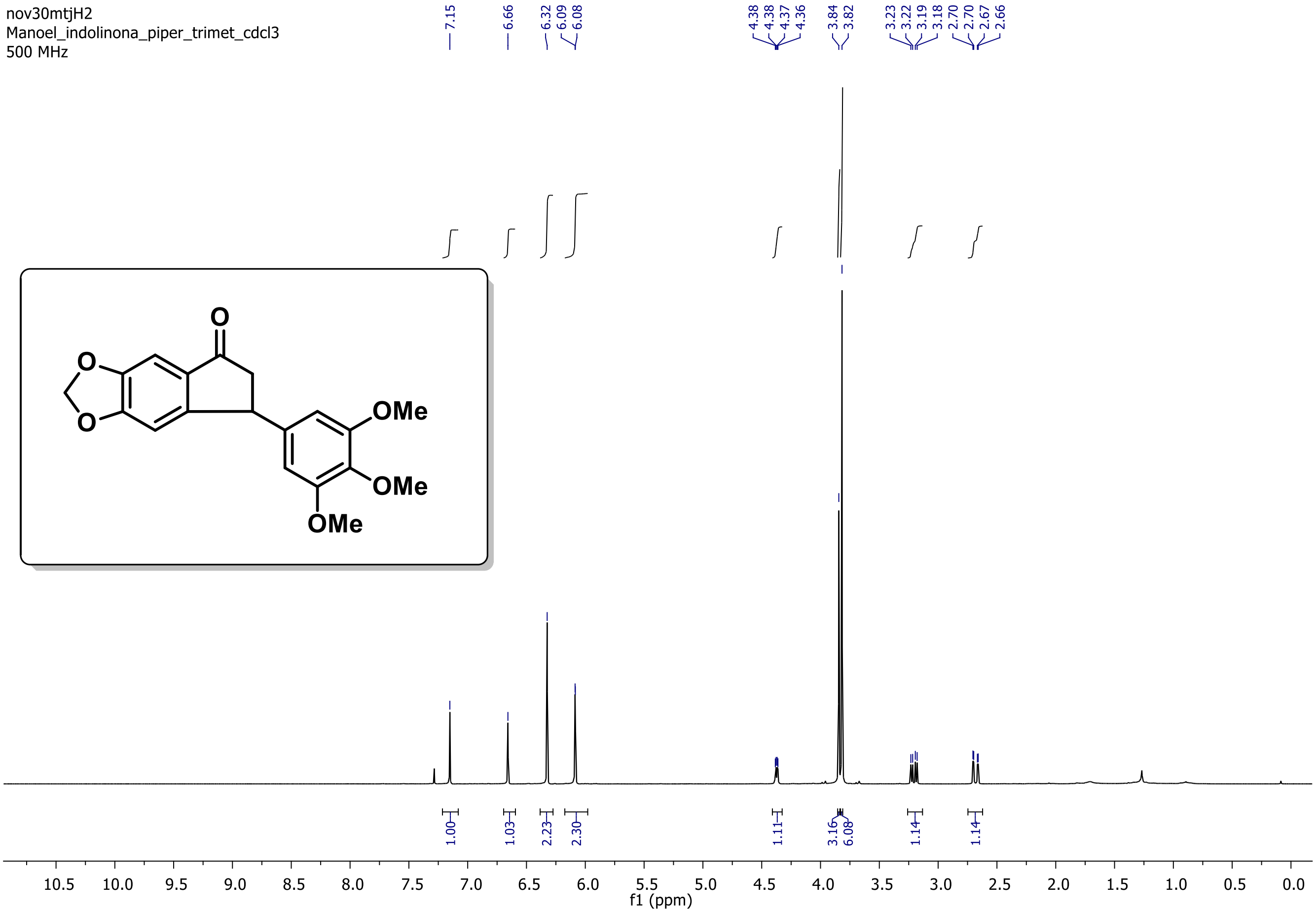

Figure S77. ^{1}H NMR spectrum (500 MHz, $CDCl_3$) of compound **11dc**.

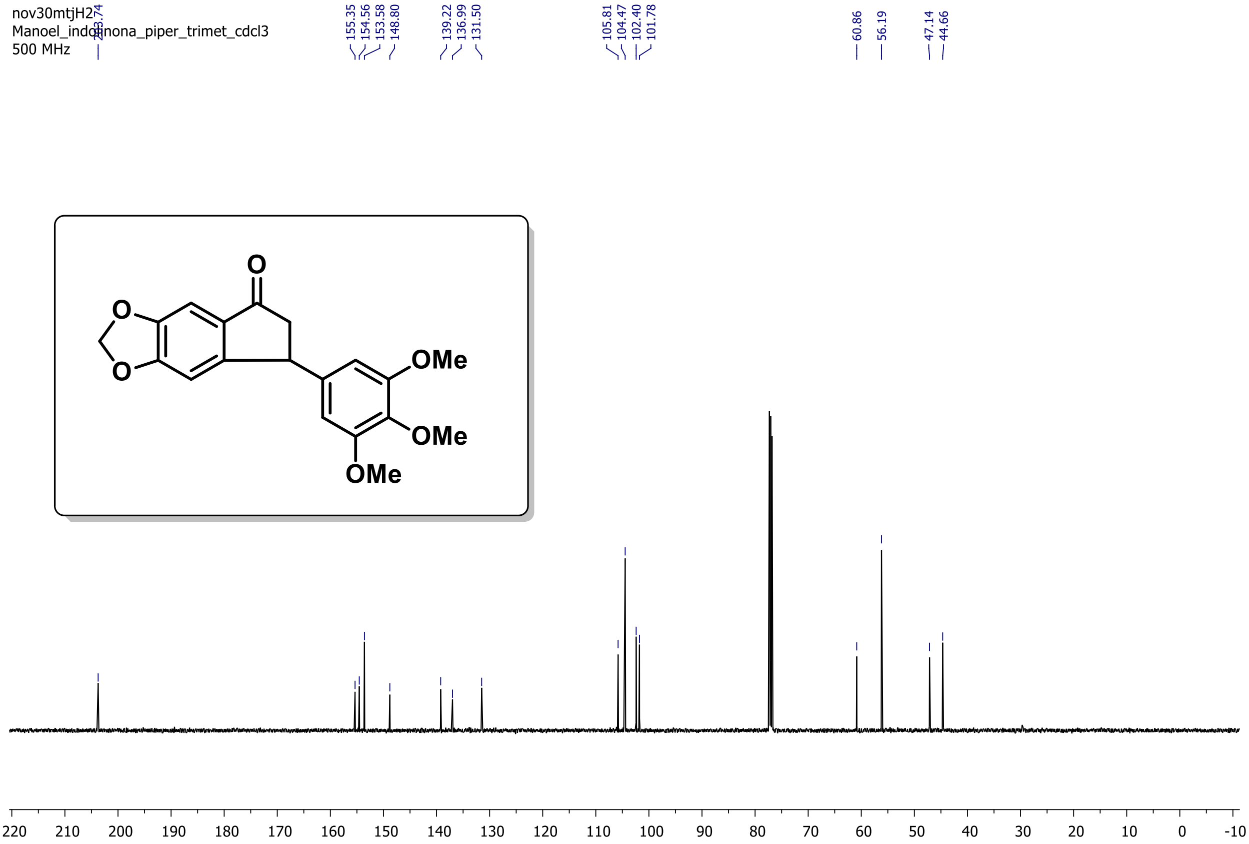

Figure S78. ^{13}C NMR spectrum (126 MHz, $CDCl_3$) of compound **11dc**.

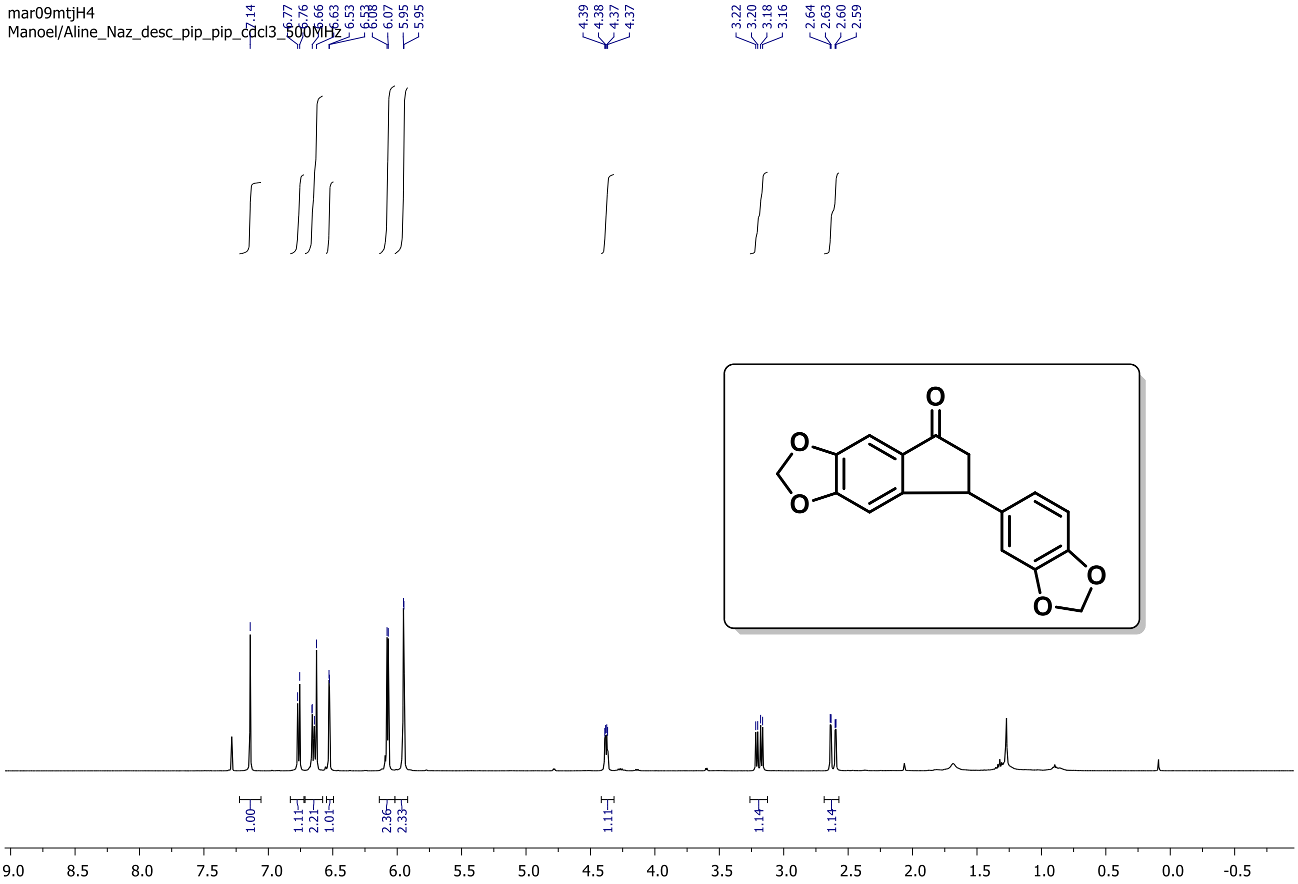

Figure S79. ^{1}H NMR spectrum (500 MHz, $CDCl_3$) of compound **11dl**.

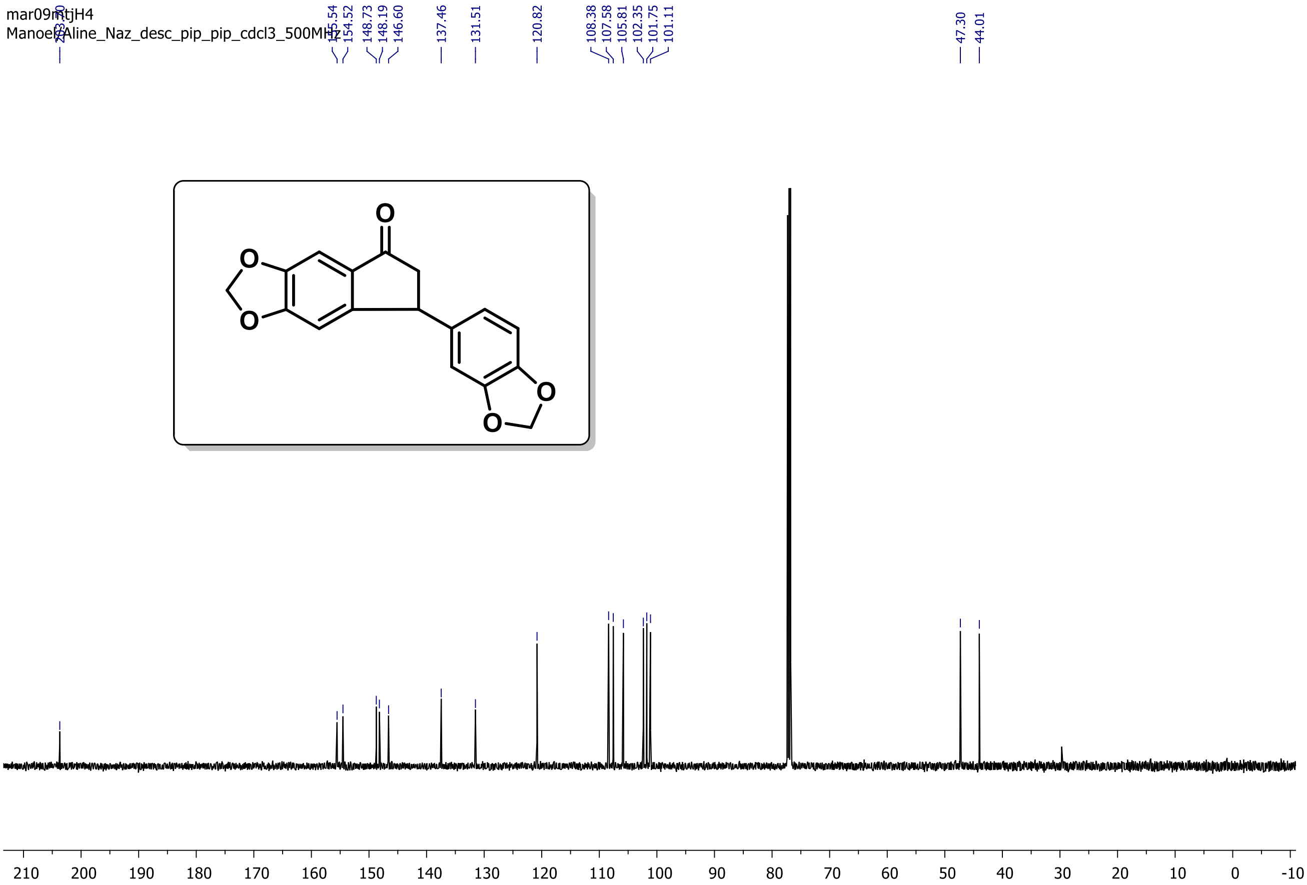

Figure S80. ^{13}C NMR spectrum (126 MHz, $CDCl_3$) of compound **11dl**.

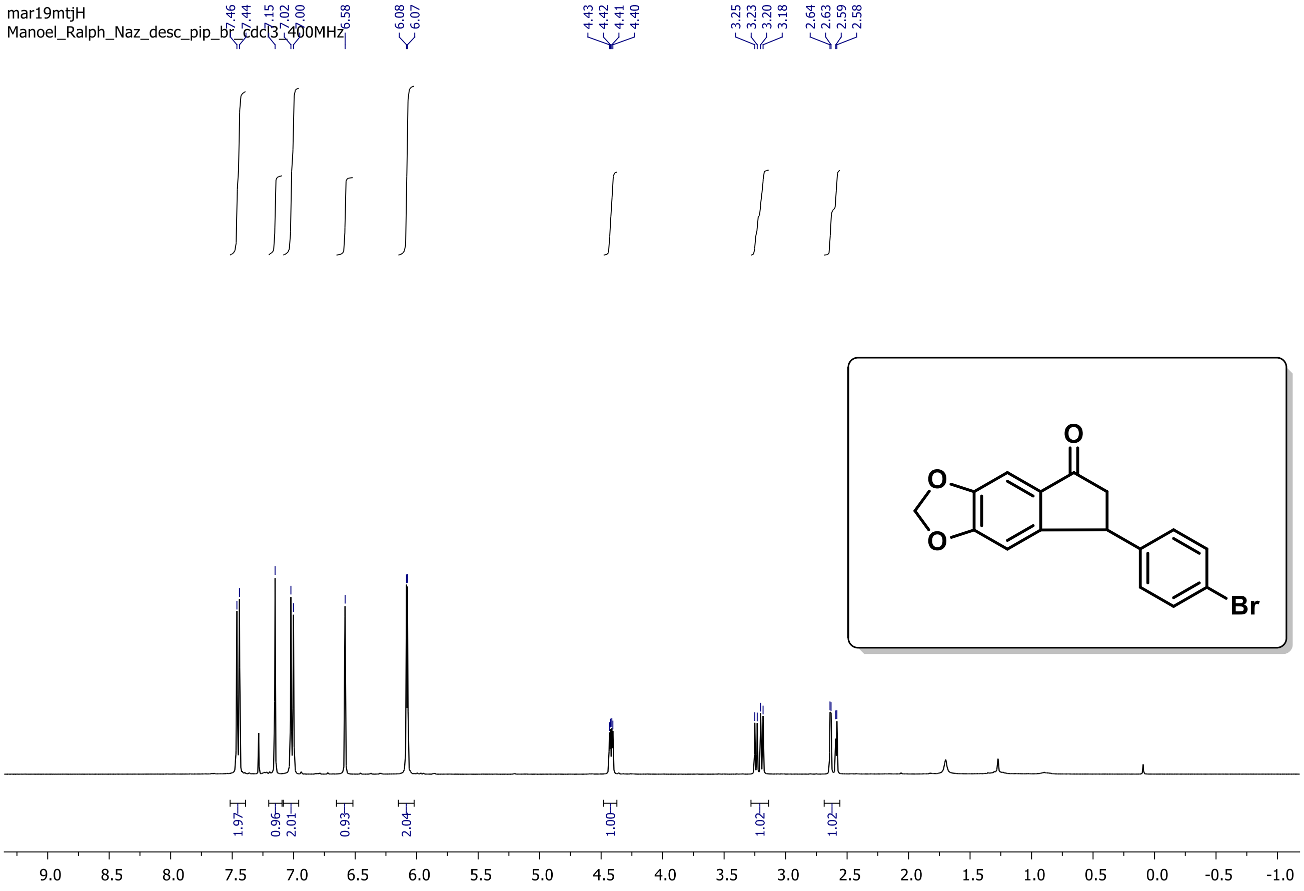

Figure S81. ^{1}H NMR spectrum (500 MHz, $CDCl_3$) of compound **11dm**.

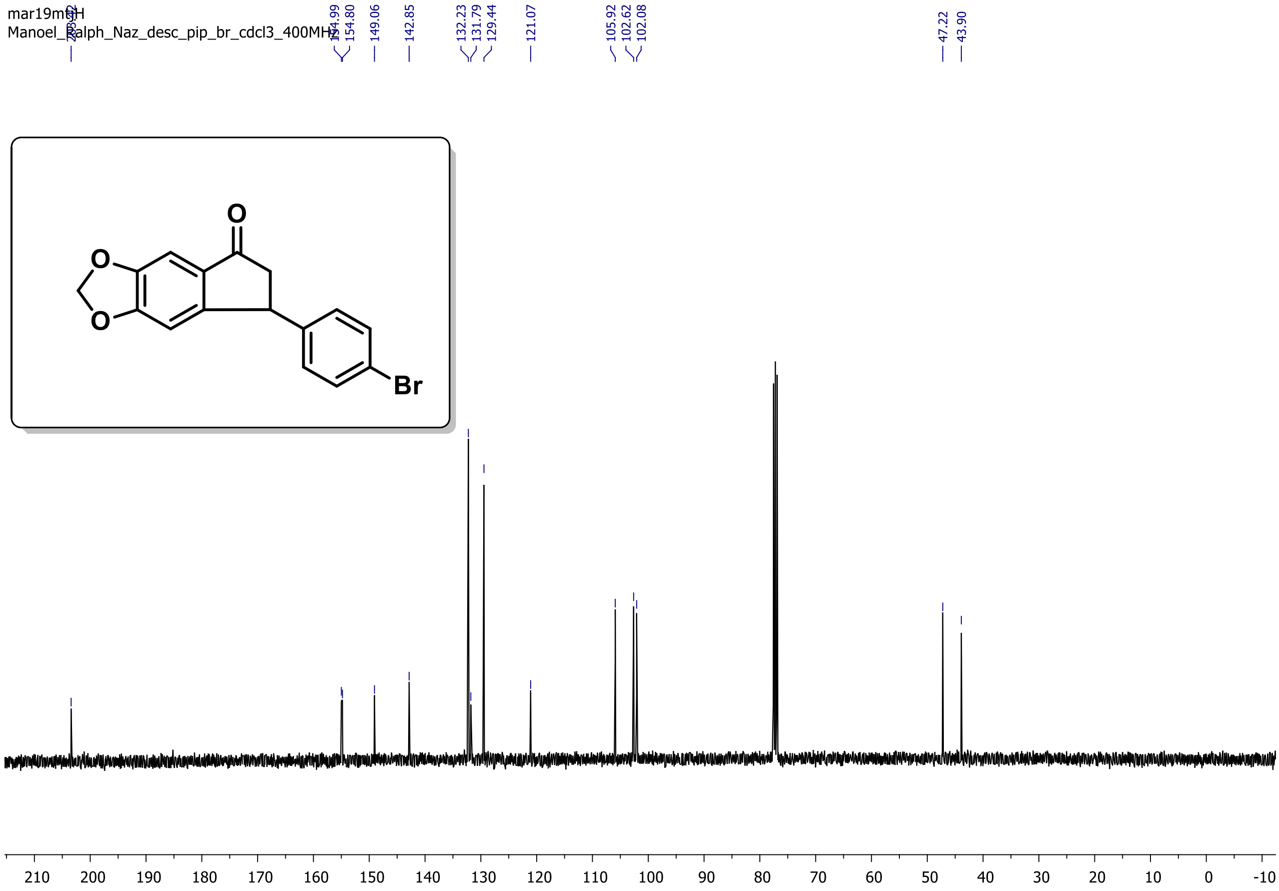

Figure S82. ^{13}C NMR spectrum (126 MHz, $CDCl_3$) of compound **11dm**.

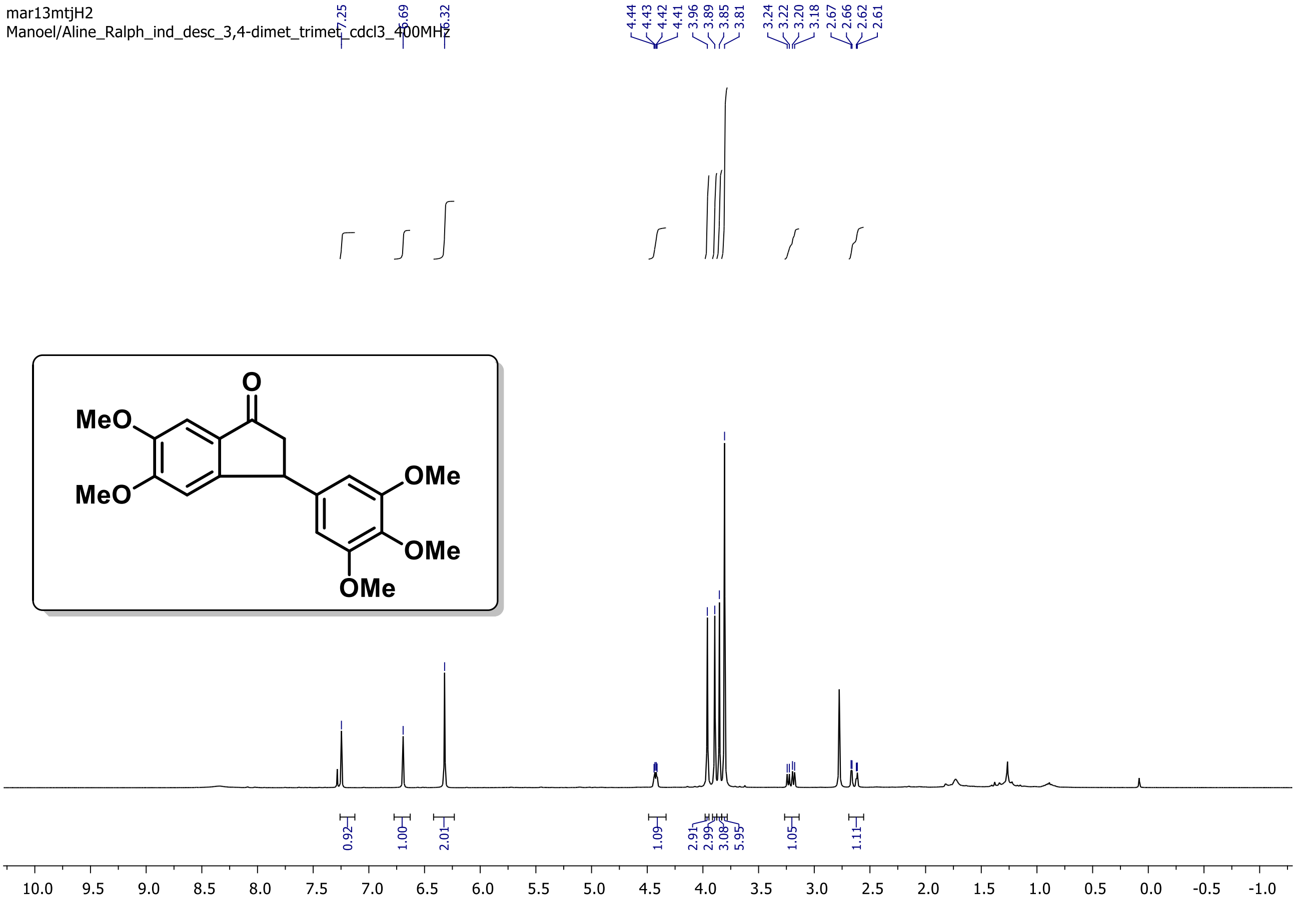

Figure S83. ^{1}H NMR spectrum (400 MHz, $CDCl_3$) of compound **11cc**.

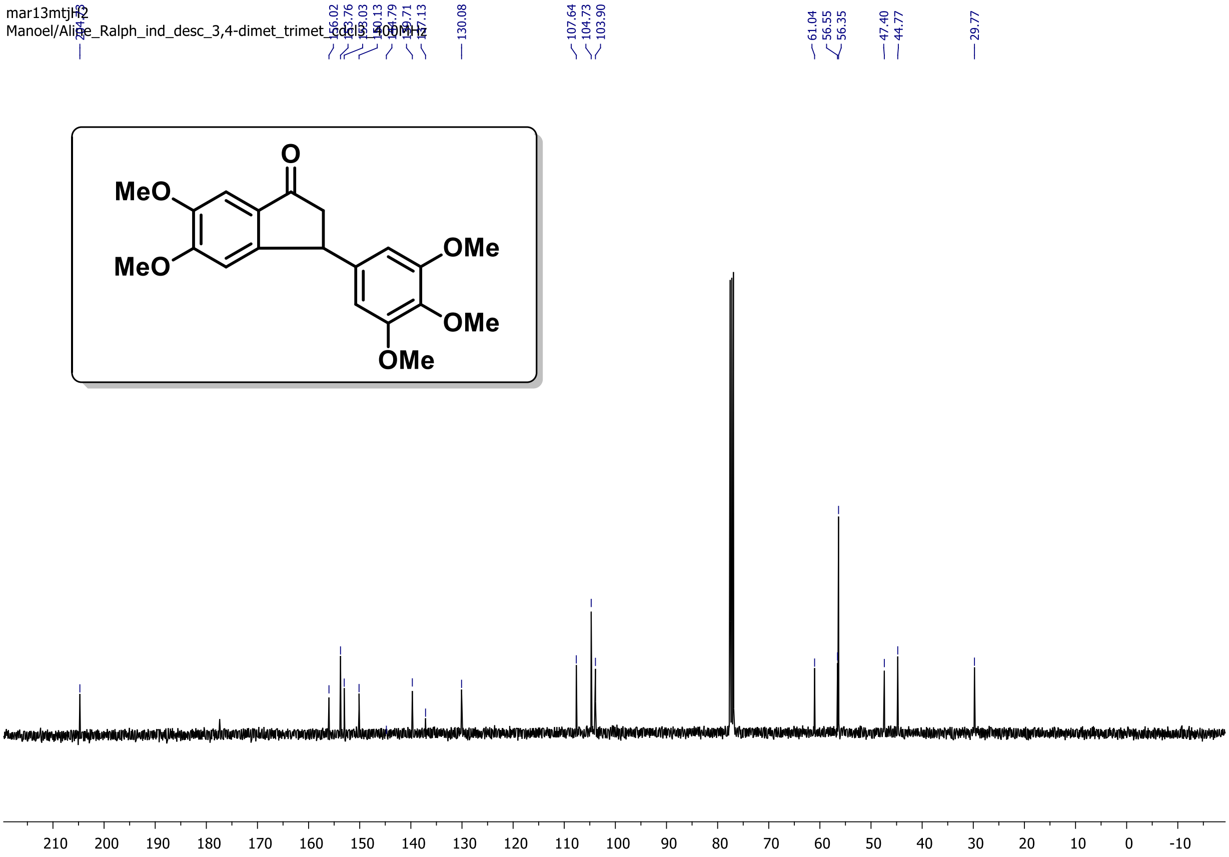

Figure S84. ^{13}C NMR spectrum (101 MHz, $CDCl_3$) of compound **11cc**.

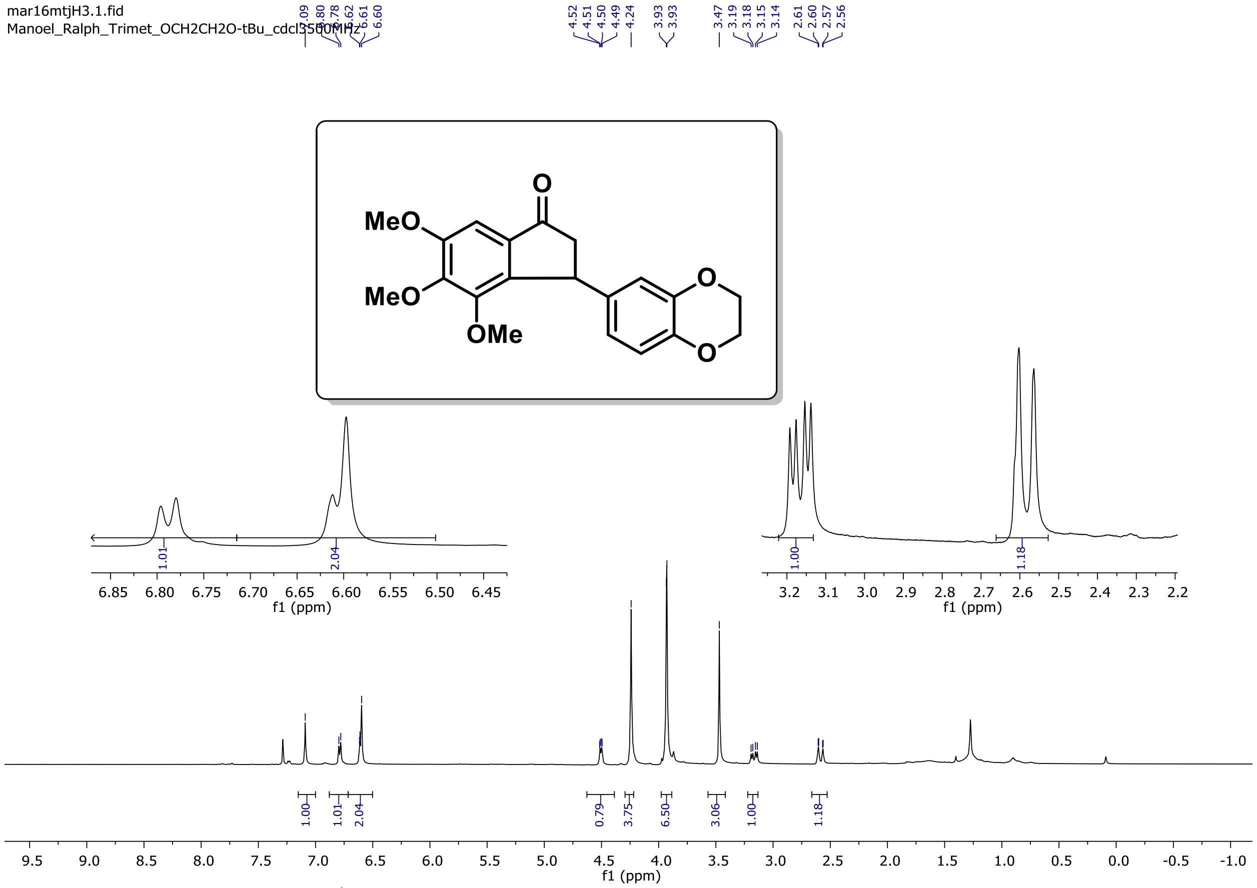

Figure S85. ^{1}H NMR spectrum (500 MHz, $CDCl_3$) of compound **11ae**.

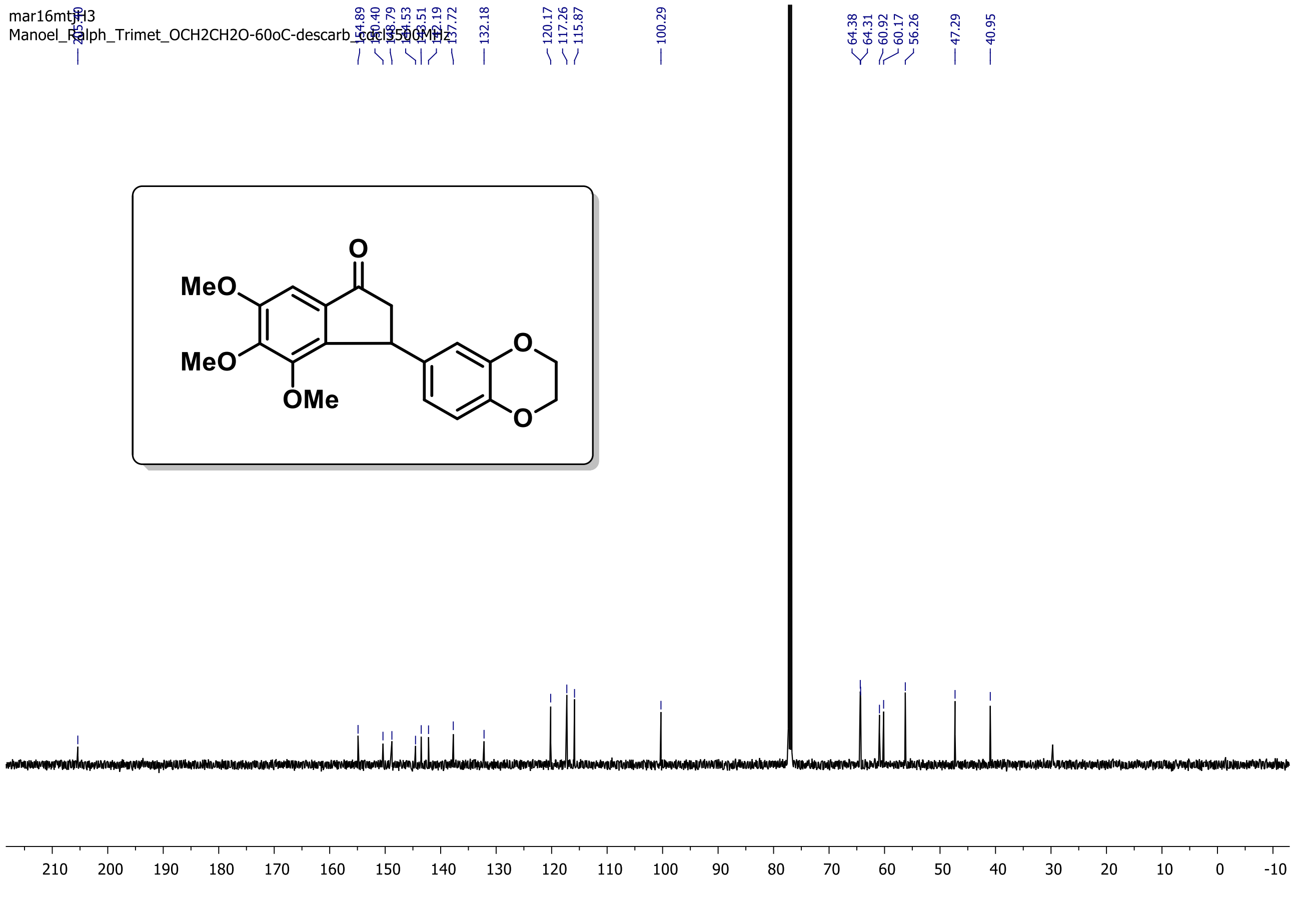

Figure S86. ^{13}C NMR spectrum (126 MHz, $CDCl_3$) of compound **11ae**.

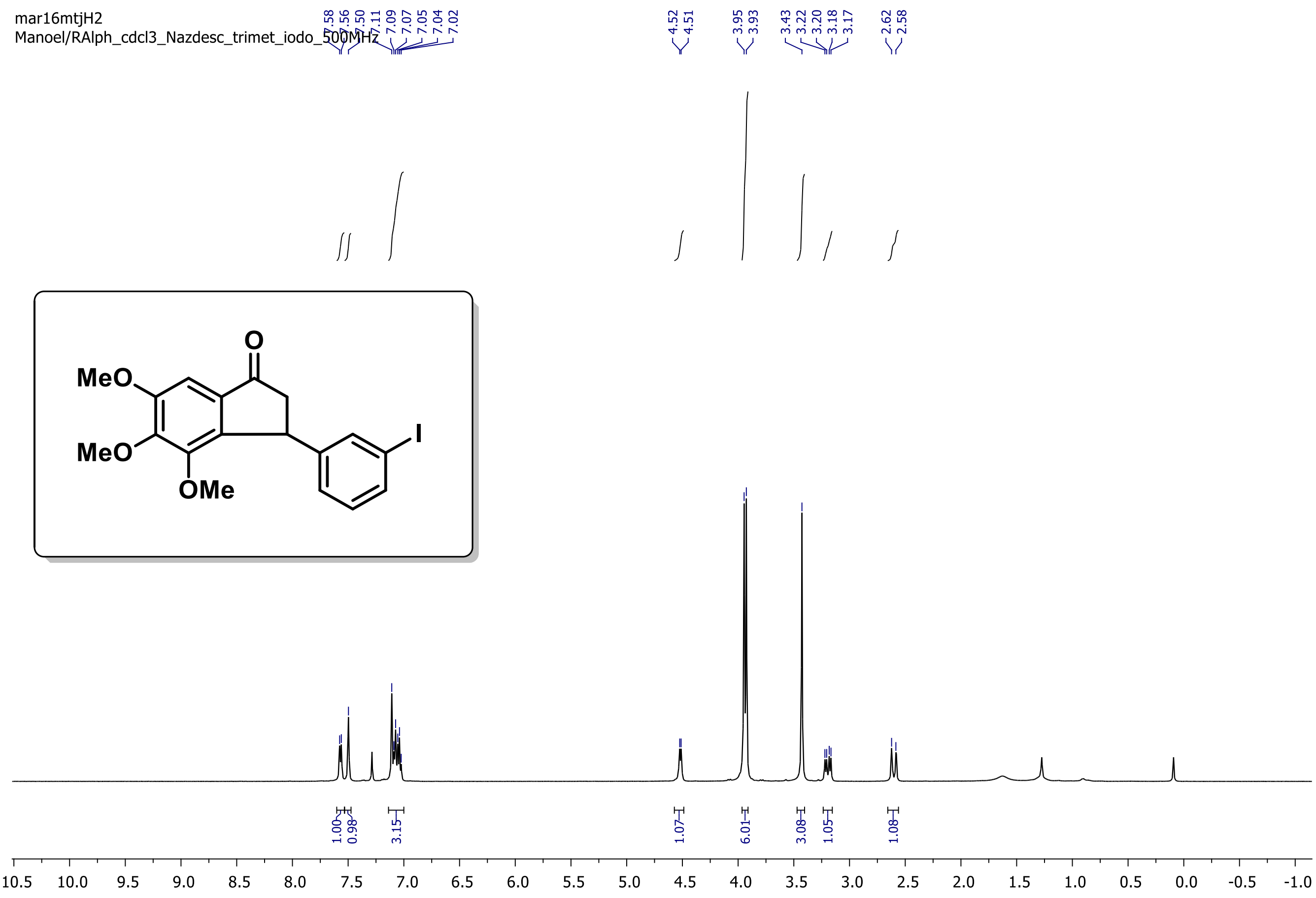

Figure S87. ^{1}H NMR spectrum (500 MHz, $CDCl_3$) of compound **11af**.

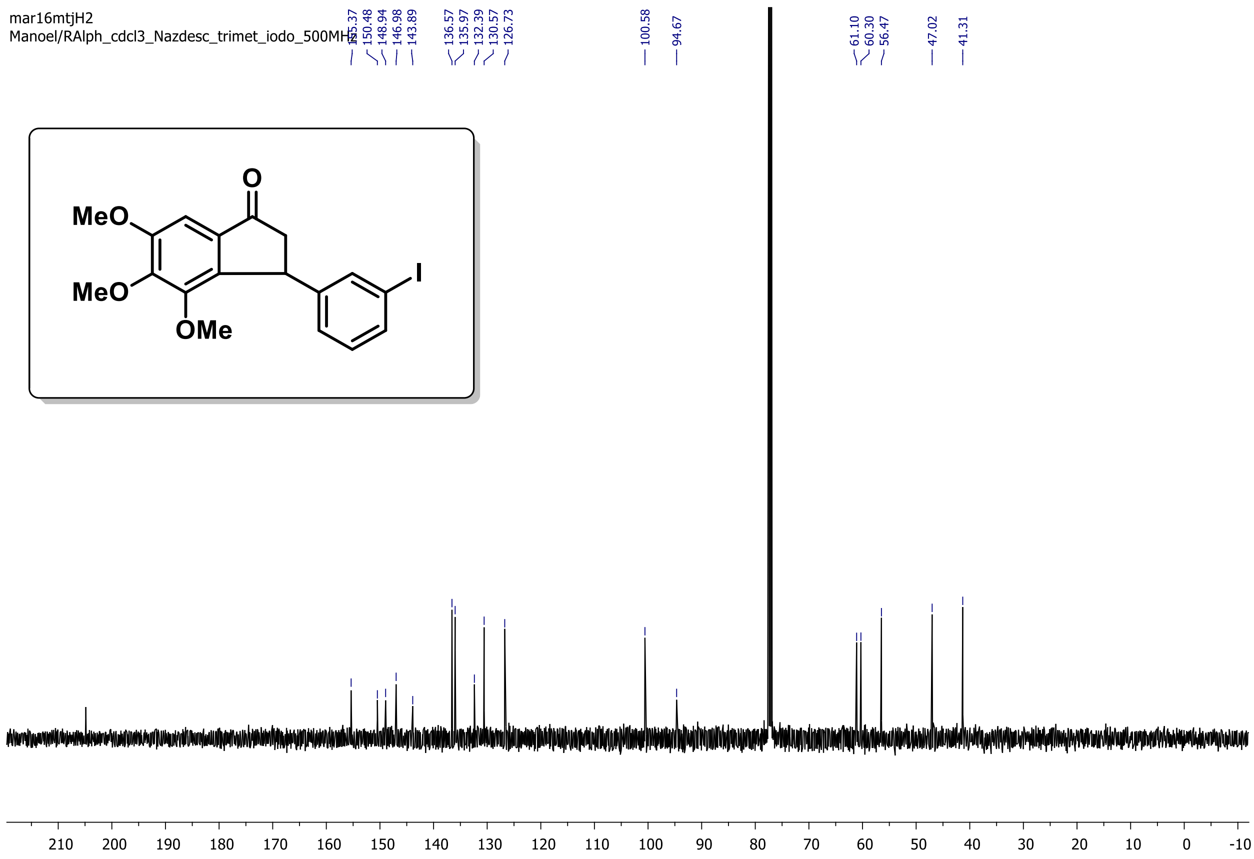

Figure S88. ^{13}C NMR spectrum (126 MHz, $CDCl_3$) of compound **11af**.
